# On the Homogeneity of a Cobalt-Based Water Oxidation Catalyst

**DOI:** 10.1021/acscatal.2c01299

**Published:** 2022-04-04

**Authors:** Daan den Boer, Quentin Siberie, Maxime A. Siegler, Thimo H. Ferber, Dominik C. Moritz, Jan P. Hofmann, Dennis G. H. Hetterscheid

**Affiliations:** † Leiden Institute of Chemistry, Leiden University, Einsteinweg 55, RA, Leiden 2300, The Netherlands; ‡ Department of Chemistry, Johns Hopkins University, 3400 North Charles Street, Baltimore 21218 Maryland, United States; § Surface Science Laboratory, Department of Materials and Earth Sciences, Technical University of Darmstadt, Otto-Berndt-Strasse 3, Darmstadt 64287, Germany

**Keywords:** water oxidation, oxygen evolution, electrocatalysis, surface characterization, homogeneous versus heterogeneous catalysis, mononuclear cobalt complex

## Abstract

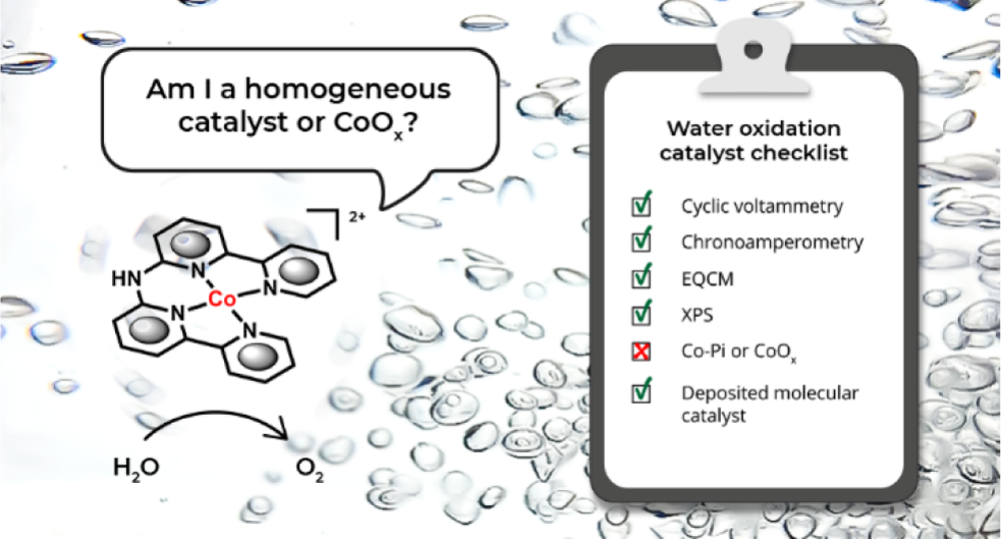

The homogeneity of molecular Co-based water oxidation catalysts (WOCs) has been a subject of debate over the last 10 years as assumed various homogeneous Co-based WOCs were found to actually form CoO_*x*_ under operating conditions. The homogeneity of the Co(H**L**) (H**L** = *N*,*N*-bis(2,2′-bipyrid-6-yl)amine) system was investigated with cyclic voltammetry, electrochemical quartz crystal microbalance, and X-ray photoelectron spectroscopy. The obtained experimental results were compared with heterogeneous CoO_*x*_. Although it is shown that Co(H**L**) interacts with the electrode during electrocatalysis, the formation of CoO_*x*_ was not observed. Instead, a molecular deposit of Co(H**L**) was found to be formed on the electrode surface. This study shows that deposition of catalytic material is not necessarily linked to the decomposition of homogeneous cobalt-based water oxidation catalysts.

## Introduction

The development of molecular catalysts for water oxidation has been a topic of interest for the last decades.^1^ The use of ruthenium complexes has been very useful to unravel the reaction mechanisms in which water oxidation catalysis occurs,^2−21^ while in particular iridium-based systems have been shown to have great robustness.^22−26^ However, the true active species and homogeneity of the latter iridium systems are not always very clear.^27−36^

Recently, the application of earth-abundant first-row transition metals, such as Cu, Fe, Ni, Mn, and Co, has gained a lot of interest. Compared to the highly active Ru- and Ir-based water oxidation catalysts (WOCs), these first-row transition metal complexes lack activity and stability, while thorough mechanistic studies have rarely been conducted.^37^

Cobalt-based WOCs, such as CoCl_2_, [Co(bpy)_3_]^3+^,[Co(NH_3_)_6_]^3+^, and colloidal cobalt hydroxide were reported since the early 80s and were studied in the presence of Ru photosensitizers.^38−49^ However, some of these assumed homogeneous Co-based systems were reported to form CoO_*x*_ particles during photochemical water oxidation.^47−49^ Notably, after the first publications of cobalt polyoxometalates (POMs) as active and oxidatively stable catalysts, the number of publications on Co-based WOCs has increased since 2010 onward.^50,51^ However, these publications on Co-based POMs have triggered a debate between mostly the Hill and Finke groups regarding the true active species for water oxidation in the presence of these Co-based POMs, which under specific conditions form heterogeneous CoO_*x*_ on the electrode surface.^52−65^ Such depositions of CoO_*x*_ on the electrode surface are known to be active for water oxidation catalysis themselves.^66−70^ The formation of CoO_*x*_ as an active catalyst is well studied by the group of Nocera, who showed the in situ generation of the very active Co-Pi WOC upon spontaneous deposition of cobalt oxide for various cobalt salts in a pH 7 phosphate buffer at the anode under operating conditions.^68,71−76^ It is therefore easy to imagine that unstable catalysts containing or liberating Co^2+^ ions in phosphate buffered solutions can readily form such active Co-Pi-type structures. Specific reaction conditions wherein catalysis was conducted proved to be a vital part of whether the operating species was most likely a CoO_*x*_ species or a truly homogeneous catalytic system. The research contributions of the Hill and Finke groups proposed situations regarding Co-based WOCs that one should consider when studying molecular Co-based WOCs:^64^

1. The Co-based WOC is a molecular catalyst.
2. There is a Co^2+^ impurity present, which forms CoO_*x*_ as the active catalyst.
3. The Co-based WOC releases Co^2+^, which forms CoO_*x*_ as the active catalyst.
4. The compound itself is a precursor for the formation of CoO_*x*_ as the active catalyst.
5. Another unidentified species is the true catalyst.
6. A combination of the abovementioned events occurs.

In the same period, a number of other Co-based systems were reported as molecular WOCs. The Berlinguette group reported pentadentate Co-pyridine systems as homogeneous catalysts for the water oxidation reaction.^77−79^ Later, the Anderson group reported very similar molecular complexes to be unstable during electrolysis, resulting in the formation of heterogeneous CoO_*x*_.^80^ The complex [Co^III^(hydroxydi(pyridin-2-yl)methanolate)_2_]^+^ was reported by Zhao et al. to be a light-driven WOC.^81^ Under electrochemical conditions, the very same catalytic species was shown to form Co-containing structures on the electrode surface, according to studies by the group of Najafpour.^82^ The compound [(TMPA)Co(μ-OH)(μ-O_2_)Co(TMPA)]^3+^ (TMPA = tris(2-pyridylmethyl)amine) was initially reported as the first dinuclear Co-based WOC.^83^ Reinvestigation of this catalytic system using surface characterization techniques revealed that water oxidation most likely is catalyzed by CoO_*x*_ at an overpotential of 550 mV.^84^ One year later, Kotani et al. claimed for the same catalytic system that water oxidation is catalyzed by a homogeneous species at an overpotential of 500 mV although no surface characterization techniques were employed in these studies.^85^ Also, several Co^III^_4_O_4_ cubane clusters were initially reported as molecular water oxidation catalysts.^86−90^ However, later it was shown for [Co^III^_4_O_4_(OAc)_4_(py)_4_] (py = pyridine) that Co^2+^ impurities in the cubane samples were actually the source of active species. The purified cubane complex was found to be completely inactive.^91^

Obviously, the assignment of the true active species in this field of research has to be taken with great care, and the precise reaction conditions for catalytic experiments are extremely important. Consequently, the application of surface-sensitive characterization techniques, such as X-ray photoelectron spectroscopy (XPS), has already become a standard in the field of homogeneous water oxidation catalysis to exclude the involvement of CoO_*x*_ in catalysis.^92−94^

The application of molecular catalysts allows for ligand design, which could help stabilize Co-based WOCs. Stabilization or circumvention of high-valent Co species seems to be one of the key design principles in the development of homogeneous Co-based WOCs, as it reduces the problem of Co^2+^ leaching or catalyst degradation with the formation of CoO_*x*_.^92,95−102^ The utilization of redox-active ligands can circumvent the formation of unstable high-oxidation state metal intermediates by storing redox equivalents on the ligand.^94,103,104^ Recently this strategy has already been effectively utilized for Ru-,^105^ Ni-,^106,107^ and Cu-based^108−110^ WOCs.

In line with these studies, we explored the water oxidation activity of a Co-based water oxidation catalyst bearing the redox-active tetradentate ligand *N*,*N*-bis(2,2′-bipyrid-6-yl)amine (H**L**), which was utilized previously in a Fe-based WOC.^111^ In addition, a catalytic system bearing the same structural motifs has recently been reported for ligand-assisted proton and CO_2_ reduction.^112,113^ In line with the aforementioned observations that CoO_*x*_ is easily formed under the catalytic conditions employed, we thoroughly and systematically investigated the homogeneity of the catalytic species during water oxidation catalysis. We present a unique case in which the active species neither remains in solution as a homogeneous species nor converts to the aforementioned CoO_*x*_ species that were shown to be the true active species in many previous studies.

## Results and Discussion

### Synthesis and Characterization

**Figure 1:**
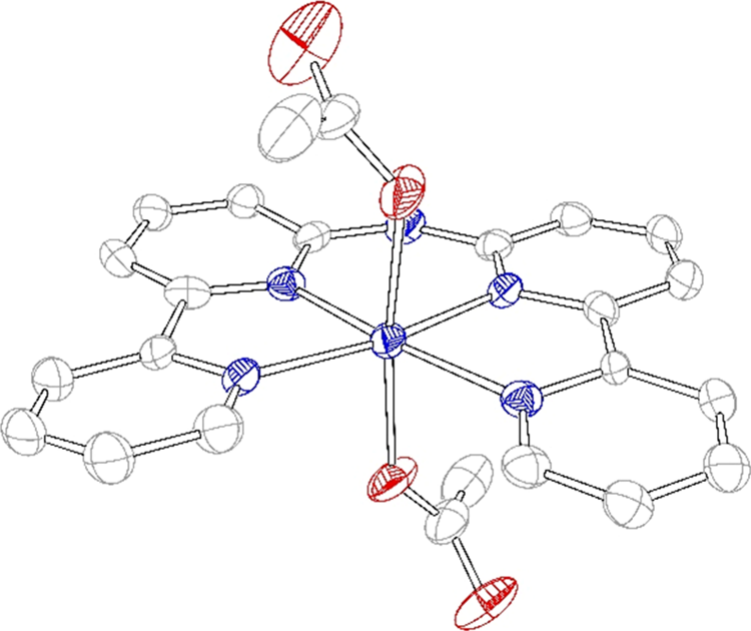
Displacement ellipsoid plot (50% probability level) of [Co(H**L**)(OAc)_2_] at 110(2) K. Disorder, a methanol molecule present in the lattice, and hydrogen atoms are not shown for clarity.

[Co(H**L**)(OAc)_2_] was synthesized by a reaction of cobalt(II) acetate and H**L** in methanol, and the product was purified by crystallization. Elemental analysis showed that the composition of the crystals is in good agreement with the structure [Co(H**L**)(OAc)_2_] without any residual solvent molecules present. The cobalt(II) ion of the Co(H**L**) crystal is found at a crystallographic inversion center in the asymmetric unit of the *P*1̅ space group (Figure 1). The [Co(H**L**)(OAc)_2_] molecule itself does not have an inversion center and, therefore, was found to be disordered (Figure S1). Bond lengths between the nitrogen donors of the tetrapyridyl ligand and the Co(II) ion vary in the range of 2.04–2.14 Å compared to 2.11–2.14 Å for the Fe-based analogue, respectively (Figures S2 and S3).^111^ Both acetate ions are coordinated to the Co(II) ion with Co–O bond lengths of 2.086(17) and 2.100(17) Å. The ligand H**L** surrounds the Co(II) ion in the equatorial plane with a torsion angle (N1–N2–N4–N5) of 2.63°, indicating that the ligand is in an almost perfect planar conformation.

The magnetic moment of [Co(H**L**)(OAc)_2_] in the solid state was determined to be 4.29 μB, indicating a high-spin cobalt(II) species with three unpaired electrons.^114^ The ^1^H NMR spectrum of Co(H**L**) in D_2_O shows a signal for the free acetate ion in the diamagnetic region, indicating that upon dissolving [Co(H**L**)(OAc)_2_] in water, the coordinated acetate ions are readily exchanged by water molecules, resulting in [Co(H**L**)(H_2_O)_2_]^2+^ (Figure S4). The color of an aqueous solution of Co(H**L**) changes reversibly from yellow to purple when the solution is cooled to −78 °C, while this color transition was not observed for the solid material [Co(H**L**)(OAc)_2_] (Figure S5).

UV–vis spectroscopy was used to study the stability and determine the p*K*_a_ of [Co(H**L**)(OAc)_2_] dissolved in water. UV–vis absorbance spectra were recorded in Milli-Q water, and four absorbance bands were found at 232, 254, 281, and 342 nm. Co(H**L**) was found to be stable in Milli-Q water of neutral pH for at least 6 days (Figure S6). Upon addition of base, the UV–vis spectra changed significantly. The four previously mentioned absorbance bands partially diminish, and two new absorbance bands arise at 337 and 418 nm. By a UV–vis monitored titration of the solution with NaOH, a p*K*_a_ of 10.2 was found, which we relate to deprotonation of the secondary amine in Co(H**L**) (Figures S9 and S10).

### Cyclic Voltammetry

**Figure 2:**
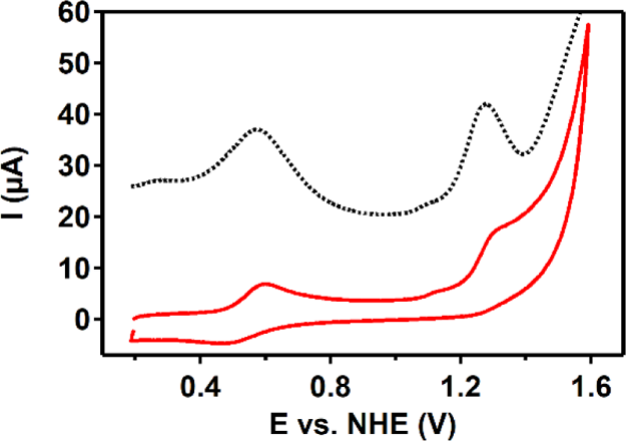
CV (red) and differential pulse voltammogram (black dotted) of 0.5 mM Co(H**L**) in a 100 mM pH 7 phosphate buffer at a scan rate of 100 mV/s. Glassy carbon (GC), Au, and a reversible hydrogen electrode (RHE) were used as the working electrode (WE), counter electrode (CE), and reference electrode (RE), respectively. Potentials were converted to the NHE.

A cyclic voltammogram (CV) of Co(H**L**) was recorded in a pH 7 phosphate buffer (Figure 2). A reversible redox wave is found, at 0.54 V versus normal hydrogen electrode (NHE), assigned to the Co^II/III^ redox couple. With a 100 mV difference between E_pc_ and E_pa_, the Co^II/III^ redox couple is quite broad for a one-electron process.^115^ The currents of the oxidative and reductive wave of the Co^II/III^ redox couple depend linearly on the square root of the scan rate, which is in good agreement with a diffusive process (Figures S11 and S12).^115^ In addition, an irreversible oxidative wave is found at 1.28 V versus NHE, followed by a catalytic wave. Differential pulse voltammetry (DPV) showed an irreversible redox wave as well in addition to the Co^II/III^ waves (Figures 2 and S13). The CV of the Zn-analogue, Zn(H**L**), in a pH 7 phosphate buffer also shows an irreversible oxidative wave at 1.28 V versus NHE (Figure S14), suggesting that the ligand is redox-active at this potential.

To investigate if Co(H**L**) is a homogeneous catalyst or acts as a Co^2+^ source for Co-Pi formation, the electrocatalytic data obtained with Co(H**L**) were compared with those obtained with Co-Pi that was deliberately made from Co(NO_3_)_2_ in a sodium phosphate buffer according to reported methods.^68^ A CV of Co(NO_3_)_2_ forming Co-Pi that was recorded under the same conditions as Co(H**L**) shows an oxidative wave at ∼1.15 V versus NHE, after which a catalytic wave arises (Figures S15 and S16). These features were also reported in the literature.^68^ For Co–Pi, a 10 times higher current at 1.6 V versus NHE was found than for Co(H**L**), indicating that Co–Pi is the more active electrocatalyst. The irreversible oxidative wave at 1.28 V versus NHE for Co(H**L**) and oxidative wave at 1.15 V versus NHE for Co–Pi do not overlap, indicating that the electronic structures of Co(H**L**) and Co–Pi are different (Figure S16). However, a minor oxidative current is observed at 1.1 V versus NHE in both the CV and the DPV of Co(H**L**).

### Pourbaix Diagram

**Figure 3:**
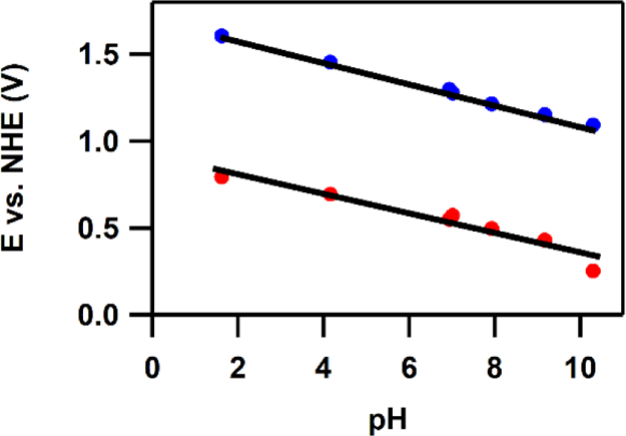
Pourbaix diagram of Co(H**L**).

Cyclic and differential pulse voltammograms of 0.5 mM Co(H**L**) were recorded for solutions containing 100 mM phosphate as an electrolyte in the pH window between pH 1 and 10, and the potential of the two oxidative waves are plotted as a function of pH in a Pourbaix diagram (Figure 3). In this pH range, the potentials of the two redox events change with a slope of −59 and −61 mV/pH, respectively; thus, both oxidations can be assigned to a proton-coupled electron transfer (PCET) process. After the two PCET steps, an active species for water oxidation is obtained as we observe a catalytic wave in the CV (Figure 2). When the pH of the solution is increased above pH 10, the Co^II/III^ redox couple starts to disappear, and at pH 13, this redox couple is completely absent (Figure S17). Moreover, the catalytic currents increase 10-fold over 15 cycles in the cyclic voltammetry experiment. This suggests the formation of a CoO_*x*_ deposit at the electrode surface and thus instability of the homogeneous (pre)-catalyst. We have, therefore, refrained from further studies under these ill-defined alkaline conditions.

### Dipping Tests

The homogeneity and stability of Co(H**L**) was further investigated at pH 7, where Co(H**L**) shows a catalytic activity that is relatively high compared to other pH values in the range of 0 to 10.

Electrode dipping tests were conducted by comparing CVs in the potential window of 0.2–1.6 V versus NHE. For this, CVs recorded in the presence of Co(H**L**) were compared to experiments in the blank solution in a separate electrochemical cell containing only phosphate buffer, recorded before and after each experiment in the presence of Co(H**L**). During transfer between cells the electrode surface was rinsed with water to remove any remaining Co(H**L**)-containing droplets. Different potential windows were used in these dipping experiments to be able to relate deposition to individual redox processes.

First, the Co^II/III^ redox couple is discussed. A CV of the blank solution was recorded between 0.2 and 1.6 V versus NHE followed by a CV of 10 cycles recorded in a solution containing Co(H**L**) in a potential window of 0.2 to 0.7 V versus NHE in which the Co^II/III^ redox couple is found (*E*_1/2_ = 0.54 V versus NHE). Compared to the blank CV prior to contact with Co(H**L**), the blank recorded afterward reached a marginal higher current at 1.6 V versus NHE (Figure S18), pointing to some minor deposition. However, after 10 cycles in the blank solution, the current at 1.6 V versus NHE decreases to currents typically found prior to exposure to Co(H**L**) (Figure S19).

**Figure 4:**
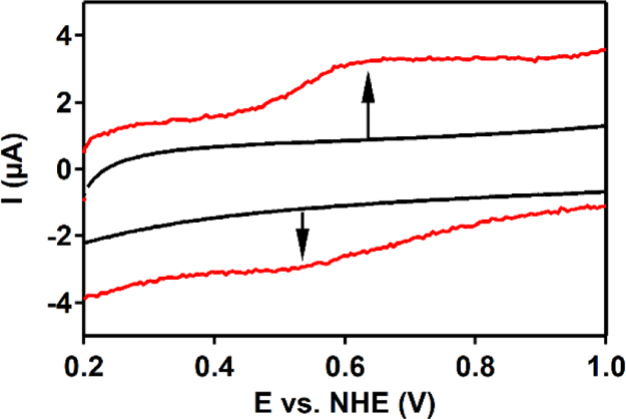
CV in a 100 mM pH 7 phosphate buffer at a scan rate of 100 mV/s before (black) and after (red) the electrode was cycled 75 times between 0.2 and 1.6 V versus NHE in a 0.5 mM Co(H**L**) in a 100 mM pH 7 phosphate buffer at a scan rate of 100 mV/s. GC, Au, and RHE were used as WE, CE, and RE, respectively. Potentials were converted to NHE.

Whereas the current enhancement upon exposure to Co(H**L**) in the 0.2–0.7 V versus NHE potential window results in a marginal increase in currents in the postcatalysis blank solution, the exposure to Co(H**L**) in the potential window between 0.9 and 1.6 V versus NHE led to significant currents. In the presence of Co(H**L**), a catalytic current of ∼40 μA is obtained at 1.6 V versus NHE in the first scan, which upon prolonged potential cycling increases to a catalytic current of 120 μA in the 75th scan. The first scan in the postcatalysis blank reached a current of 60 μA at 1.6 V versus NHE, which is half of the final catalytic current found after 75 scans in the presence of Co(H**L**) (Figure S20). During potential cycling in the postcatalysis blank solution, the obtained current at 1.6 V versus NHE gradually decreased scan-by-scan from 60 to 30 μA after 50 cycles (Figures S21 and S22). However, the current at 1.6 V versus NHE remained significantly higher than the currents before catalysis in the blank solution. Very similar results are obtained when the WE is cycled between 0.2 (instead of 0.9 V) to 1.6 V versus NHE in solutions containing Co(H**L**). While the potential is cycled for 75 scans in the Co(H**L**) solution, the Co^II/III^ redox couple, which was ascribed to a free diffusive process, remains visible during the entire series of 75 scans (Figures S23–S25). Also, in the postcatalysis scans, the presence of a reversible redox wave around 0.55 V versus NHE remains visible for multiple scans (Figure 4). When the postcatalysis run was carried out in a pH 2.5 solution, the oxidative wave of the redox couple shifts to 0.85 V versus NHE (Figure S26), which is in good agreement with the Co^II/III^ redox couple of Co(H**L**) in the Pourbaix diagram (Figure 3). This is a strong indication that the deposited material largely consists of Co(H**L**).

In other postcatalysis experiments, the deposits obtained from Co(H**L**) and Co-Pi on the WE were investigated with chronoamperometry and cyclic voltammetry and compared. After 75 cycles, in the presence of either Co(H**L**) or Co(NO_3_)_2_ between 0.9 and 1.6 V versus NHE, a deposit formed on the WE was studied in the blank solution by using chronoamperometry at 1.29 V versus NHE for 30 min (Figure S27). Here, Co-Pi showed significantly more current than the deposited Co(H**L**). Additionally, the deposit formed after 75 cycles in the presence of either Co(H**L**) or Co-Pi between 0.9 and 1.6 V versus NHE was investigated under noncatalytic conditions using cyclic voltammetry in MeCN. Using the electrode that was exposed to Co(H**L**), an oxidative and a reductive wave was found between −0.1 and 0.0 V versus Fc/Fc^+^ (Figure S28). These waves, also occurring under catalytic conditions (Figure 4), most likely correspond to the Co^II/III^ redox couple. In the case of Co-Pi, an oxidative wave is found between 0.4 and 0.6 V and a reductive wave around 0.4 V versus Fc/Fc^+^ (Figure S29). As the postcatalysis redox waves for Co(H**L**) and Co-Pi are found at different potentials, the deposits evidently are different from each other.

### Electrochemical Quartz Crystal Microbalance

The formation of catalytically active deposits during cyclic voltammetry and chronoamperometry was further investigated using electrochemical quartz crystal microbalance (EQCM) experiments. The combination of electrochemistry with the quartz crystal microbalance allows for the detection of mass changes on the Au electrode surface due to redox triggered events by monitoring changes in the Δ frequency (Δ*f*) of the quartz crystal on which the thin Au layer is deposited. The accumulation of mass onto the EQCM electrode is inversely proportional to changes in the Δ*f*. In these experiments, a Δ*f* of 1.0 Hz corresponds to deposition of 12 ng/cm^2^ material (Figure S30 shows the calibration of the EQCM).

**Figure 5:**
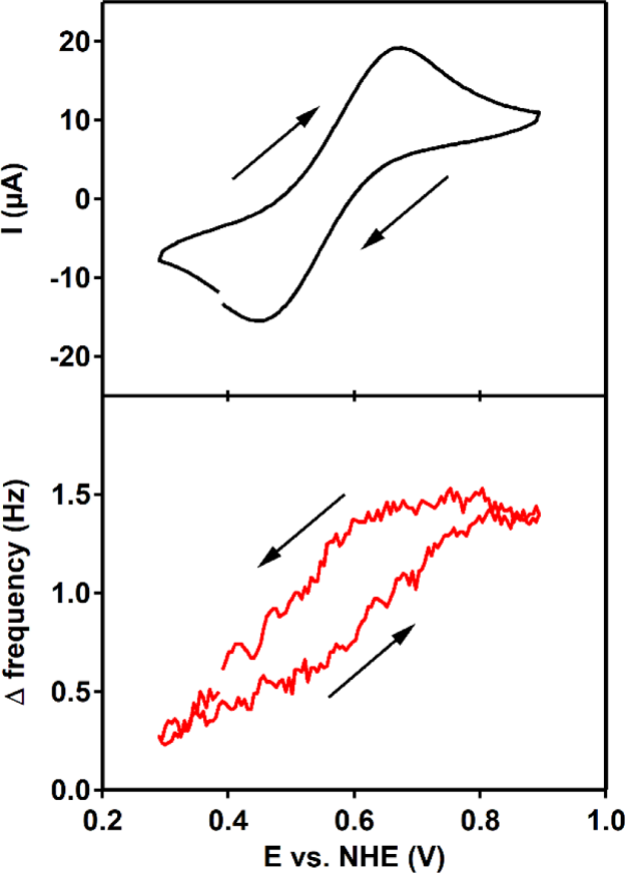
CV in combination with EQCM. Top: CV of 0.5 mM Co(H**L**) in a 100 mM pH 7 phosphate buffer at a scan rate of 100 mV/s. Bottom: Δ frequency response. Au, Au, and RHE were used as the WE, CE, and RE, respectively. Potentials were converted to NHE.

The Co^II/III^ redox couple of Co(H**L**) is discussed first. A minor increase in Δ*f* response was found upon oxidation of Co^II^ to Co^III^, and a similar decrease in Δ*f* was observed upon reduction of Co^III^ to Co^II^ (Figure 5). These reversible changes suggest that Co^II^(H**L**) precipitates on the electrode surface but, upon oxidation to Co^III^(H**L**), dissolves in solution. Overall, there is no net Δ*f* change over 10 cycles (Figure S31). The Δ*f* changes connected to the Co^II^/Co^III^ redox couple are in line with the relatively large Δ*E* of 100 mV in the CV and with small amounts of the cobalt compound remaining on the electrode surface in the dipping tests in the same potential window. The observed Δ*f* of roughly 1 Hz corresponds to adsorption of less than 1 monolayer of Co(H**L**). This interaction between Co^II^(H**L**) and the electrode might be explained by the geometry of the complex, as the ligand has an almost perfect planar structure, with a torsion angle of 2.63° and a fully conjugated π-system, the planar conjugated π-system could interact with the electrode surface.

**Figure 6:**
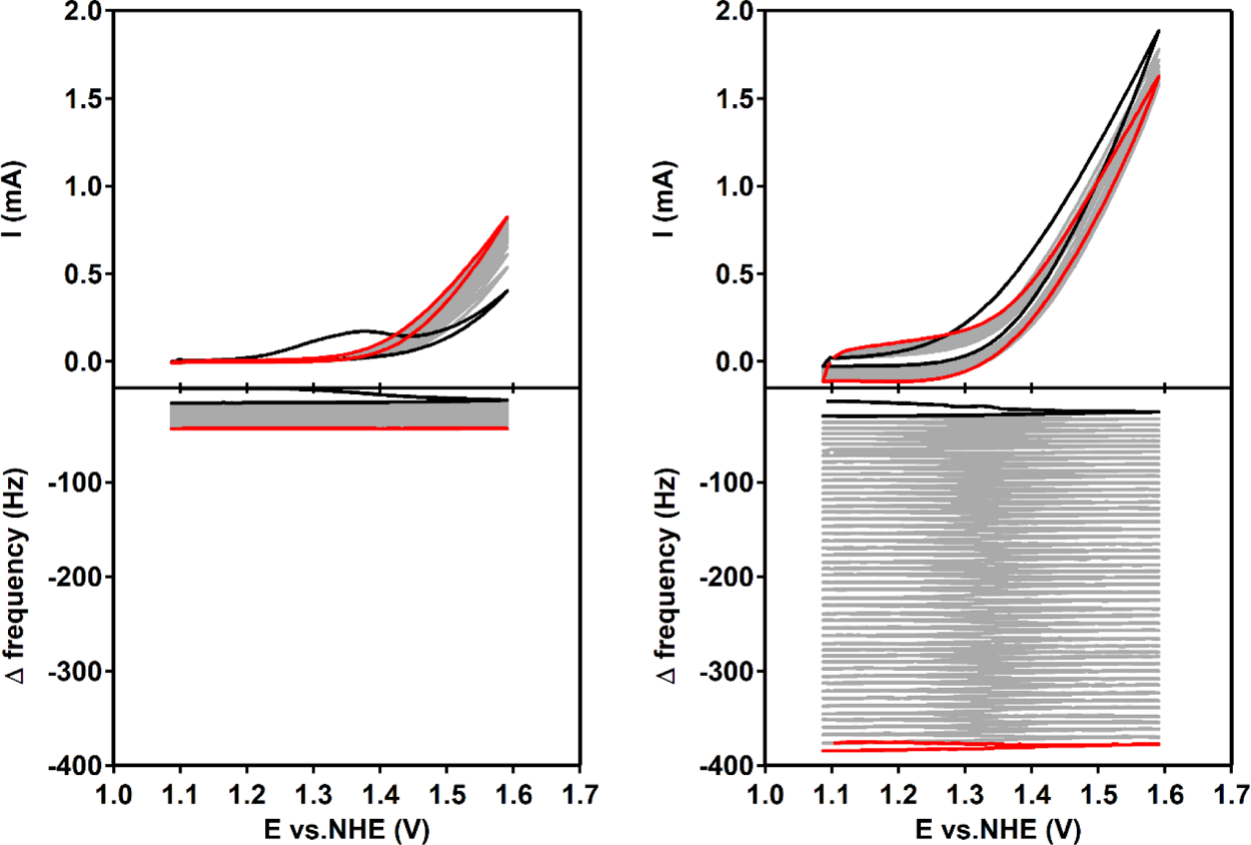
CVs in combination with EQCM of Co(H**L**) on the left and Co(NO_3_)_2_ on the right. In both graphs, cycle 1 is depicted in black and cycle 50 in red. Top: CV of 0.5 mM [Co] in a 100 mM pH 7 phosphate buffer at a scan rate of 100 mV/s. Bottom: Δ frequency response. Au, Au, and RHE were used as the WE, CE, and RE, respectively. Potentials were converted to NHE.

Secondly, the catalytic wave observed with cyclic voltammetry in combination with EQCM is discussed. In this experiment, 50 cycles were recorded between 1.1 and 1.6 V versus NHE (Figures 6 and S31 for zoom in). In the case of Co(H**L**), the Δ*f* decreases scan-by-scan, with larger Δ*f* steps in the first scans (Figure S32). After 50 cycles, the Δ*f* corresponds to adsorption of multiple layers of presumably Co(H**L**). In comparison, using a solution containing Co(NO_3_)_2_, around a 10-fold larger change in Δ*f* is observed, indicating that 10 times more material is deposited on the electrode surface. Moreover, the Δ*f* decreases stepwise with a constant value in every scan showing a continuous build-up of Co–Pi when using a solution containing Co(NO_3_)_2_.

### Exploring the Role of Free Co^2+^

It has been reported for Co^III^_4_O_4_(OAc)_4_(Py)_4_ that free Co^2+^ ions present in the samples are the source of the true active species rather than the Co^III^_4_O_4_ complex.^91^ The presence of free Co^2+^ ions can be ruled out in solution by using either ^31^P NMR experiments or trapping experiments with the addition of ethylenediaminetetraacetic acid (EDTA), following literature procedures.^91^

It is known that free Co^2+^ ions can bind to phosphate ions, resulting in a significant line broadening of the phosphate resonance peak in the ^31^P NMR spectrum, which is easily recognized (0.1 M solution of pH7, Figure S33). However, Co(H**L**) can also bind to phosphate ions, which causes a small shift in the ^31^P NMR spectrum from 2.35 to 2.60 ppm with a significantly less line broadening compared to equivalent molar amounts of Co(NO_3_)_2_ (Figures S34 and S35). However, spiking a 500 μM solution of Co(H**L**) with up to 5 μM concentrations of Co(NO_3_)_2_ did not lead to significant changes in the ^31^P NMR spectrum of phosphate solutions. Although this experiment rules out that large quantities of free Co^2+^ ions are spontaneously formed upon dissolving [Co(H**L**)(OAc)_2_] in aqueous solutions, we cannot rule out the presence of relatively small amounts of free Co^2+^.

Free Co^2+^ ions strongly bind to EDTA, which is a known inhibitor of Co-Pi formation. Loss of catalytic activity upon addition of EDTA to a solution is thus an indication that the catalytic activity is caused by Co-Pi formed from free Co^2+^ions. The addition of 5 up to 20% amounts of EDTA to a 0.5 mM Co(H**L**) solution did not lead to any suppression of the catalytic current, in contrast to what has been reported for a Co-cubane compound.^91^ Instead, the current increases slightly upon the addition of higher amounts of EDTA (Figure S36), which is probably due to some of the EDTA being electrochemically oxidized (Figures S37 and S38). These results suggest that the deposition process does not proceed via free Co^2+^ ions in solution but rather that the complex as a whole or decomposition products of the complex are the cause of the observed frequency changes in the EQCM experiments.

### Chronoamperometry

**Figure 7:**
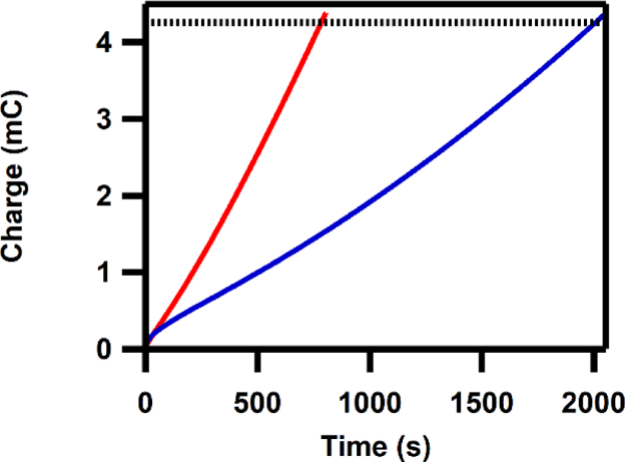
Total passed charge versus time upon applying 1.25 V versus NHE on a solution of 0.5 mM Co(H**L**) (blue line) and 0.5 mM Co(NO_3_)_2_ (red line) in a 100 mM pH 7 phosphate buffer solution. The dotted black line corresponds to a total passed charge of 4.26 mC, corresponding to 60 mC/cm^2^. GC, Au, and RHE were used as WE, CE, and RE, respectively.

In order to compare a deposit formed from Co(H**L**) with Co-Pi, deposits of both were prepared via the same method based upon a reported procedure for Co-Pi film formation.^74^ A potential of 1.25 V versus NHE was applied until 60 mC/cm^2^ of charge has passed (this corresponds to 4.26 mC for the electrode used in our experiments). Prior to the experiments, the GC electrode was anodized at 2.1 V versus NHE to fully oxidize the electrode surface and diminish the background effects of the GC electrode.^35^ In the first 50 s, the total charge increase was identical for both compounds. For the solution containing Co(H**L**), it took 2.5 times longer to reach 4.26 mC (Figure 7).

Both electrodes containing either the Co-Pi or the Co(H**L**) deposit were evaluated with cyclic voltammetry in a blank phosphate solution. The electrode with Co-Pi was significantly more active for water oxidation than the electrode with a deposit prepared from a Co(H**L**) solution (Figures S39 and S40). However, in both cases the current diminished with an increasing number of cycles. In the case of Co–Pi, an oxidative wave is clearly visible prior to the catalytic wave, which shifts to a higher potential every cycle (Figure S38). This might be caused by local pH effects, sintering, or Ostwald ripening of the deposit. In contrast, such oxidative waves were not found in the case of deposited Co(H**L**).

**Figure 8:**
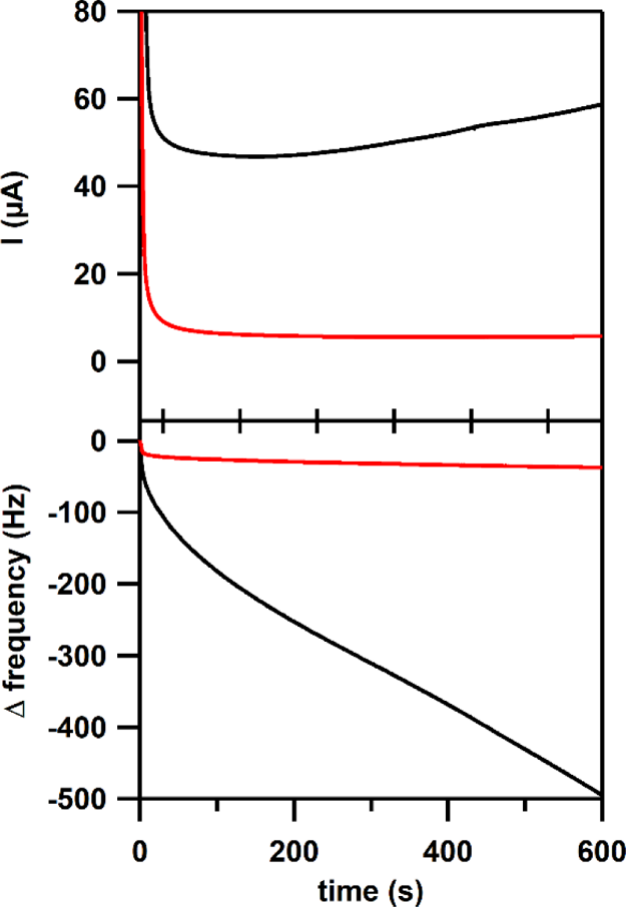
Chronoamperometry in combination with EQCM of Co(H**L**) (in red) and Co(NO_3_)_2_ (in black). Top: chronoamperogram upon applying 1.29 V versus NHE in a 0.5 mM [Co] in a 100 mM pH 7 phosphate buffer. Bottom: Δ frequency response over time. Au, Au, and RHE were used as the WE, CE, and RE, respectively.

In another experiment, EQCM was combined with chronoamperometry by applying 1.29 V versus NHE for 600 s, while the Δ*f* is monitored. The obtained current in the chronoamperogram for the solution containing Co(NO_3_)_2_ is an order of magnitude higher than for the solution containing Co(H**L**). Furthermore, the current for the Co(NO_3_)_2_ solution increases, while the current for the Co(H**L**) solution remains more constant (Figure 8). The Δ*f* response is again negative in both cases. However, the Δ*f* corresponding to Co(H**L**) decreases rapidly in the first seconds and then stabilizes. In the case of Co(NO_3_)_2_, the Δ*f* decreases significantly faster and more continuously compared to Co(H**L**), indicating that significantly more material is deposited on the electrode surface.

### Faradaic Efficiency

The faradaic efficiency of water oxidation catalyzed by Co(H**L**) and Co–Pi toward dioxygen was investigated by detection of O_2_ in solution during chronoamperometry. For Co–Pi, a faradaic efficiency of 100% was found, which is in good agreement with the reported value (Figure S42).^68^ For homogeneous catalysts, it is more difficult to achieve such high Faradaic efficiencies due to partly oxidized species, for example, Co(III) compounds, diffusing away from the electrode before these can turnover. For Co(H**L**), a Faradaic efficiency of 83 ± 6% was found, which is a typical value for homogeneous Co-based catalysts (Figure S42).^92,94−97,102^ No visible changes in color, nor significant changes in the UV–vis spectrum were observed upon bulk electrolysis of a solution containing Co(H**L**) for 5 h at a relatively high potential of 1.49 V versus NHE (Figure S44).

### X-Ray Photoelectron Spectroscopy

The deposits formed on the Au EQCM electrode surface in solutions containing either Co(NO_3_)_2_ or Co(H**L**) were further investigated using XPS after the electrochemical CV experiment (Figure 8). These samples were compared regarding their Co, Au, N, P, and Na signals and directly compared to powder samples of their precursors [Co(H**L**)](OAc)_2_] and Co(NO_3_)_2_·6 H_2_O. Survey XP spectra indicate the presence of these elements in the samples (Figures S45–S48).

The powder reference of [Co(H**L**)(OAc)_2_] shows broad signals at BE(Co 2p_3/2_) = 780.8 (BE = binding energy) and 778.2 eV, which point to the presence of Co^II^ and Co^0^, respectively (Figure S50). The assignment of Co^II^ is confirmed by the satellite structure at BE(Co 2p_3/2_-sat.) = 786.4 eV with the typical energy difference of about 6 eV to the main Co 2p_3/2_ emission.^116^ The presence of Co^0^ is most likely due to X-ray beam damage of the powder sample, as no indication of Co^0^ was found by any of the other characterization techniques used. After catalysis, weak signals are found in the Co 2p region for Co(H**L**) on the Au electrode although the binding energy of the Co 2p_3/2_ emission at 780.8 eV corresponds with that of the [Co(H**L**)(OAc)_2_] powder reference. The XP spectra of the Co-Pi system correspond well with the literature, as the observed peaks at BE(Co 2p_3/2_) = 780.8 eV and BE(Co 2p_1/2_) = 796.8 eV are in good agreement with emissions at 780.7 and 795.7 eV in the reported XP spectra.^68^ The XP spectrum of power reference Co(NO_3_)_2_·6H_2_O shows a peak at BE(Co 2p_3/2_) = 782.2 eV, although this is 1 eV higher than reported in the literature.^117^ A comparison of the relative Co 2p and Au 4f signal intensities of the postcatalysis Co(H**L**) and Co-Pi electrodes indicates that the Co-Pi layer is significantly thicker than the Co(H**L**) layer, which is in good agreement with the larger Δ*f* observed in the EQCM studies (see Figure 6 for CV in combination with EQCM and Figure S51 for the Au 4f XP spectrum).

**Figure 9:**
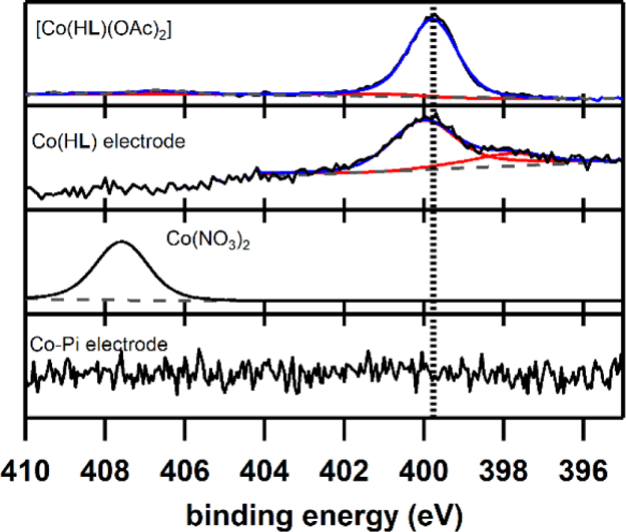
XP spectra N 1s region. The XP spectra (black), components (red), sum of components (blue), and background (dashed grey) are shown for each sample. From top to bottom: (1) powder reference [Co(H**L**)(OAc)_2_] (2) Co(H**L**) on an Au electrode surface after 50 cycles between 1.1 and 1.6 V versus NHE in a 100 mM pH 7 phosphate buffer with a scan rate of 100 mV/s (3) powder reference Co(NO_3_)_2_·6 H_2_O (the component overlaps with the XP spectra) (4) Co–Pi on an Au electrode surface after 50 cycles between 1.1 and 1.6 V versus NHE in a 100 mM pH 7 phosphate buffer with a scan rate of 100 mV/s.

To further elucidate the character of the deposit formed on the electrode surface from the Co(H**L**)-containing solution, XPS analysis of the N 1s, P 2p, and Na 1s regions was required. Clear peaks were found in the N 1s spectra for the powder sample [Co(H**L**)(OAc)_2_] and the Co(H**L**) postcatalysis sample, displaying emissions at BE(N 1s) = 399.8 and 400.0 eV, respectively (Figure 9). The occurrence of N 1s signals in this BE region indicates the presence of C–N species, such as pyridines of the H**L** ligand in the powder reference as well on the electrode surface.^118^ The presence of a free H**L** ligand on the electrode surface is excluded as the N 1s signal of the postcatalysis sample of Co(H**L**) and the H**L** ligand do not match (Figure S52). The background-subtracted peak areas were found to give an atomic Co to N ratio of around 1:6 (14 atom % Co; 86 atom % N) for both the powder reference and for the Co(H**L**) electrode, pointing to a 1:1 ratio of Co to H**L** in the deposit of Co(H**L**) (Table S2). For the Co(NO_3_)_2_ powder sample, an emission at BE(N 1s) = 407.6 eV was found, which is indicative of the N in the nitrate ion.^119,120^ As expected, *N*-based signals were not observed for the electrode surface containing Co-Pi. The Co-Pi system has been reported to show a signal at a BE(P 2p_3/2_) = 132.9 eV, which we also found for our Co-Pi sample (Figure S53).^68^ For the electrode with Co(H**L**) after catalysis, we did not find this signal on the electrode, indicating that the species on the surface does not contain detectable amounts of phosphate and thus shows no evidence for transformation to a Co-Pi active phase. A signal for Na 1s at 1071.3 eV was only observed on the electrode with a Co-Pi deposit, showing that sodium ions are present in the Co–Pi structure (Figure S54). Although BE in the Co 2p spectrum is rather inconclusive, other spectral regions, such as N 1s, P 2p, and Na 1s, can be helpful to arrive at a clearer picture.^84^ Based on the absence of any P and Na signals in postcatalysis XPS spectra of Co(H**L**), it can be concluded that no Co-Pi layer was formed. This is in contrast to the XP spectra reported by the Anderson group in the case of [Co(2,6-(bis(bis-2-*N*-methylimidazolyl)hydroxymethyl)pyridine)]^2+^ for which P and Na signals were observed in deposits obtained from this molecular precursor.^80^

### Homogeneity Considerations

Our combined electrochemical measurements and postcatalysis XPS studies show clear signs of a deposit formation on the electrode in the case of Co(H**L**), yet these deposits contain features that are significantly different than that of Co-Pi or other forms of CoO_*x*_ that have been reported previously.^68,70,80,82,84^ Based upon the dipping test and EQCM results, it can be concluded that throughout cycling over the Co^II/III^ redox waves, no significant deposition occurs. With a slope of −59 mV/pH in the Pourbaix diagram, oxidation of Co(H**L**) was found to proceed via PCET, presumably via oxidation of [Co^II^(H**L**)(H_2_O)_2_]^2+^ to [Co^III^(**L**^–^)(H_2_O)_2_]^2+^ or [Co^III^(H**L**)(H_2_O)(OH)]^2+^.

In the next oxidation event, [Co^III^(H**L**)(H_2_O)(OH)]^2+^ is irreversibly oxidized via another PCET step. This oxidation matches well with that of the analogous zinc complex, and, therefore, is most probably linked with oxidation of the redox-active ligand, forming [Co^III^(L^•^)(H_2_O)(OH)]^2+^ rather than a high-valent species such as [Co^IV^(H**L**)(H_2_O)(=O)]^2+^. The utilization of a redox-active ligand allows for circumvention of a Co^IV^ intermediate, which might be linked to CoO_*x*_ formation.^80,84^

The postcatalysis dipping test and EQCM experiments are indicative of mass accumulation on the electrode surface at higher potentials. The utilization of postcatalysis studies has solved the homogeneity debate for Co-based WOCs in the past.^80,82,84,91−94^ In the case of Co(H**L**), the unchanged atomic Co/N ratio in XPS and the presence of the Co^II/III^ redox couple in postcatalysis cyclic voltammetry indicate the intact compound, possibly as part of some Co(H**L**) aggregate or cluster, is present on the electrode surface. The catalytic wave of a molecular catalyst typically reaches a plateau as eventually, the rate-determining step shifts from an electron-transfer step to a chemical step.^121^ This is not observed in the case of the Co(H**L**) deposit, for which a continuous catalytic wave is observed that is more typical of heterogeneous systems. This may be the result of a combination of a relatively poor electron conductivity through the multiple layers of Co(H**L**) and an effect of the Co sites being chemically inequivalent due to their surrounding within the amorphous layer rather than the shape of the wave being indicative of a heterogeneous catalyst.

As is common in the field of catalysis, it remains difficult to fully exclude that undetectable amounts of cobalt oxide nanoparticles are present in the deposit and that they are responsible for some of the catalytic activity and especially that such species might accumulate over time under the oxidative conditions employed during prolonged water oxidation catalysis.^122^ Nevertheless, our data shows that most likely electrocatalytic water oxidation activity should at least initially be ascribed to a deposition of “molecular” species resembling Co(H**L**) rather than Co–Pi.

## Conclusions

We found that during the water oxidation reaction, deposits of Co(H**L**) are formed on the electrode surface. The elemental composition of these deposits does not match that of CoO_*x*_ or more specifically Co–Pi but instead fits better to a deposit of a molecular species still containing the organic ligand. This is in contrast to many other Co-systems investigated previously and, to the best of our knowledge, the first time that a deposition of a Co-based WOC does not largely contain CoO_*x*_. We believe that this is an important observation that highlights that homogeneity and decomposition of the homogeneous Co-based catalyst are not necessarily related.

## Supporting Information Available

Experimental information, single-crystal X-ray crystallography, ^1^H-NMR, color transition, UV-Vis, electrochemical experiments, XPS analysis of the electrode surface, NMR spectra, and mass spectrometry (PDF)
Crystallographic data (CIF)

## Supplementary Material

cs2c01299_si_001.pdf

cs2c01299_si_002.cif
