# Supplementary material for: On the Homogeneity of a Cobalt-Based Water Oxidation Catalyst
Source: ACS Catal. 2022 Apr 4;12(8):4597–607. doi: 10.1021/acscatal.2c01299 (PMC9016703; doi:10.1021/acscatal.2c01299)
Supplement: Supplementary file 1 — cs2c01299_si_001.pdf [file cs2c01299_si_001.pdf]

Supporting information

On the homogeneity of a cobalt-based water oxidation catalyst

Daan den Boer,[a] Quentin Siberie,[a] Maxime A. Siegler,[b] Thimo H. Ferber,[c] Dominik C. Moritz,[c] Jan P. Hofmann,[c] and Dennis G. H. Hetterscheid,[a]*

[a] Leiden Institute of Chemistry, Leiden University, Einsteinweg 55, 2300 RA, Leiden, The Netherlands

[b] Department of Chemistry, Johns Hopkins University, 3400 North Charles St., Baltimore, Maryland 21218, United States

[c] Surface Science Laboratory, Department of Materials and Earth Sciences, Technical University of Darmstadt, Otto-Berndt-Strasse 3, 64287 Darmstadt, Germany

**Corresponding author:* d.g.h.hetterscheid@chem.leidenuniv.nl

Contents

I. Experimental 4

General information 4

Electrochemistry 4

Synthetic protocols 8

II. Single crystal X-ray crystallography 10

III. ^{1}H-NMR 14

IV. Color transition 15

V. UV-Vis 16

VI. Electrochemical experiments 19

Cyclic and differential pulse voltammograms 19

Dipping test and post catalysis study 23

Electrochemical quartz crystal microbalance 32

^{31}P NMR 36

Co^{2+} trapping with EDTA 39

Cyclic voltammograms after chronoamperometry 41

Oxygen detection and Faradaic efficiency 43

VII. XPS analysis of the electrode surface 48

VIII. NMR spectra 55

IX. Mass spectrometry 58

Abbreviations

aq	aqueous
CE	Counter Electrode
CV	Cyclic Voltammogram
EQCM	Electrochemical Quartz Crystal Microbalance
equiv	equivalent
ESI-MS	ElectronSpray Ionization Mass Spectrometry
GC	Glassy Carbon
LC-MS	Liquid Chromatography Mass Spectrometry
NHE	Normal Hydrogen Electrode
NMR	Nuclear Magnetic Resonance
RE	Reference Electrode
RHE	Reversible Hydrogen Electrode
S-BINAP	(S)-(-)-2,2'-Bis(diphenylphosphino)-1,1'-binaphthyl
UV-vis	Ultraviolet-visible
WE	Working Electrode
XPS	X-ray photonelectron spectroscopy

Abbreviations in NMR spectroscopy

APT	Attached Proton Test
COSY	Correlated SpectroscopY
C_q	Quaternary carbon
d	Doublet
dt	Doublet of Triplets
HSQC	Heteronuclear Single Quantum Coherence
J	coupling constant
m	Multiplet
t	Triplet
td	Triplet of Doublets
δ	chemical shift

I. Experimental

General information

Chemicals were purchased from Acros Organics (*tert*-butylamine), Sigma Aldrich (H_2O_2 (35% aq.), 6-Bromo-2,2'-bipyridine, $PhCF_3$, KO*t*Bu, Ts_2O, $Zn(OTf)_2$ (98%), $Pd(dba)_2$), Alfa Aesar (2,2'-bipyridine, $MgSO_4$, trifluoracetic acid, cobalt(II) acetate tetra hydrate (98%)), Brunswig Chemie ((*S*)-BINAP), Carl Roth GMBH (NaOH) and VWR (MeOH, CH_2Cl_2, Et_2O). (S)-BINAP and KO*t*Bu were stored under argon in a glovebox. All other compounds were used as is without any further purification. H**L** was synthesized following literature procedures.[1-4]

NMR spectra (^{1}H, ^{13}C APT, COSY and HSQC) were recorded on a Bruker AV 300 or 400 MHz NMR Spectrometer. Chemical shifts (δ) are reported in parts per million (ppm) using the residual solvent as internal standard. Coupling constants (*J*) are reported in Hertz (Hz). UV-vis spectra were recorded on a Varian Cary 50 Scan spectrophotometer in a 1 cm path length quartz cuvette. Mass spectra were obtained by LC-MS (Thermo Finnigan AQA ESI-MS). Elemental analyses were performed by Mikroanalytisches Laboratorium Kolbe in Oberhausen, Germany. Magnetic susceptibility was measured with a Sherwood Scientific MK 1 Magnetic susceptibility balance.

Electrochemistry

Chemicals for electrochemistry

All chemicals for electrochemistry were commercial purchased and used without further purification. The following chemicals were used, including purity and supplier; Cobalt nitrate hexahydrate (99.999%, Sigma Aldrich), sodium dihydrogen phosphate (99.99%, Suprapur®, Merck), disodium hydrogen phosphate (99.999%, Fluka), sodium hydroxide monohydrate 99.99%, Suprapur®, Merck), Lead (II) nitrate (99.999% Sigma Aldrich), phosphoric acid (85% aq. Suprapur®), H_2SO_4 (98% aq., Suprapur®), $HClO_4$ (70% aq., Suprapur®), NBu_4PF_6 (99.0%, Sigma Aldrich), ferrocene (98% Fluka) and MeCN (Biosolve, HPLC grade).

Equipment electrochemistry

All electrochemical experiments (with exception of EQCM experiments) were performed in custom made single-compartment glass cells in a three-electrode setup. All electrochemical

cells were routinely cleaned by submerging the electrochemical glassware in a $KMnO_4$ solution (1 g/L $KMnO_4$ in 0.5 M H_2SO_4). Afterwards manganese byproducts were removed by addition of hydrogen peroxide (35% aq.). Milli-Q ultrapure (>18.2 MΩ cm resistivity) water was used in all experiments to clean glassware and to prepare aqueous solutions. Prior to each experiment, the glassware was cleaned by boiling at least once in Milli-Q water.

Autolab PGSTAT204 or PGSTAT128N potentiostats in combination with NOVA 2.0 software were used to perform electrochemical measurements. The pH of the electrolyte was measured with a Radiometer PHM220, calibrated following IUPAC buffers. All electrolytes had an ionic strength of around 0.3 M.

Electrodes and cleaning of the electrode surface

Glassy carbon (GC) electrodes encapsuled in polyether ether ketone (PEEK) with a 0.07 cm^2 geometrical surface were used as working electrode (Autolab). Prior to the experiment the GC electrode surface was mechanically polished with a Labopol-20 polishing machine. First, the electrode was polished with 1.0 micron diamond suspension on a Dur type polishing cloth for 2 minutes (Struers). The electrode was rinsed with 2-propanol to remove the organic substances from the slurry and subsequently rinsed with Milli-Q water. Next, the electrode was polished with a 0.4 micron silica suspension on a Dur type polishing cloth for 2 minutes and the electrode was rinsed with Milli-Q water afterwards. Finally, the electrode was sonicated in Milli-Q water for 10 minutes. In some cases, where deposits formed on the electrode, the electrode was polished with P2500 and P5000 sandpaper before mechanical polish.

A gold (99.9%, MaTeck) wire with a large surface was used as a counter electrode (CE) in all experiments. Prior to the experiment, the electrode was rinsed with Milli-Q water and the electrode was flame annealed. The reference electrode was a reversible hydrogen electrode (RHE). The RHE electrode consisted of a platinum mesh (MaTeck) in H_2-saturated (Linde, H_2 5.0) electrolyte at the same pH as the electrolyte solution inside the cell. The cell and the RHE electrode were connected via a Luggin capillary. The reference potentials were converted and reported vs. Normal Hydrogen Electrode (NHE).

Prior to measurements, the electrolyte solution was saturated with argon gas (Linde, Ar 5.0), by bubbling through the solution for at least 20 minutes. During the measurements, the cell was continuously kept under argon atmosphere to prevent air from entering the electrochemical cell. For the experiments in MeCN, the argon flow through the electrochemical cell was saturated with MeCN first by bubbling gas argon through the solvent in order to prevent evaporation of the electrolyte solution.

Electrochemical Quartz Crystal microbalance (EQCM)

EQCM experiments were performed on an Au electrode (0.35 cm^2 Autolab), that consisted of a 200 nm gold layer on a 6 MHz quartz crystal. The custom made electrochemical cell consisted of PEEK material and had a volume of 5 mL.

Chronoamperometry

Chronoamperometry measurements were performed using three electrochemical cells. First, the GC electrode was anodized in an electrochemical cell containing 100 mM pH 7 phosphate buffer. The potential was kept at 2.1 V vs. RHE for 25 minutes to fully anodize the electrode surface. Subsequently a blank chronoamperometry measurement was performed in another electrochemical cell containing 100 mM pH 7 phosphate buffer. The potential was kept at 1.66 V vs. RHE for 10 minutes to obtain a stable background current. The electrode was then placed in an electrochemical cell containing the cobalt compound of interest. The potential was kept at 1.66 V vs. RHE until the desired amount of charge was passed.

Faraday Efficiency

Bulk electrolysis was performed in a custom-made electrochemical cell in which the working electrode was separated from the counter electrode by an SELEMION™ AMVN anion-exchange membrane (AGC Engineering Co., Ltd). Each side of the cell contained 8.0 mL of electrolyte. The anode compartment consisted of a 3.1 cm^2 custom-made GC working electrode from a GC-plate (Alfa Aeser), a low profile Ag/AgCl electrode (Pine research) and a solution of the compound of interest (8.0 mL). The cathode compartment contained Au wires (MaTeck) and the blank solution (8.0 mL). Oxygen was detected in solution by a NeoFox oxygen probe (Oceaninsight) at the anode side. The solution was stirred by a magnetic stirring bar at 700-1000 rounds per minute. The electrodes were cleaned as described above, except

for the GC working electrode, which was manually polished for a few seconds on sandpaper P5000, followed by polishing on a microcloth with 1.0, 0.3 and 0.05 micron sizes of alumina suspensions (Buehler), respectively. After polishing the GC electrode, the excess alumina was removed by sonication of the electrode in Milli-Q water for at least 10 minutes.

X-ray photoelectron spectroscopy (XPS)

XPS data were obtained on a Physical Electronics PHI Versaprobe 5000 spectrometer in fixed analyzer transmission mode using monochromatic Al Kα radiation ($h\nu$ = 1486.6 eV, spot diameter 200 µm and a power of 50 W) at an analyzer angle of 45° with a pass energy of 23.50 eV (step size of 0.1 eV) for region scans and survey scans over a range of 1350-0 eV with a step size of 1.0 eV and a pass energy of 187.85 eV. The samples have been mounted and measured on sticky carbon tape and due to their good conductivity, no charge neutralization has been applied. The gold electrodes have been electronically connected to the spectrometer using silver glue to connect the edge of the gold with the sample holder. XP-spectra of sample Co-Pi were obtained on a Specs PHOIBOS 150 spectrometer at a perpendicular analyzer angle with monochromatic Al Kα radiation (Focus 500 with XR50M (SPECS) $h\nu$ = 1486.74 eV, spot diameter of 1000 µm with a power of 300 W). The survey and region spectra were measured in fixed analyzer transmission mode with a pass energy of 20 eV (step size of 1 eV) and 10 eV (step size of 0.05 eV), respectively. Binding energy calibration was performed by setting the C1s signal of sp^3 carbon to 284.8 eV. Due to the insufficient electric conductivity of the pure ligand powder, a neutralizer equipped with a low-voltage electron gun (BaO) was used to compensate the sample charging. Elemental compositions were determined using Scofield relative sensitivity factors available in CasaXPS 2.3.23PR1.0. Linear backgrounds have been applied. Component fits were done using Lorentzian asymmetric line shapes LA(1.53,243), based on a convolution of Lorentzian and Gaussian line shapes.

Synthetic protocols

[Co(HL)(OAc)$_2$]

H**L** (100 mg, 0.31 mmol, 1 equiv.) was dissolved in 6 mL MeOH. A purple solution of cobalt (II) acetate tetrahydrate (73 mg, 0.29 mmol, 0.95 equiv.) in MeOH (6 mL) was added to the H**L** solution, changing the color of the solution from dark yellow to orange. The resulting mixture was stirred for 5 hours at room temperature, followed by removal of the solvent *in vacuo*. The mixture was redissolved in a minimal amount of MeOH and crystals were grown by vapor diffusion with MeOH/Et_2O at 4°C. The obtained crystals were isolated by filtration and were washed thoroughly with Et_2O. After drying the crystals at 40°C *in vacuo* the target compound, [Co(H**L**)(OAc)$_2$] was obtained (74 mg, 0.15 mmol, 52%). Single crystals suitable for X-ray diffraction were obtained by vapor diffusion in MeOH/Et_2O at –30°C.

Elemental analysis:
Calculated (%) for $C_{24}H_{21}CoN_5O_4$ (502.40 g/mol): C: 57.38, H: 4.21, N: 13.94 found: C: 57.12, H: 4.19, N: 13.87

MS (ESI) m/z: calc. for [Co(H**L**)]$^{2+}$: 192.0, found 192.2;
calc. for [Co(**L**)]$^{+}$: 383.1, found 383.1;

UV-VIS extinction coefficient ϵ (L·mol^{-1}·cm^{-1}) :
ϵ (232 nm): 2.02 · 10^4
ϵ (254 nm): 1.17 · 10^4
ϵ (280 nm): 1.70 · 10^4
ϵ (344 nm): 1.29 · 10^4

Magnetic moment: 4.29 μB

[Zn(HL)](OTf)$_2$

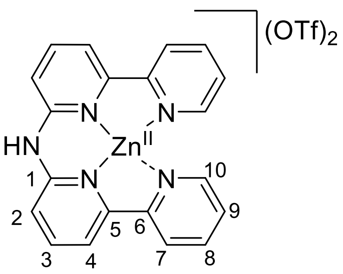

H**L** (0.051 g, 0.16 mmol, 1 equiv.) was dissolved in 10 mL MeOH. A solution of Zn(OTf)$_2$ (0.054 g, 0.15 mmol, 0.95 equiv.) in 6 mL methanol was added to the H**L** solution. The resultant mixture was stirred overnight at room temperature followed by removal of the solvent *in vacuo*. The mixture was redissolved in a minimal amount of MeOH and crystals were grown by vapor diffusion with MeOH/Et$_2$O at 4°C. The obtained crystals were isolated by filtration and were washed thoroughly with Et$_2$O. After drying the crystals at 40°C *in vacuo* the target compound, [Zn(H**L**)](OTf)$_2$ was obtained (0.080 g, 0.12 mmol, 74%).

^{1}H NMR (400 MHz, methanol-*d*$_4$) δ: 9.24 (d, *J* = 5.0 Hz, 2H, H-10), 8.77 (d, *J* = 8.2 Hz, 2H, H-7), 8.43 (td, *J* = 8.1, 1.5 Hz, 2H, H-8), 8.33 (d, *J* = 7.6 Hz, 2H, H-4), 8.25 (t, *J* = 8.0 Hz, 2H, H-3), 8.02 – 7.96 (m, 2H, H-9), 7.50 (d, *J* = 8.3 Hz, 2H, H-2).

^{13}C NMR (101 MHz, methanol-*d*$_4$) δ: 155.08 (C_q), 150.86 (C_q), 150.21 (C10), 147.52 (C_q), 143.57 (C-3), 143.09 (C-8), 128.44 (C-9), 124.70 (C-7), 118.42(C-4), 117.30 (C-2).

MS (ESI) m/z: calc. for [Zn(H**L**)]$^{2+}$: 194.5, found 194.8;
calc. for [Zn(H**L**)(MeOH)]$^{2+}$: 210.5, found 210.7;
calc. for [Zn(**L**)]$^{+}$: 388.1, found 388.1;
calc. for [Zn(H**L**)(OTf)]$^{+}$: 538.0, found 538.0.

Elemental analysis:

Calculated (%) for $C_{22}H_{15}ZnF_6N_5O_6S_2 \cdot 0.25\ C_4H_{10}O$ (707.14 g/mol): C: 39.08, H: 2.42, N: 9.91.
Found: C: 39.11, H: 2.36, N: 9.86.

II. Single crystal X-ray crystallography

All reflection intensities were measured at 110(2) K using a SuperNova diffractometer (equipped with Atlas detector) with Mo *K*α radiation (λ = 0.71073 Å) under the program CrysAlisPro. The same program was used to refine the cell dimensions and for data reduction. The structure was solved with the program SHELXS-2018/3 and was refined on F^2 with SHELXL-2018/3. Numerical absorption correction based on gaussian integration over a multifaceted crystal model was applied using CrysAlisPro. The temperature of the data collection was controlled using the system Cryojet (manufactured by Oxford Instruments). The H atoms were placed at calculated positions using the instructions AFIX 43 or AFIX 137 with isotropic displacement parameters having values 1.2 or 1.5 U_{eq} of the attached C or N atoms. The H atoms attached to O1S and O1S' were found from difference Fourier maps, and their coordinates were restrained pseudo freely using the DFIX instruction in order to keep the O–H bond distances within an acceptable range. The structure was initially solved and refined in the space group *P*1, and the structure was also found to be disordered.

The Co complex is found to be disordered as it is located at one site of inversion symmetry. The occupancy factor was constrained to be 0.5. The asymmetric unit also contains one disordered MeOH lattice molecule, and the occupancy factor of the major component of the disorder refines to 0.748(5)

Computer programs: *CrysAlis PRO* 1.171.39.29c (Rigaku OD, 2017), *SHELXS2018*/3 (Sheldrick, 2018), *SHELXL2018*/3 (Sheldrick, 2018), *SHELXTL* v6.10 (Sheldrick, 2008).[5]

Table S1. Experimental details X-ray structure analysis

	**[Co(HL)(OAc)$_2$]**
Crystal data	
Chemical formula	$(C_{24}H_{21}CoN_5O_4)\cdot 2(CH_4O)$
M_r	566.47
Crystal system, space group	Triclinic, *P*-1
Temperature (K)	110
a, *b*, *c* (Å)	7.4012 (2), 8.5138 (3), 11.0680 (4)
α, β, γ (°)	90.500 (3), 95.188 (3), 111.907 (3)
V (Å^3)	643.73 (4)
Z	1
Radiation type	Mo *K*α
μ (mm^{-1})	0.72
Crystal size (mm)	0.37 × 0.22 × 0.06
Data collection	
Diffractometer	SuperNova, Dual, Cu at zero, Atlas
Absorption correction	Gaussian<br>*CrysAlis PRO* 1.171.39.29c (Rigaku Oxford Diffraction, 2017) Numerical absorption correction based on gaussian integration over a multifaceted crystal model Empirical absorption correction using spherical harmonics, implemented in SCALE3 ABSPACK scaling algorithm.
T_{min}, T_{max}	0.534, 1.000
No. of measured, independent and observed [$I > 2\sigma(I)$] reflections	11899, 2959, 2726
R_{int}	0.024
$(\sin \theta/\lambda)_{max}$ (Å^{-1})	0.650
Refinement	
$R[F^2 > 2\sigma(F^2)]$, $wR(F^2)$, S	0.036, 0.089, 1.12
No. of reflections	2959
No. of parameters	329
No. of restraints	263
H-atom treatment	H atoms treated by a mixture of independent and constrained refinement
$\Delta\rho_{max}$, $\Delta\rho_{min}$ (e Å^{-3})	0.49, -0.27

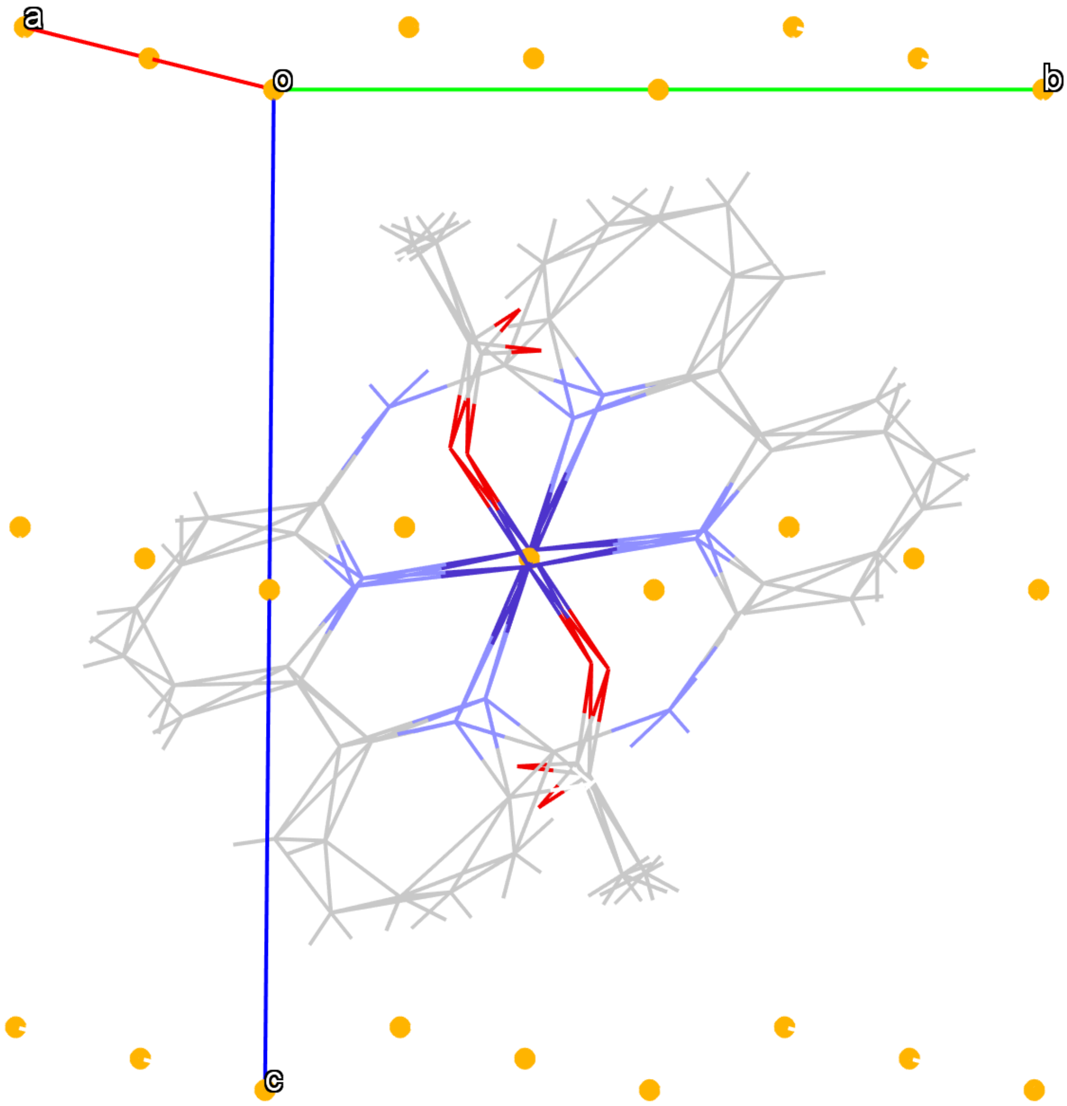

Figure S1. [Co(H**L**)(OAc)$_2$] in the unit cell (P-1 space group) showing inversion centers (orange dots).

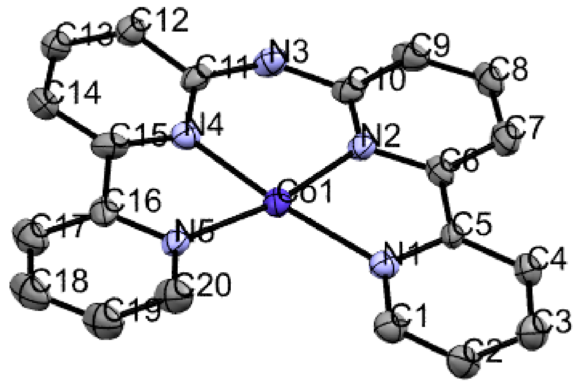

Figure S2. Displacement ellipsoid plot (50% probability level) of [Co(H**L**)(OAc)$_2$] at 110(2) K including the atom numbering. Hydrogen atoms and acetate ions were omitted for clarity.

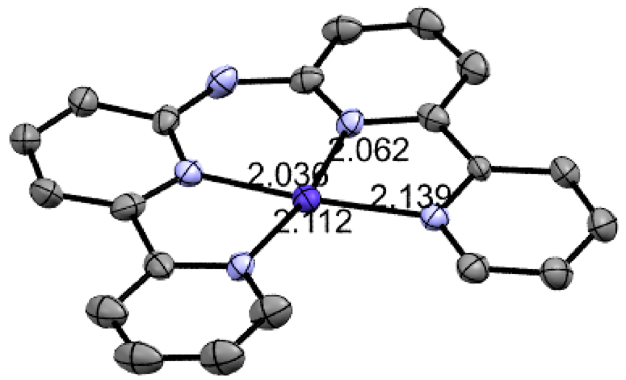

Figure S3. Displacement ellipsoid plot (50% probability level) of [Co(H**L**)(OAc)$_2$] at 110(2) K, including Co-N bond lengths. Hydrogen atoms and acetate ions were omitted for clarity.

III. ^{1}H-NMR

The ^{1}H-NMR spectrum of a 0.5 mM Co(H**L**) was recorded in D_2O at 300 MHz (Figure S4). Except for the residual solvent peak (4.79 ppm), only a singlet is found at 1.88 ppm, which corresponds to the CH_3 group of the acetate group.[6] Spiking the 0.5 mM Co(H**L**) solution with NaOAc results in an increase of the signal at 1.88 ppm, indicating the signal indeed corresponds to the free acetate ion in solution. This indicates the acetate groups are not coordinated in aqueous solution and are likely to be exchanged for water molecules.

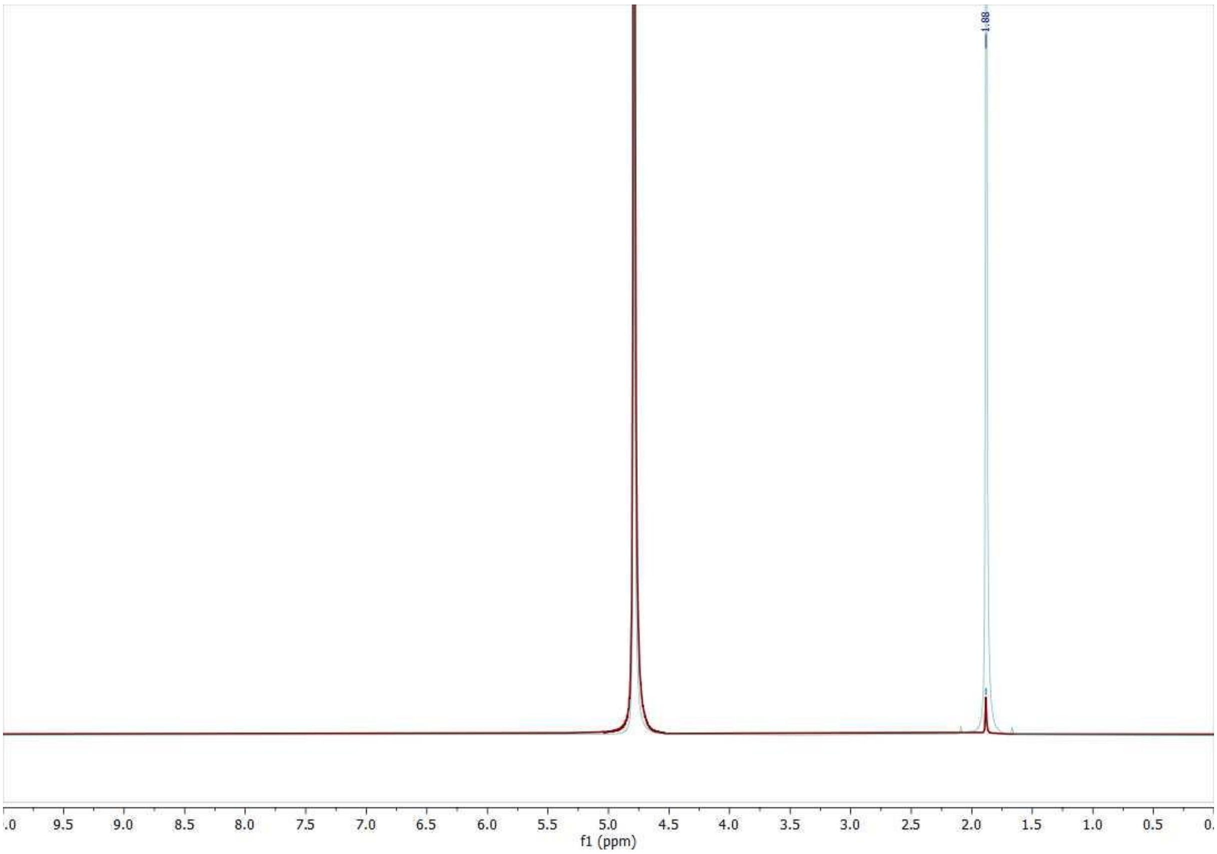

Figure S4. ^{1}H-NMR of 0.5 mM Co(H**L**) in D_2O (red) and 0.1 M NaOAc in D_2O (blue) recorded at 300 MHz (red).

IV. Color transition

A yellow 0.5 mM solution of $[Co(H\mathbf{L})(H_2O)_2]^{2+}$ in Milli-Q water was prepared in a glass tube. Upon submerging the solution in a dry ice/acetone bath (–78°C), the color changed from yellow to purple. Leaving the tube out of the dry ice, allowing it to heat to room temperature, the solution turned yellow again, indicating the process is reversible.

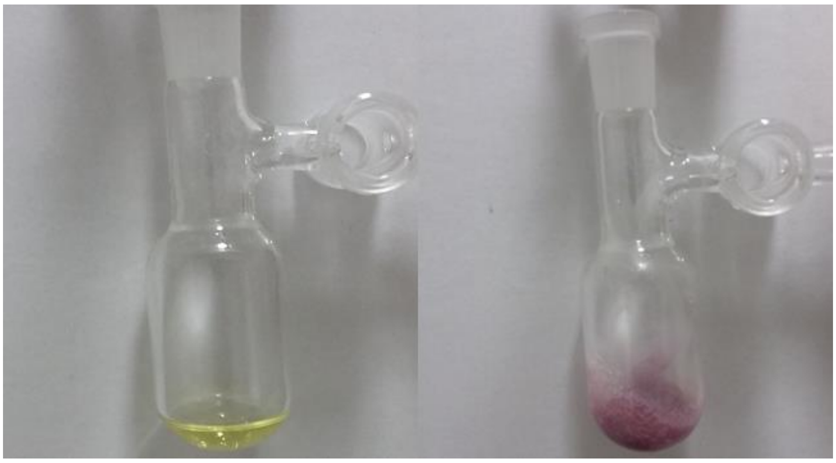

Figure S5. Solutions of Co(H**L**) in Milli-Q water at room temperature (left) and at –78 °C (right).

V. UV-Vis

The UV-vis spectrum of an aqueous solution of $[Co(H\mathbf{L})(H_2O)_2]^{2+}$ was recorded directly upon dissolving (Figure S4). Absorbance peaks were found at 232, 254, 281 and 334 nm. The solution was kept in a closed cuvet for 6 days. The spectrum after 6 days did not change compared to the spectrum recorded of the fresh solution, indication $[Co(H\mathbf{L})(H_2O)_2]^{2+}$ is stable in aqueous solution. UV-vis spectra of varying concentrations $[Co(H\mathbf{L})(H_2O)_2]^{2+}$, consisting of 5, 7.1, 8.3, 10, 12.5, 16.7, 25 and 50 µM were recorded (Figure S5). From the concentration versus absorbance plots, extinction coefficients were determined for the absorbance bands; $\varepsilon_{232\ nm} = 2.0 \cdot 10^4$, $\varepsilon_{254\ nm} = 1.2 \cdot 10^4$, $\varepsilon_{280\ nm} = 1.7 \cdot 10^4$, $\varepsilon_{344\ nm} = 1.3 \cdot 10^4$ L· mol^{-1} ·cm^{-1} (Figure S6). The pK_a of the ligand was determined by UV-vis monitored titration with NaOH. Upon addition of NaOH, the solution changed visually from yellow to orange and the absorbance bands shift to 337 and 418 nm (Figure S7). Monitoring the arising absorbance at 418 nm versus pH, results in a titration graph in which half of the wave corresponds to the pK_a of 10.2 (Figure S8).

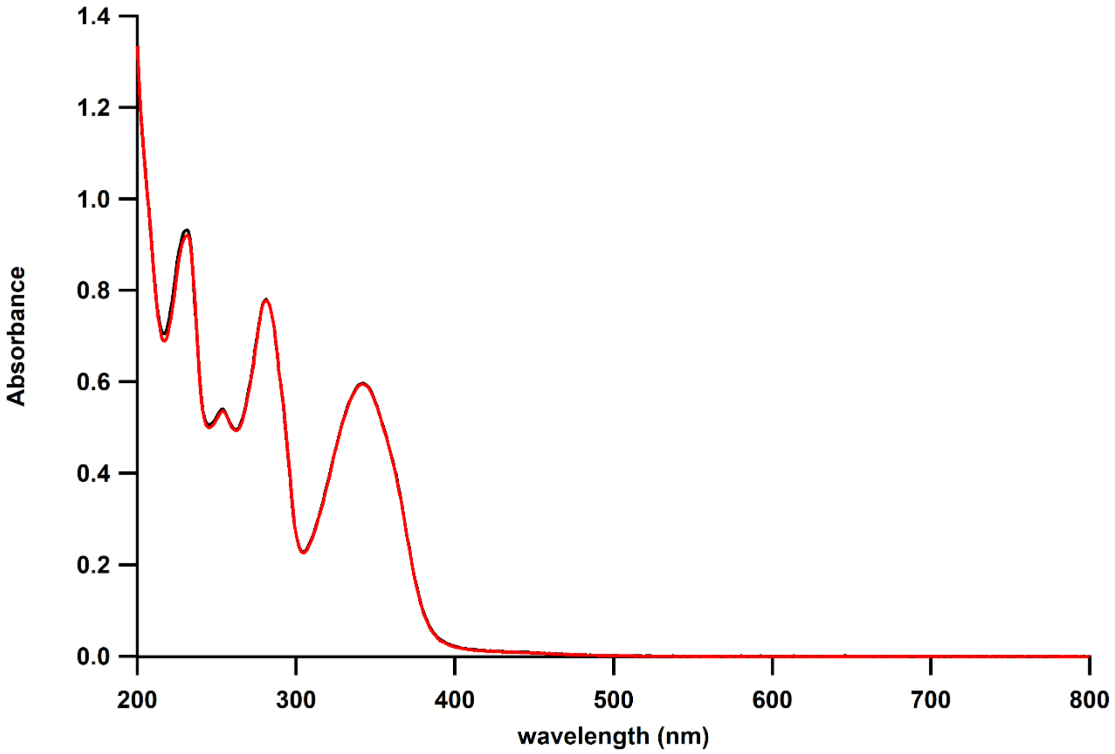

Figure S6. UV-vis spectra of Co(H**L**) in Milli-Q water directly after dissolving (black line) and after 6 days (red).

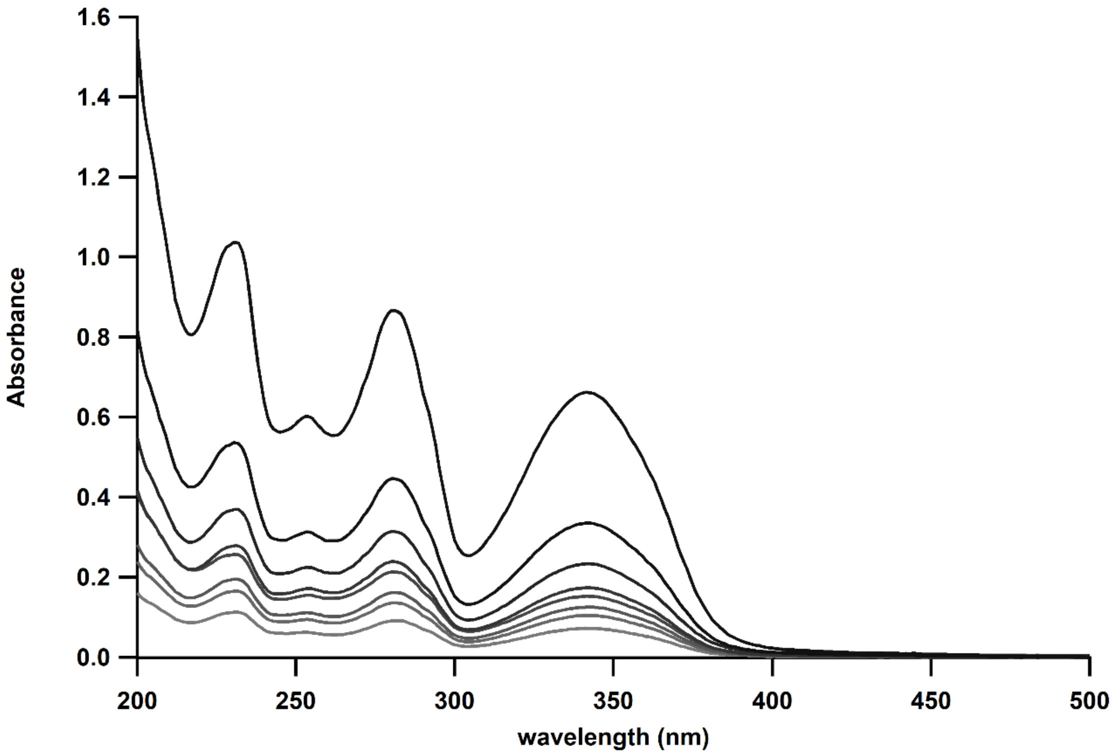

Figure S7. UV-vis spectra of Co(H**L**) in Milli-Q water at varying concentration between 5 and 50 µM.

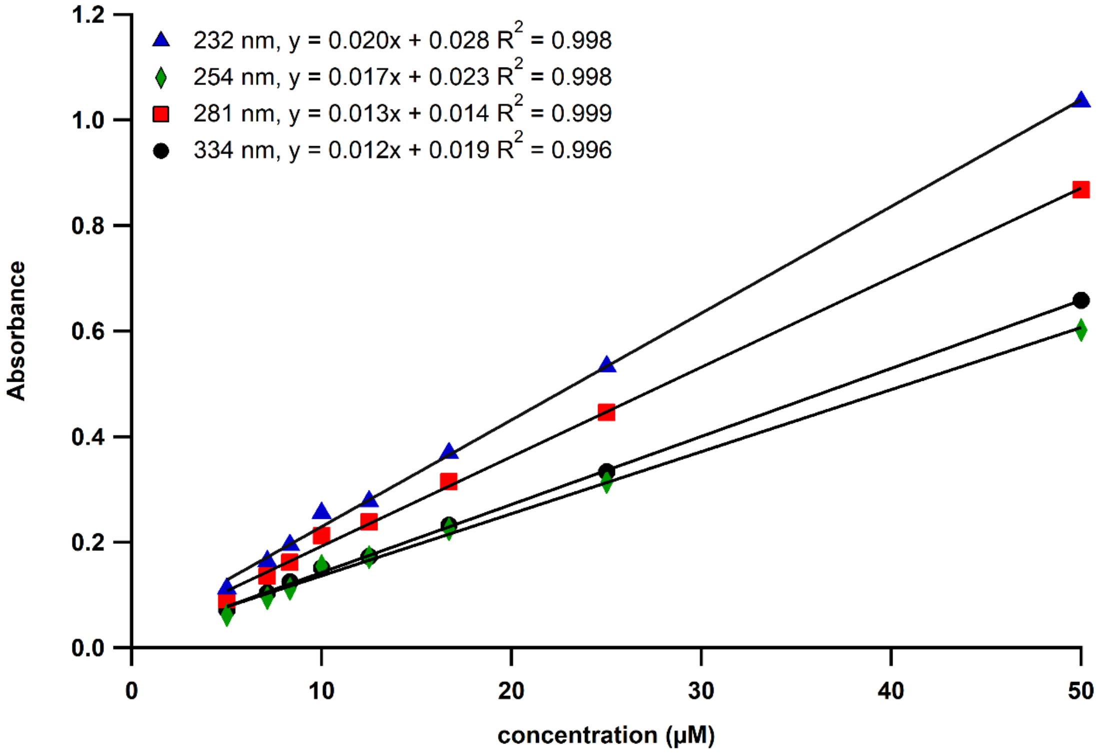

Figure S8. Absorbance dependence on the concentration of Co(H**L**) in Milli-Q water for absorbance bands at 232, 254, 281 and 334 nm.

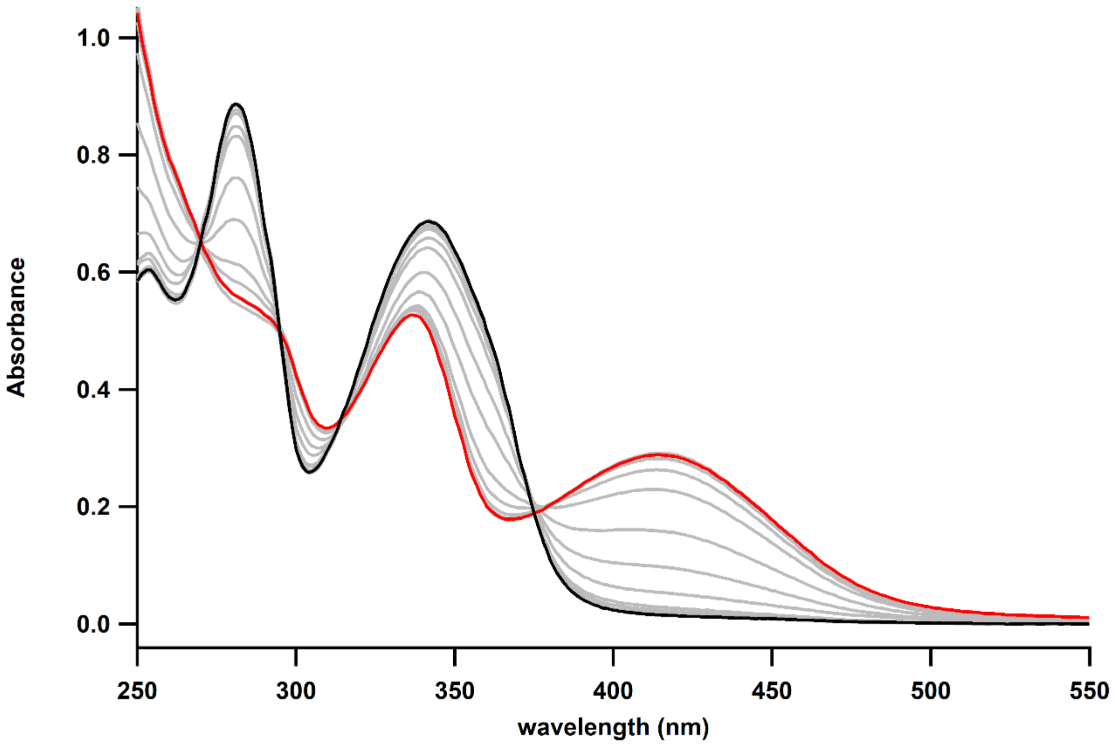

Figure S9. UV-vis spectra of Co(H**L**)/Co(**L**) in Milli-Q water during titration with NaOH between pH 6.2 (black) and pH 12.0 (red).

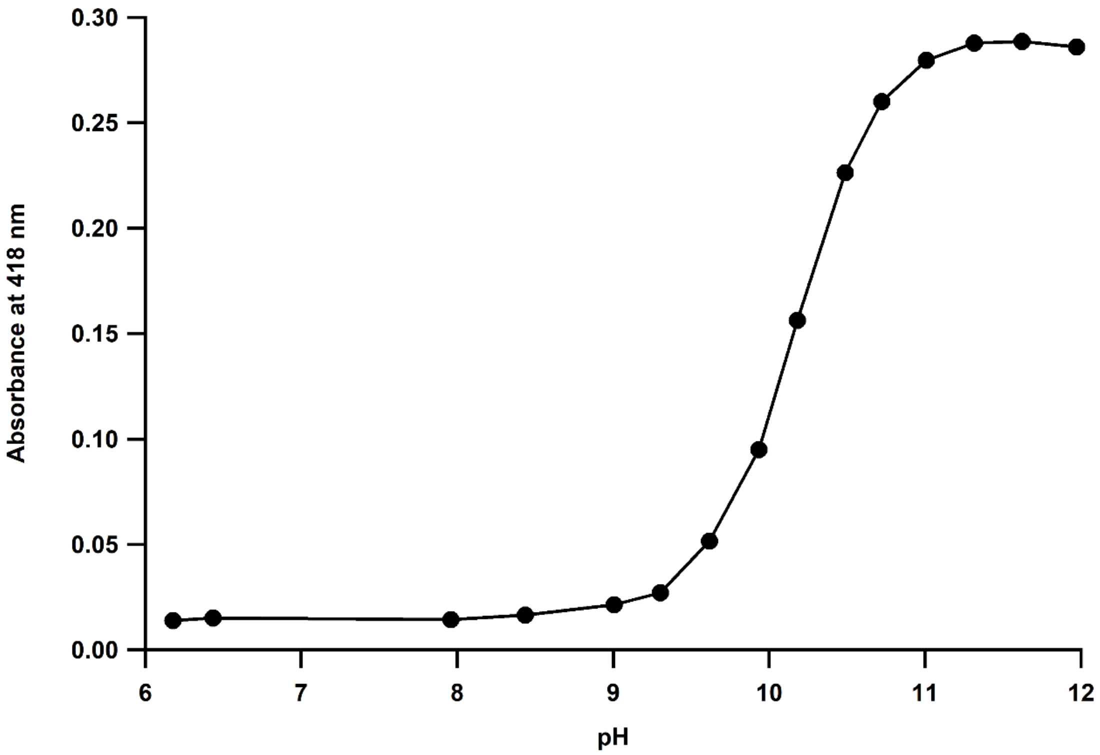

Figure S10. Absorbance at 418 nm of Co(**L**) in Milli-Q water as a function of pH. The equivalence point is calculated to be 10.2, corresponding to the pK_a of the N–H group of the ligand.

VI. Electrochemical experiments

Cyclic and differential pulse voltammograms

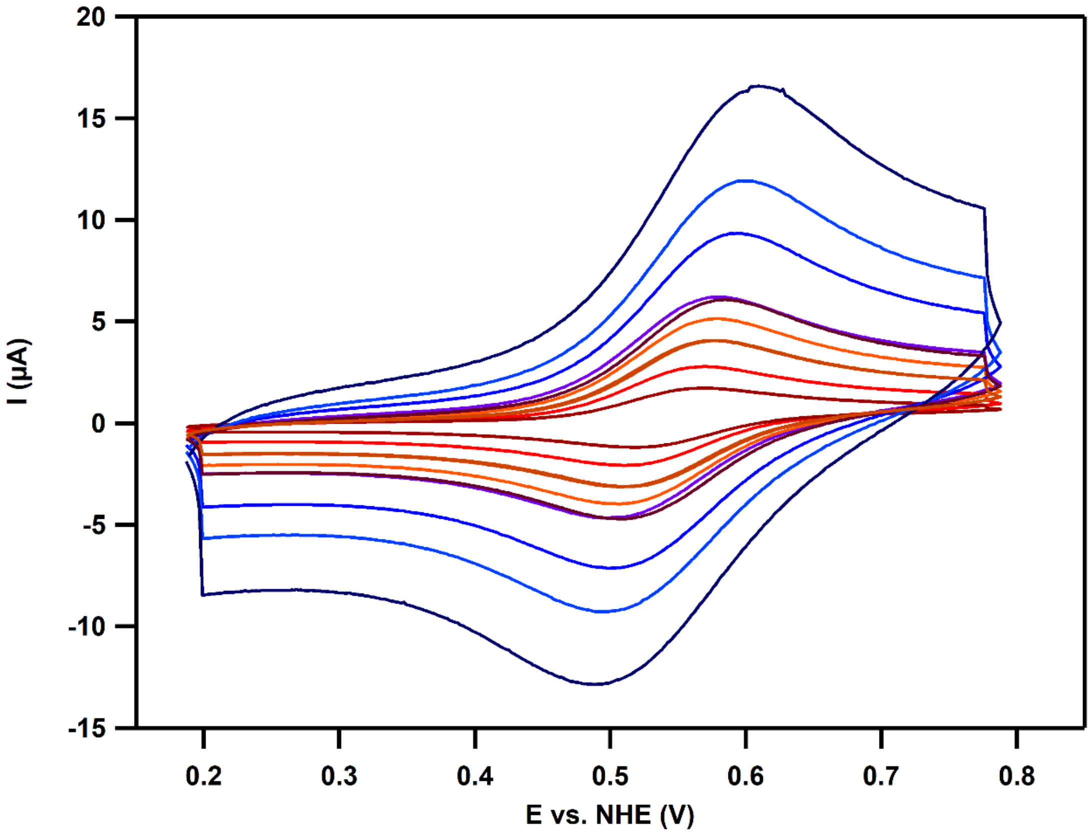

Figure S11. Cyclic Voltammograms of 0.5 mM Co(H**L**) in a 100 mM pH 7 phosphate buffer at varying scan rate (10 -100 mV/s). GC, Au and RHE were used as WE, CE and RE, respectively. Potentials were converted to NHE.

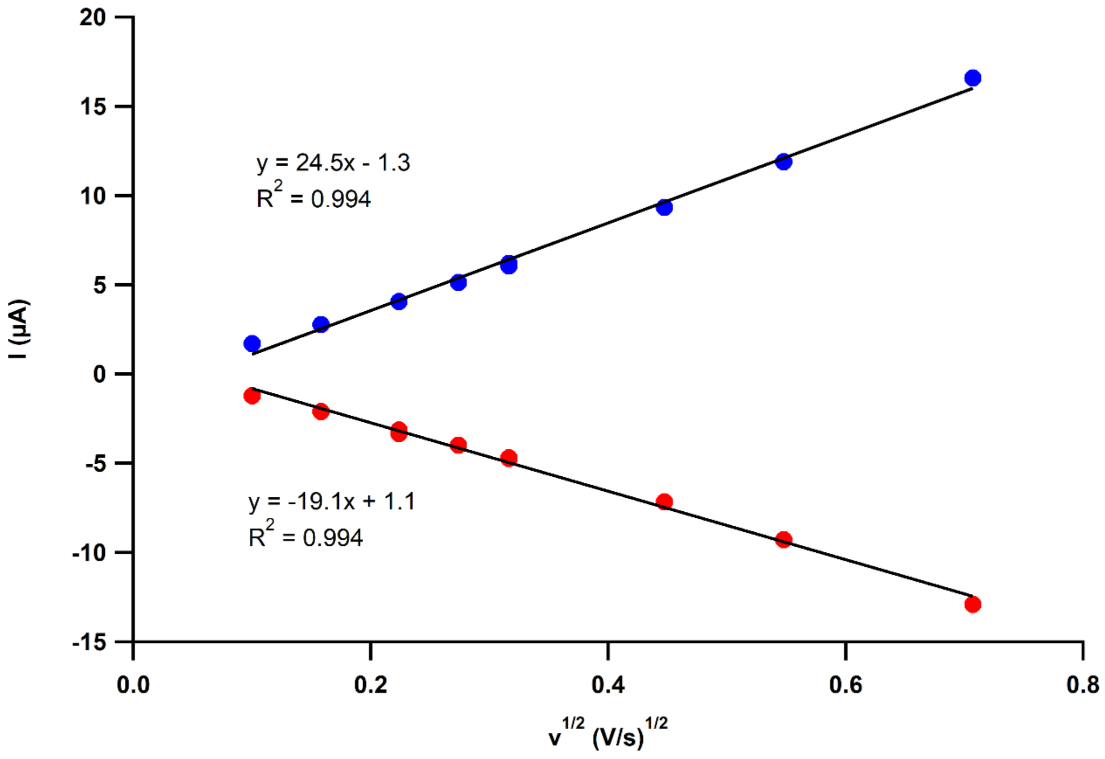

Figure S12. Linear dependence on the square root of the scan rate for the oxidative and reductive peak currents of the $Co^{II/III}$ redox couple of Co(H**L**).

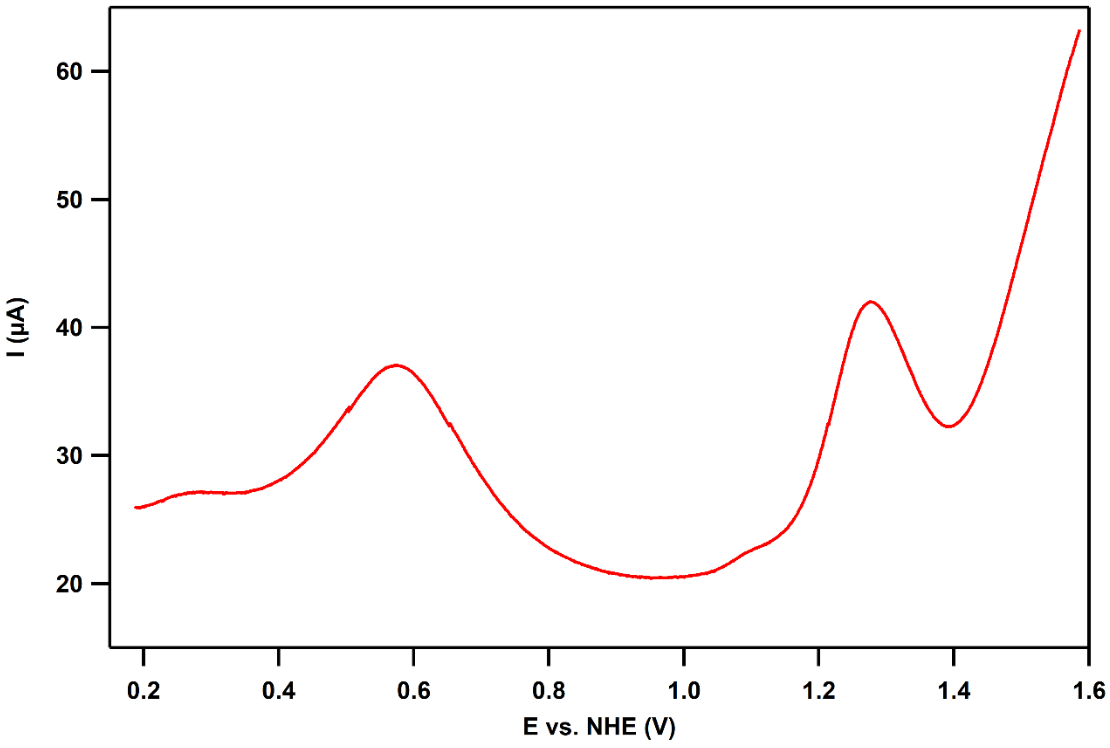

Figure S13. Differential pulse voltammogram of 0.5 mM Co(H**L**) in a 100 mM pH 7 phosphate buffer. GC, Au and RHE were used as WE, CE and RE, respectively. Potentials were converted to NHE.

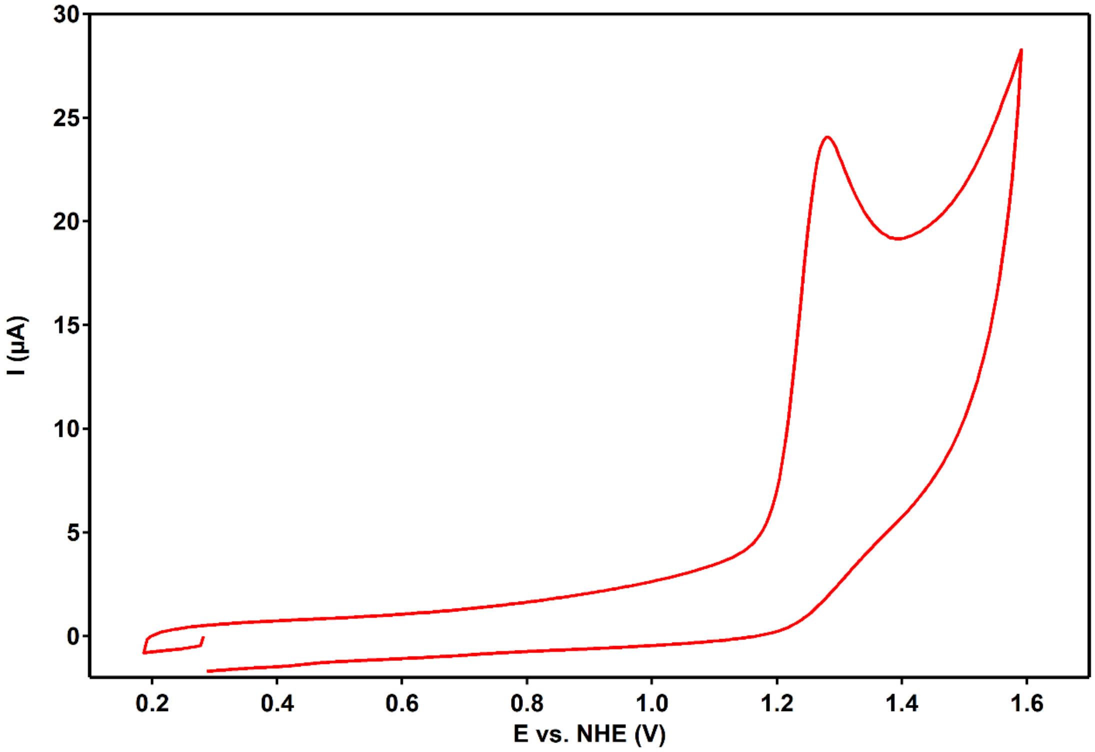

Figure S14. Cyclic voltammogram 0.5 mM Zn(H**L**) in 100 mM pH 7 phosphate buffer at a scan rate of 100 mV/s. GC, Au and RHE were used as WE, CE and RE, respectively. Potentials were converted to NHE.

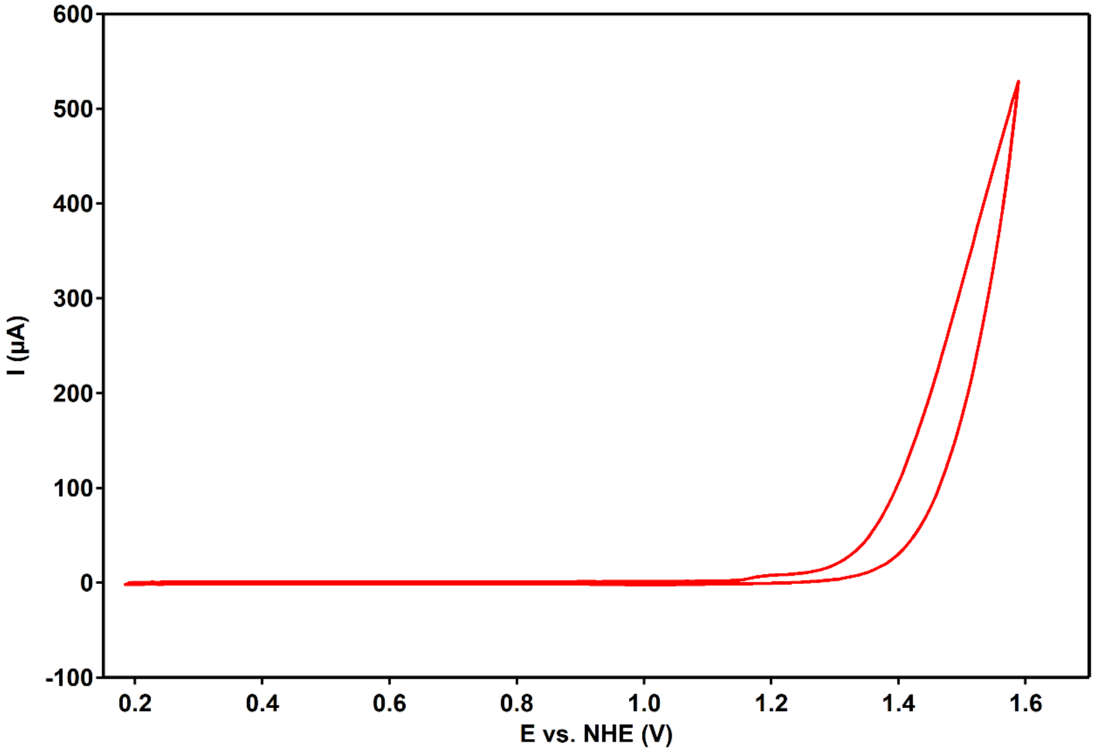

Figure S15. Cyclic voltammogram of 0.5 mM $Co(NO_3)_2$ in a 100 mM pH 7 phosphate buffer at a scan rate of 100 mV/s. GC, Au and RHE were used as WE, CE and RE, respectively. Potentials were converted to NHE.

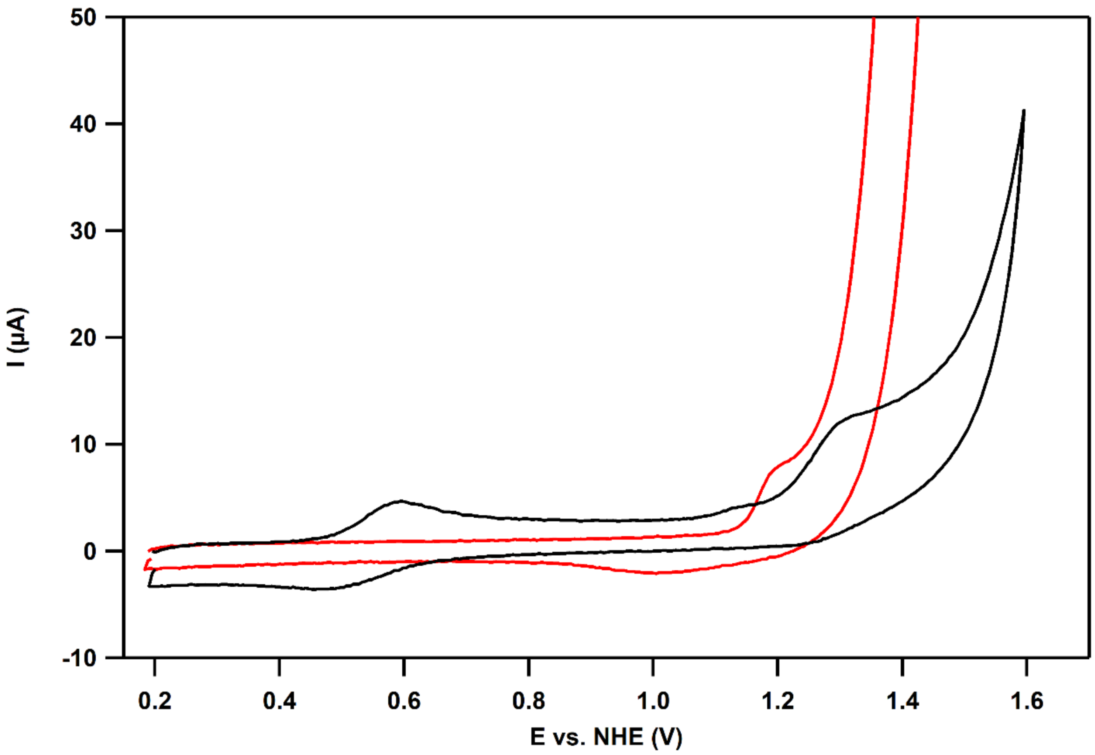

Figure S16. Cyclic voltammograms 0.5 mM Co(H**L**) (black) and 0.5 mM $Co(NO_3)_2$ (red) in 100 mM pH 7 phosphate buffer at a scan rate of 100 mV/s. GC, Au and RHE were used as WE, CE and RE, respectively. Potentials were converted to NHE.

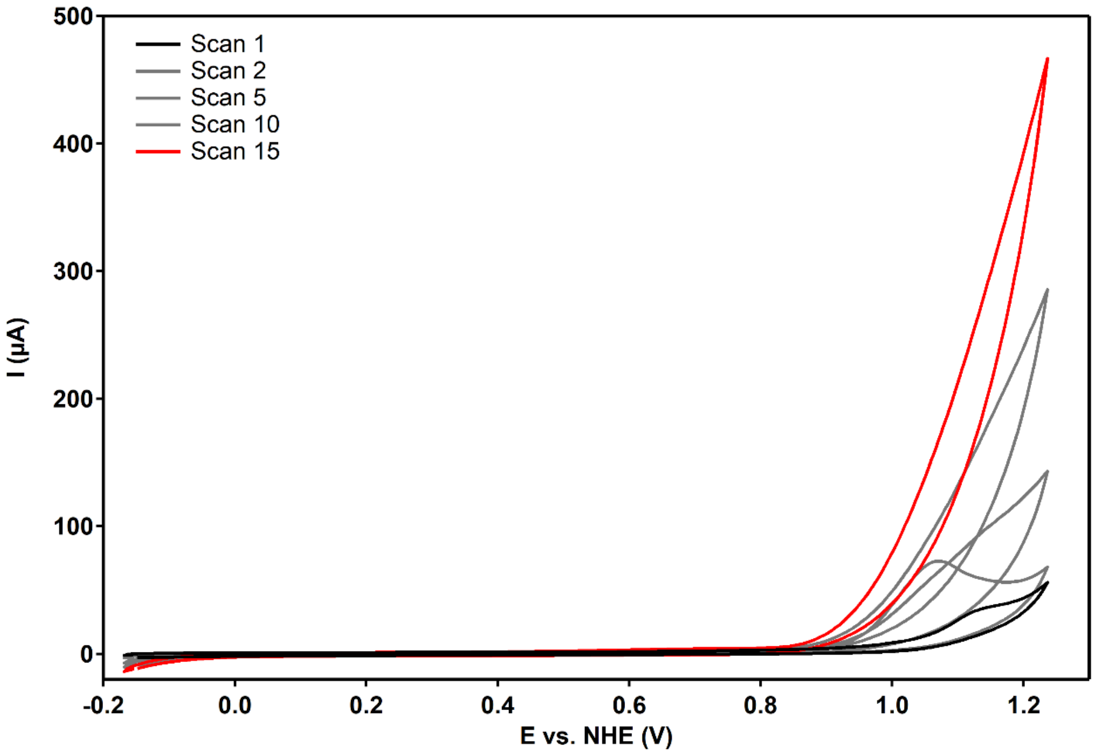

Figure S17. Cyclic voltammograms of 0.5 mM [Co(**L**) in 100 mM NaOH, pH 13 at a scan rate of 100 mV/s. GC, Au and RHE were used as WE, CE and RE, respectively. Potentials were converted to NHE.

Dipping test and post catalysis study

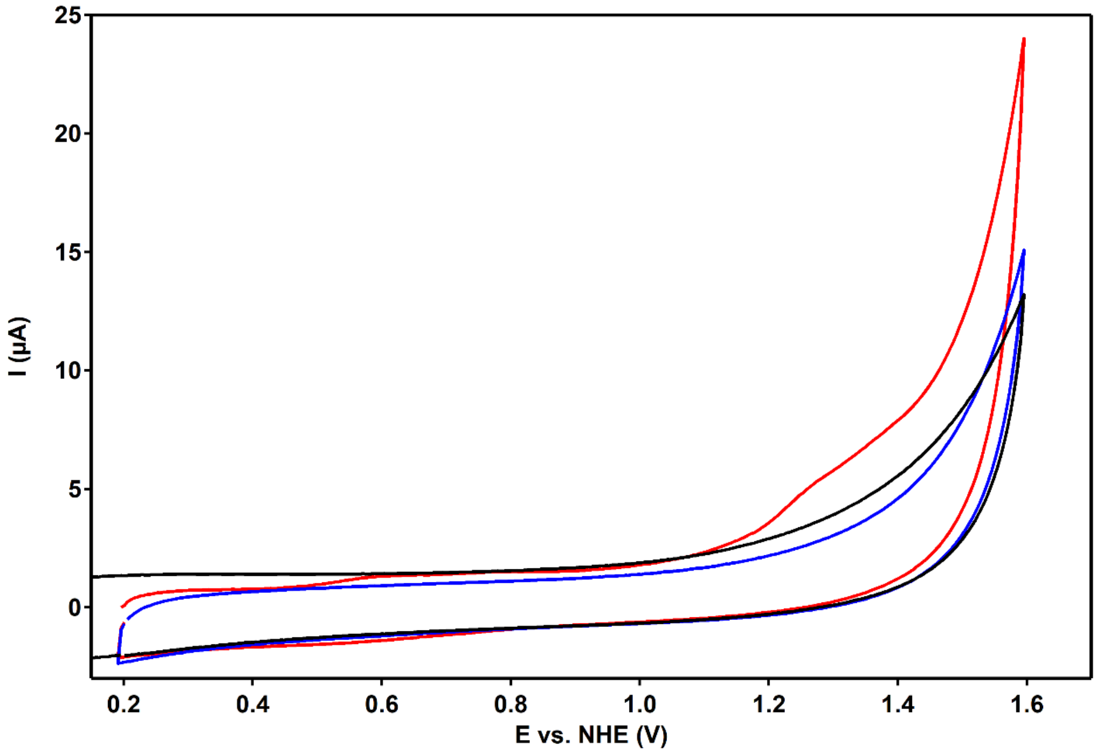

Figure S18. Cyclic voltammograms in 100 mM pH 7 phosphate buffer prior (black) and after (cycle 1: red, cycle 10: blue), 10 cycles between 0.2 and 0.7 V vs. NHE of 0.5 mM Co(H**L**) in a 100 mM pH 7 phosphate buffer at a scan rate of 100 mV/s. GC, Au and RHE were used as WE, CE and RE, respectively. Potentials were converted to NHE.

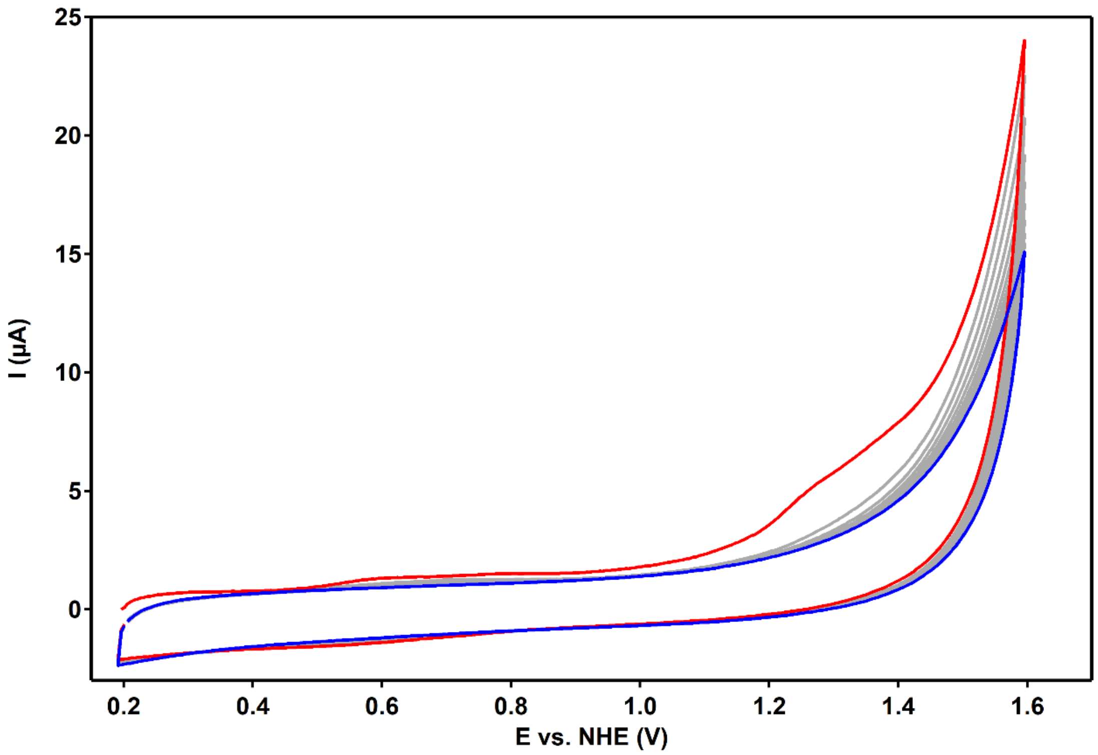

Figure S19. Cyclic voltammograms of 10 cycles (cycle 1: red, cycle 10: blue) in a 100 mM pH 7 phosphate buffer at a scan rate of 100 mV/s after 10 cycles between 0.2 and 0.7 V vs. NHE of 0.5 mM Co(H**L**) in a 100 mM pH 7 phosphate. GC, Au and RHE were used as WE, CE and RE, respectively. Potentials were converted to NHE.

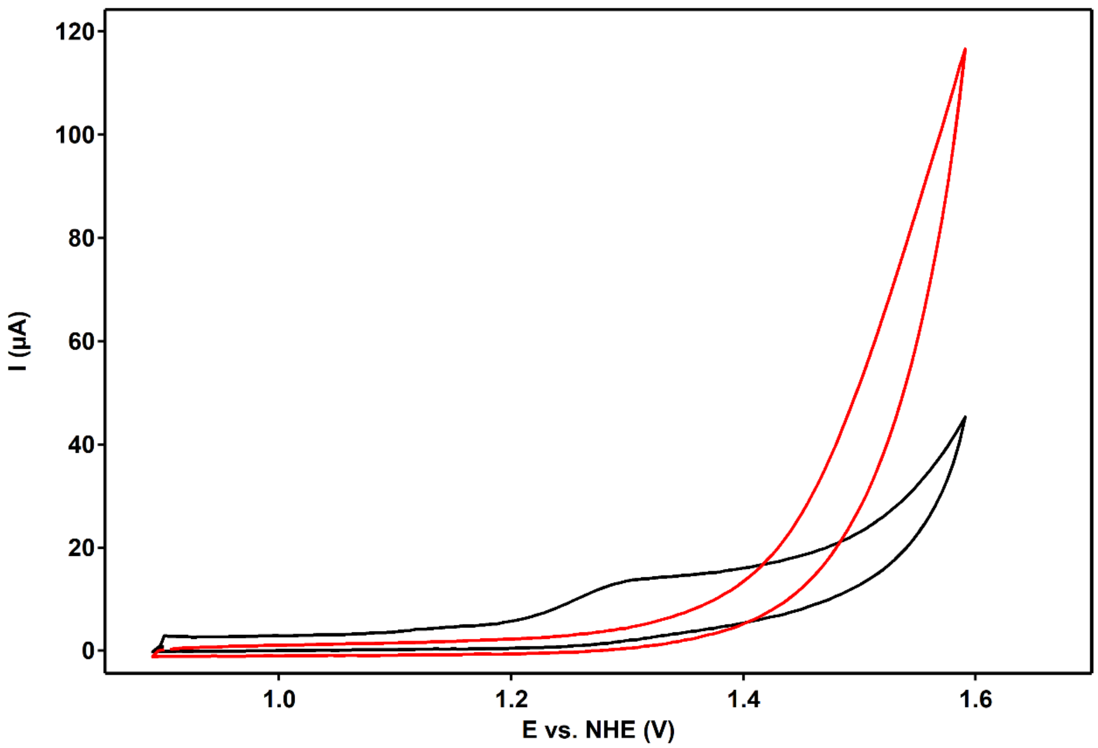

Figure S20. Cyclic voltammograms of cycle 1 (black) and 75 (red) of 0.5 mM Co(H**L**) in a 100 mM pH 7 phosphate buffer at a scan rate of 100 mV/s. GC, Au and RHE were used as WE, CE and RE, respectively. Potentials were converted to NHE.

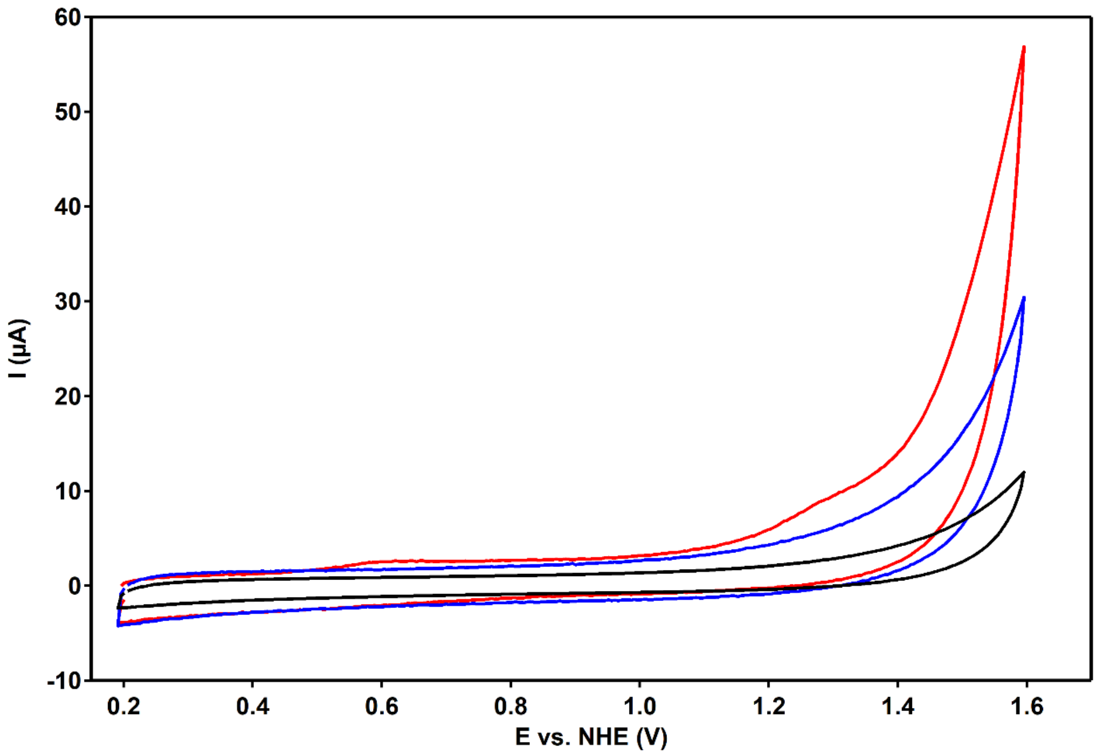

Figure S21. Cyclic voltammograms in a 100 mM pH 7 phosphate buffer at a scan rate of 100 mV/s prior (black) and after (cycle 1: red, cycle 50: blue), 75 cycles between 0.9 and 1.6 V vs. NHE of 0.5 mM Co(H**L**) in a 100 mM pH 7 phosphate buffer. GC, Au and RHE were used as WE, CE and RE, respectively. Potentials were converted to NHE.

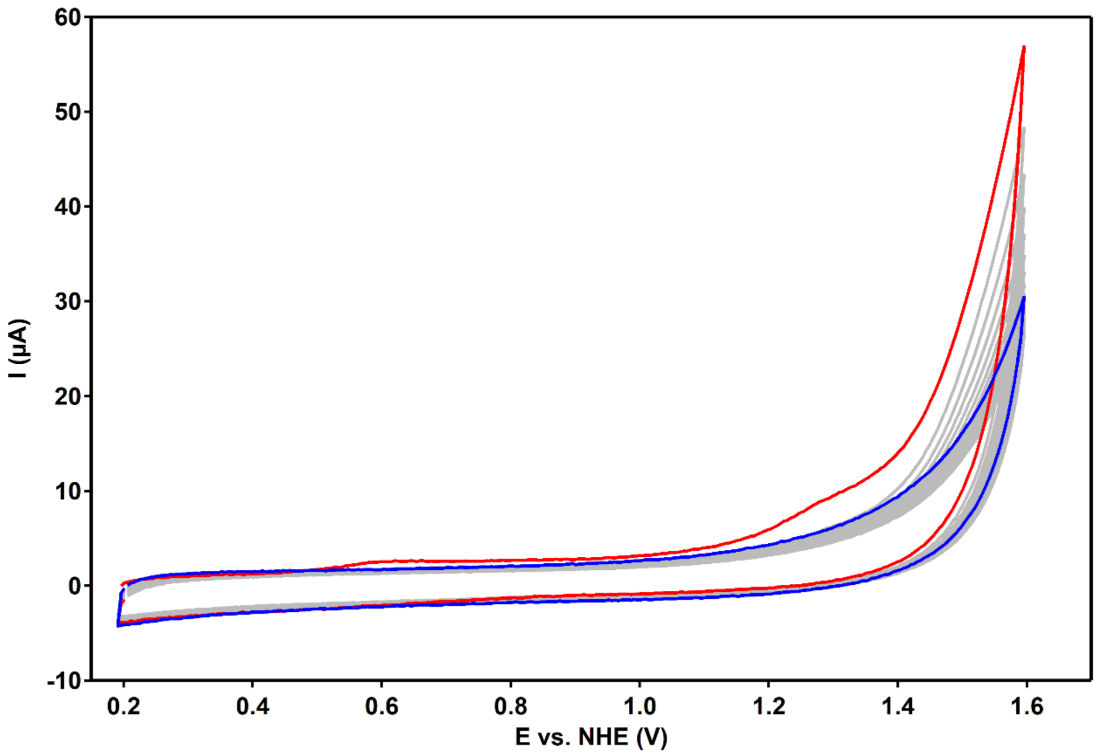

Figure S22. Cyclic voltammograms of 50 cycles in a 100 mM pH 7 phosphate buffer at a scan rate of 100 mV/s after 75 cycles after (cycle 1: red, cycle 50: blue) between 0.9 and 1.6 V vs. NHE of 0.5 mM Co(H**L**) in a 100 mM pH 7 phosphate buffer. GC, Au and RHE were used as WE, CE and RE, respectively. Potentials were converted to NHE.

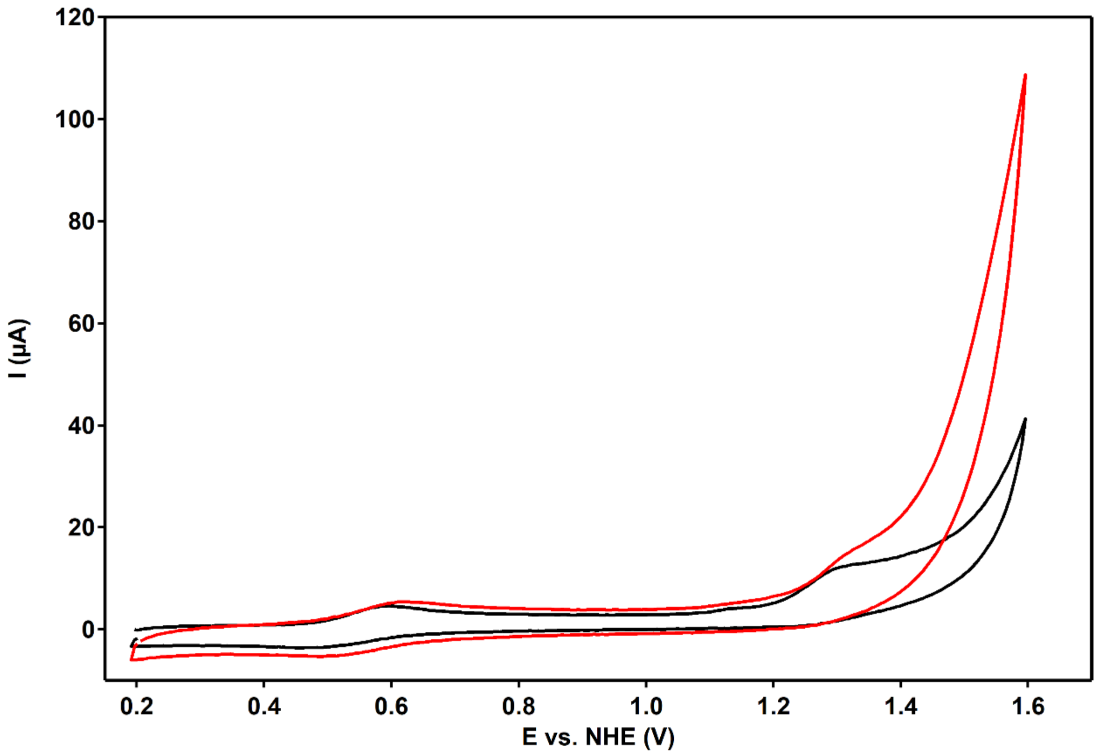

Figure S23. Cyclic voltammograms of cycle 1 (black) and 75 (red) of 0.5 mM Co(H**L**) in a 100 mM pH 7 phosphate buffer at a scan rate of 100 mV/s. GC, Au and RHE were used as WE, CE and RE, respectively. Potentials were converted to NHE.

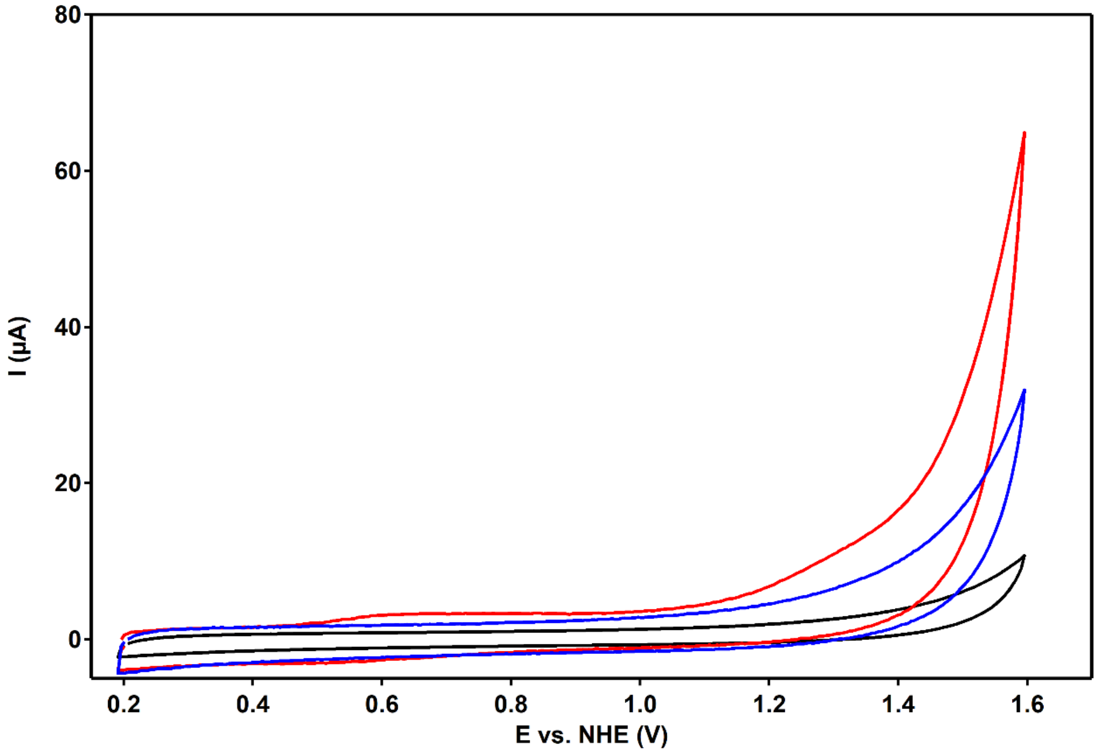

Figure S24. Cyclic voltammograms in a 100 mM pH 7 phosphate buffer at a scan rate of 100 mV/s prior (black) and after (cycle 1: red, cycle 50: blue), 75 cycles between 0.2 and 1.6 V vs. NHE of 0.5 mM Co(H**L**) in a 100 mM pH 7 phosphate buffer. GC, Au and RHE were used as WE, CE and RE, respectively. Potentials were converted to NHE.

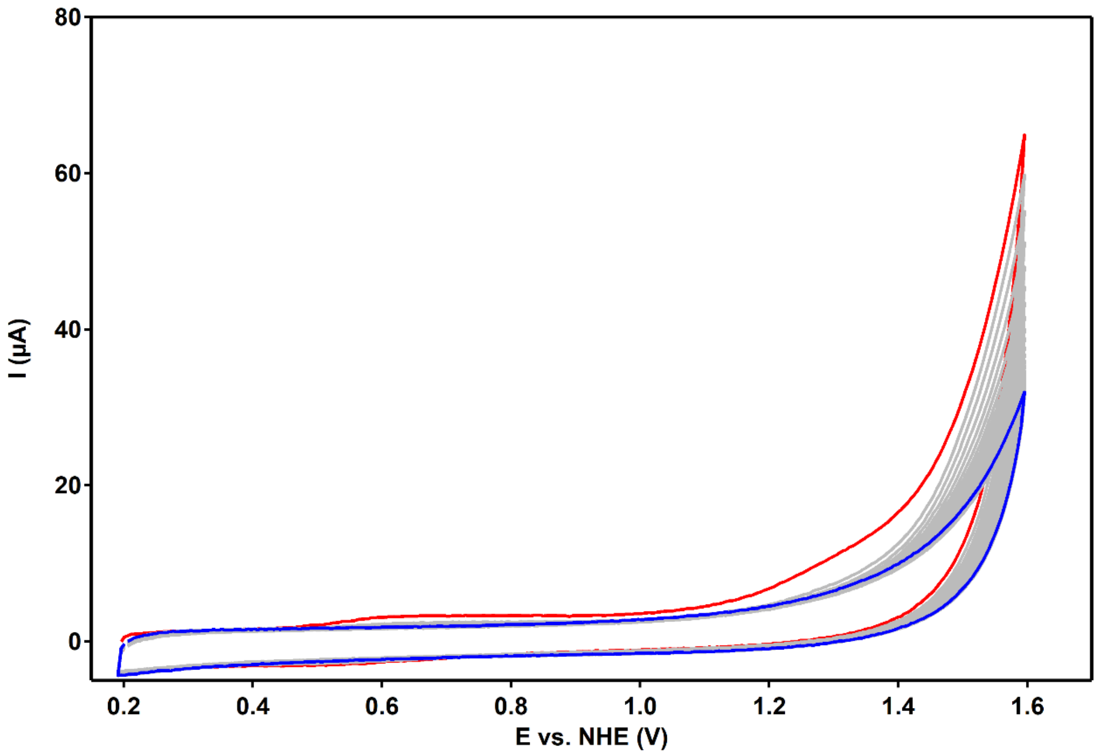

Figure S25. Cyclic voltammograms of 50 cycles in a 100 mM pH 7 phosphate buffer at a scan rate of 100 mV/s after 75 cycles after (cycle 1: red, cycle 50: blue) between 0.2 and 1.6 V vs. NHE of 0.5 mM Co(H**L**) in a 100 mM pH 7 phosphate buffer. GC, Au and RHE were used as WE, CE and RE, respectively. Potentials were converted to NHE.

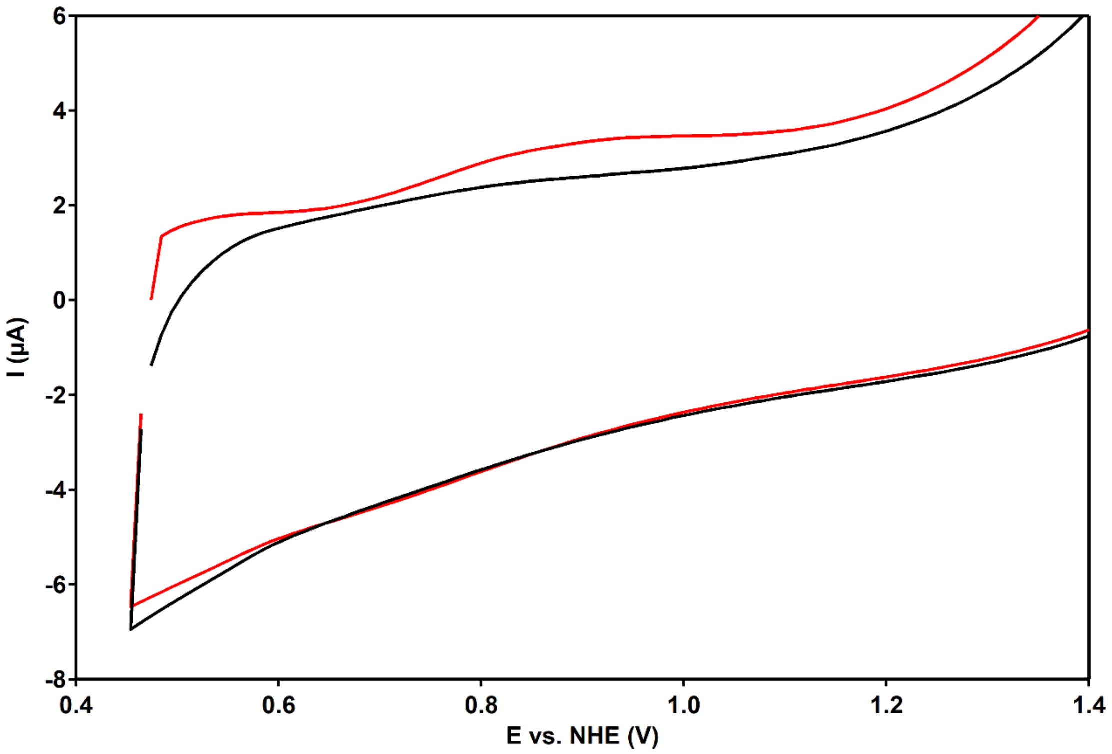

Figure S26. Cyclic voltammograms of 2 consecutive cycles (cycle 1: red, cycle 2: black) in a 100 mM pH 2.5 phosphate buffer at a scan rate of 100 mV/s after 75 cycles after between 0.2 and 1.6 V vs. NHE of 0.5 mM Co(H**L**) in a 100 mM pH 7 phosphate buffer. GC, Au and RHE were used as WE, CE and RE, respectively. Potentials were converted to NHE. The redox activity of the Co(H**L**) was absent in the second scan.

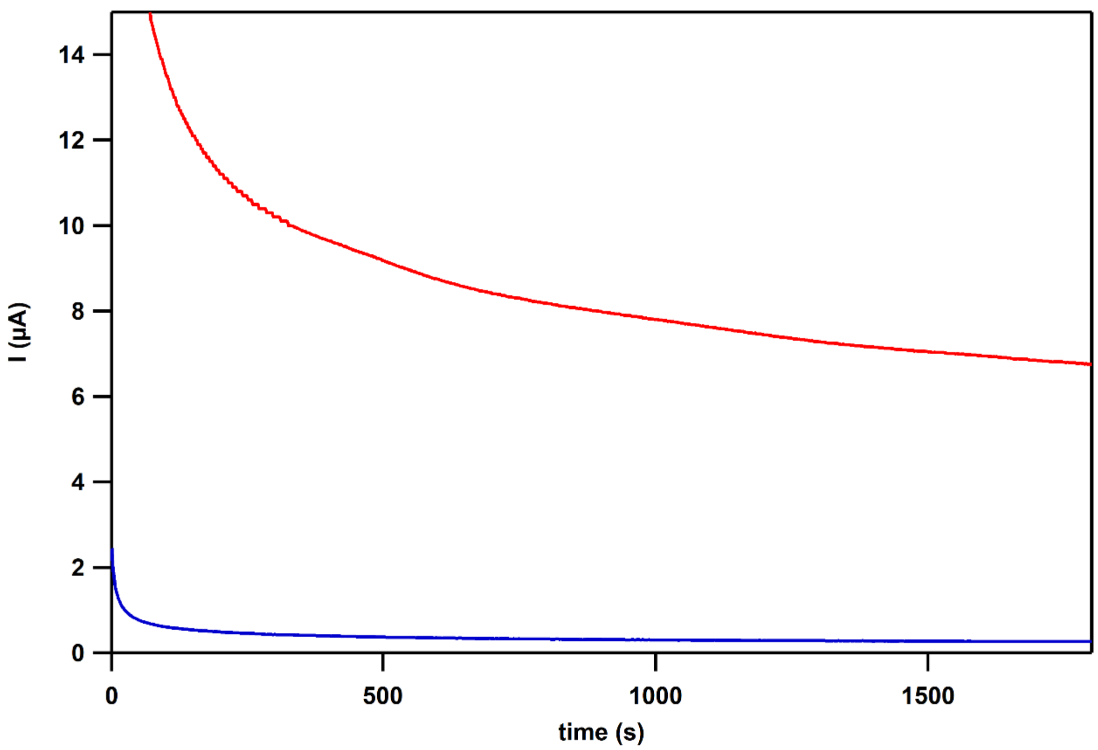

Figure S27. Chronoamperogram at 1.29 V vs. NHE in a 100 mM pH 7 phosphate buffer of Co(H**L**) (blue) and Co-Pi (red) deposits produced by 75 cycles of cyclic voltammetry between 0.9 and 1.6 V vs. NHE in a 0.5 mM [Co] in a 100 mM pH 7 phosphate buffer with a scan rate of 100 mV/s. GC, Au and RHE were used as WE, CE and RE, respectively.

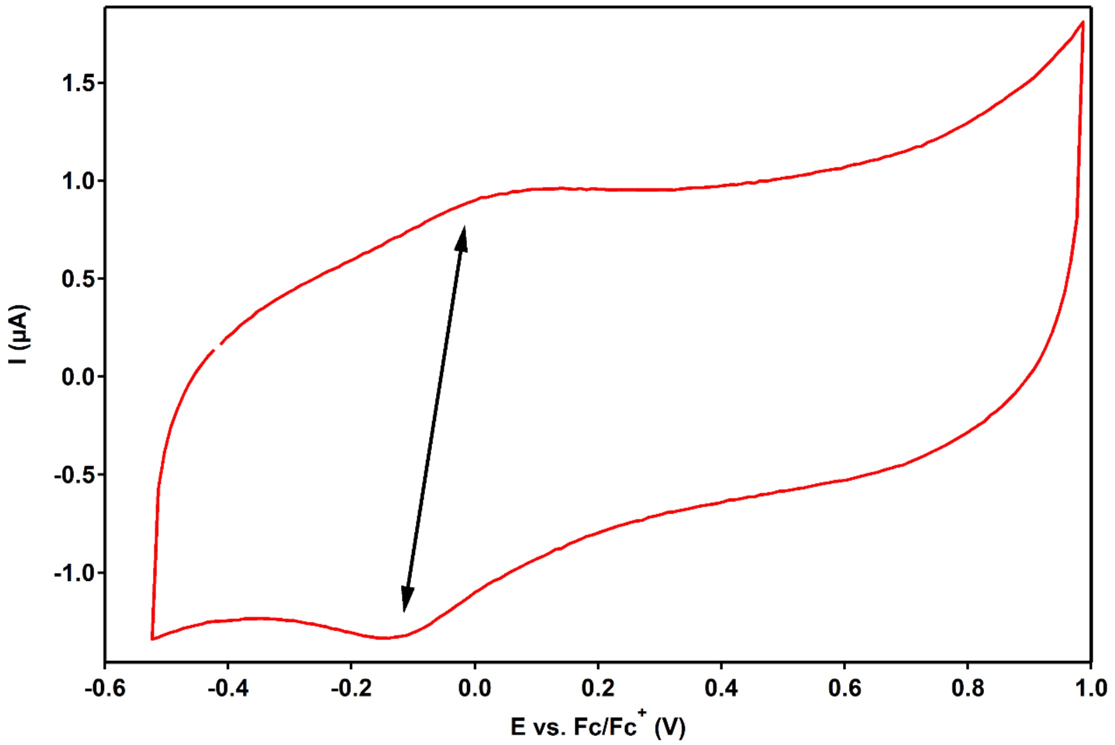

Figure S28. Cyclic voltammogram in 0.1 M NBu_4PF_6 in MeCN at a scan rate of 100 mV/s of a deposition of Co(H**L**) produced by 75 cycles of cyclic voltammetry between 0.9 and 1.6 V vs. NHE in a 0.5 mM Co(H**L**) in a 100 mM pH 7 phosphate buffer with a scan rate of 100 mV/s. GC, Au and Ag/AgCl were used as WE, CE and RE, respectively. Potentials were converted to the ferrocene redox couple (Fc/Fc^+).

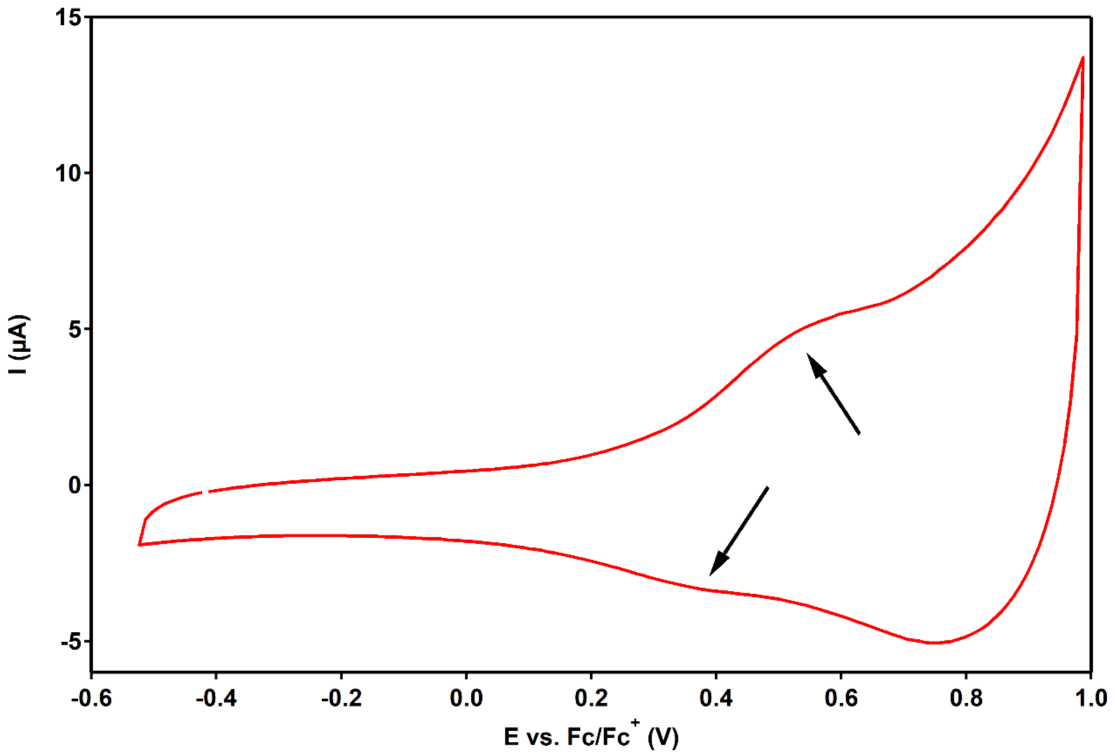

Figure S29. Cyclic voltammogram in 0.1 M NBu_4PF_6 in MeCN at a scan rate of 100 mV/s of a deposition of Co-Pi produced by 75 cycles of cyclic voltammetry between 0.9 and 1.6 V vs. NHE in a 0.5 mM $Co(NO_3)_2$ in a 100 mM pH 7 phosphate buffer with a scan rate of 100 mV/s. GC, Au and Ag/AgCl were used as WE, CE and RE, respectively. Potentials were converted to the ferrocene redox couple (Fc/Fc^+).

Electrochemical quartz crystal microbalance

To determine the amount of deposition on the electrode surface, the Δ frequency (Δf) can be converted to Δ mass (Δm) using the Sauerbrey equation:[7]

$$-\Delta f = \mathrm{C_f} \cdot \Delta \mathrm{m}$$

Here Δf is the frequency change in Hz, C_f is the sensitivity coefficient of the quartz crystal in $Hz/g/cm^2$ and Δm is the mass change in g/cm^2.

The C_f value was determined by a Pb bulk deposition experiment from a 10 mM solution of $Pb(NO_3)_2$ in 0.1 M $HClO_4$ (Figure S30). A Cyclic voltammogram was recorded, starting at –0.54 V and 1.06 V, starting at 0.86 V vs. RHE. The integrated charge was determined during the deposition of bulk Pb on the Au electrode, which occurred between 0.1 and –0.54 V vs. RHE. The integrated charge was converted to Δm by the following conversion:

$$\Delta \mathrm{m} = \frac{\mathrm{Q}}{\mathrm{n} * \mathrm{F}} * \frac{\mathrm{M}}{\mathrm{A}}$$

Here Q is the charge in C, n the number of electrons, which was 2 in this case ($Pb^{2+} + 2\ e^- \rightarrow Pb$), F is the Faraday constant of 96485 C/mol), M is the molar mass in g/mol which was 207.2 g/mol in this case and A is the surface area of the electrode, which was 0.35 cm^2 in this case. A faradaic efficiency of 100% is assumed for the deposition.

C_f was determined by the average value of $\Delta f/\Delta m$ between 25 and 36 seconds during the bulk deposition experiment. Here we found a value of $8.54 \cdot 10^7$ $Hz/g/cm^2$, which corresponds to the value of $8.15 \cdot 10^7$ $Hz/g/cm^2$ provided by the manufacturer.[8]

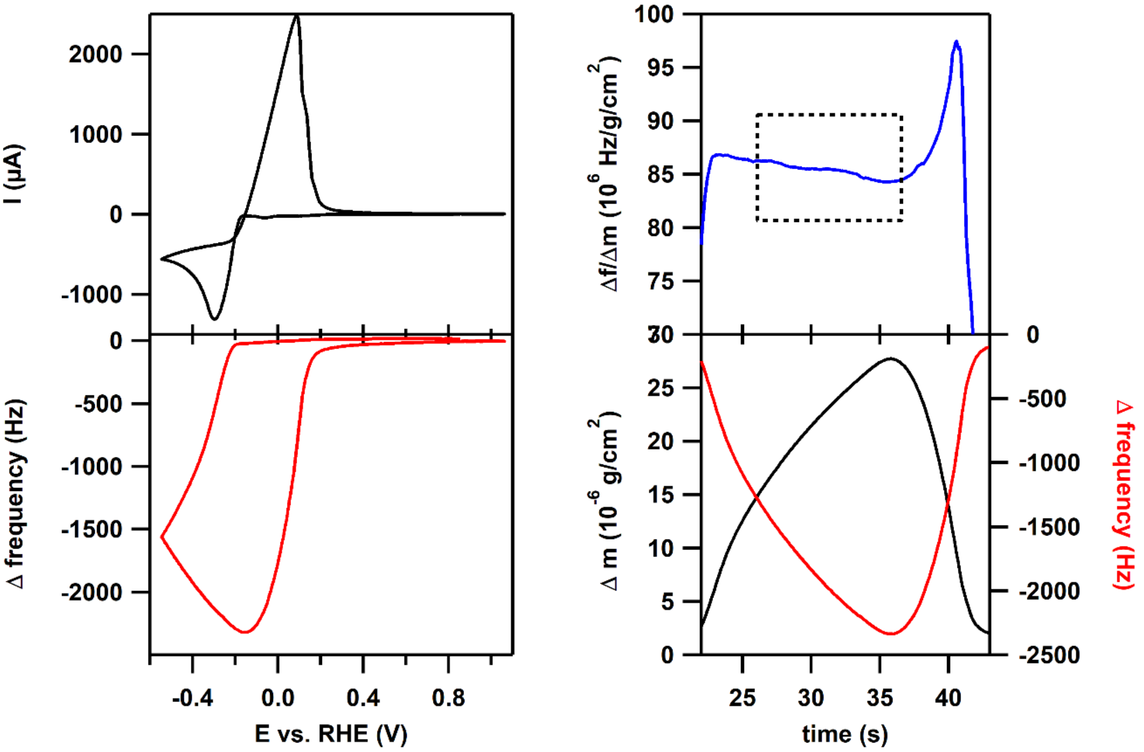

Figure S30. Calibration of the sensitivity coefficient (C_f) by bulk deposition on a Au EQCM electrode. **Left)** Cyclic voltammogram in combination with EQCM of 10 mM $Pb(NO_3)_2$ in 0.1 M $HClO_4$ recorded at 50 mV/s. In the top part, the cyclic voltammogram is shown and in the bottom part the Δ frequency (red) response. Au, Au and RHE were used as WE, CE and RE, respectively. **Right)** In the bottom part the Δ frequency (red) and Δ mass response (black) between 0.1 and –0.54 V vs. RHE and back in time is shown. In the top panel $\Delta f/\Delta m$ (blue) is plotted over the same time period. The average value between 25 and 36 seconds was used to determine sensitivity coefficient (black dotted box).

To get an indication how many molecules and how many layers of Co(H**L**) are roughly adsorbed on the surface, the amount of mol per cm^2 was estimated from the crystal packing (Table S1). Using 7.40 and 8.51 Å, the size of one Co(H**L**) particle is of $6.30 \cdot 10^{-15}$ cm^2. The amount of Co(H**L**) particles per cm^2 then corresponded to $1.58 \cdot 10^{14}$ particles per cm^2, which is $2.63 \cdot 10^{-10}$ mol/cm^2 for a monolayer, assuming a structured deposition of Co(H**L**) with the ligand arranged planer to the electrode surface. The species in solution is expected to be $[Co(HL)(H_2O)_2]^{2+}$ (M = 420.3 g/mol). Utilizing the Sauerbrey equation discussed above, Δf can be calculated for a monolayer of deposited Co(H**L**), which is roughly 10 Hz.

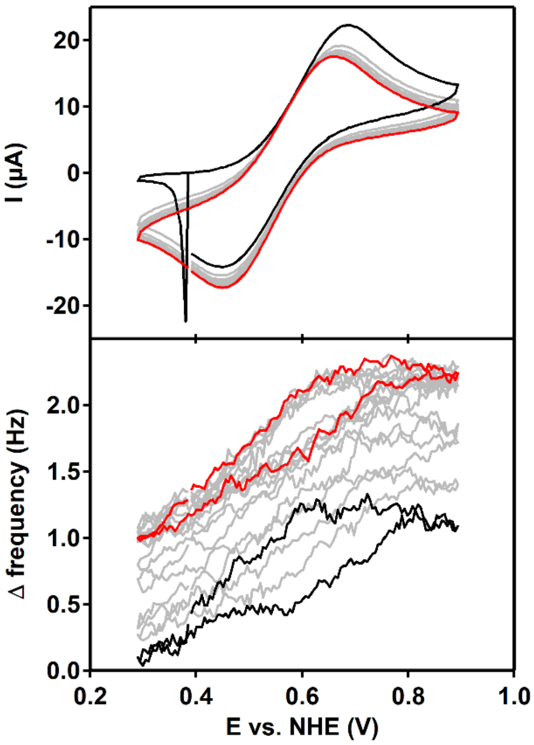

Figure S31. Cyclic voltammograms in combination with EQCM including 10 cycles. Top: cyclic voltammogram of 0.5 mM Co(H**L**) in a 100 mM pH 7 phosphate buffer at a scan rate of 100 mV/s. Bottom: Δ frequency response. Scan 1: black line, scan 10: red line. Au, Au and RHE were used as WE, CE and RE, respectively. Potentials were converted to NHE.

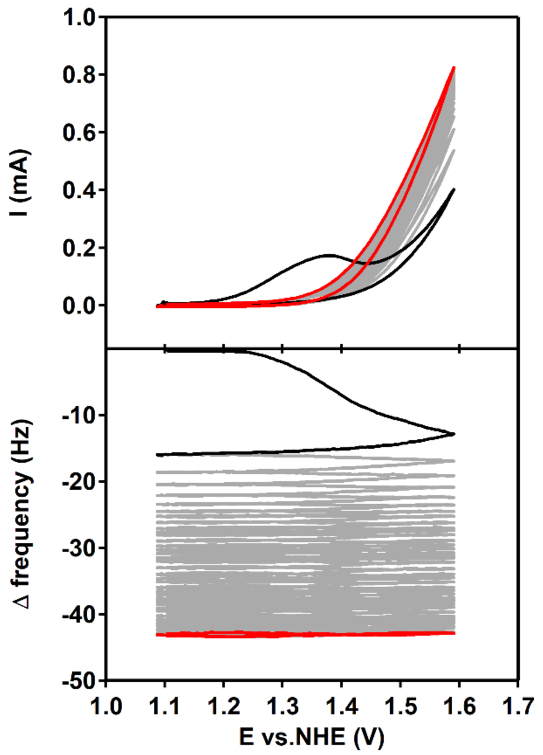

Figure S32. Cyclic voltammogram in combination with EQCM including 50 cycles. Top: cyclic voltammogram of 0.5 mM Co(H**L**) in a 100 mM pH 7 phosphate buffer at a scan rate of 100 mV/s. Bottom: Δ frequency response. Scan 1: black line, scan 50: red line. Au, Au and RHE were used as WE, CE and RE, respectively. Potentials were converted to NHE.

^{31}P NMR

^{31}P NMR samples were prepared as following; a 100 mM pH 7 phosphate buffer was prepared by dissolving NaH_2PO_4 and Na_2HPO_4 in D_2O, which was also used as reference sample. Two solutions of 500 µM (0.5 mM) of Co(H**L**) and $Co(NO_3)_2$ dissolved in phosphate buffer (D_2O). The $Co(NO_3)_2$ solution was diluted to a 50 and 5 µM $Co(NO_3)_2$ solution. Two samples were prepared containing 0.5 mM Co(H**L**) solution spiked with 5 and 0.5 µM $Co(NO_3)_2$. These NMR samples are in good agreement with used solutions for electrochemistry experiments.

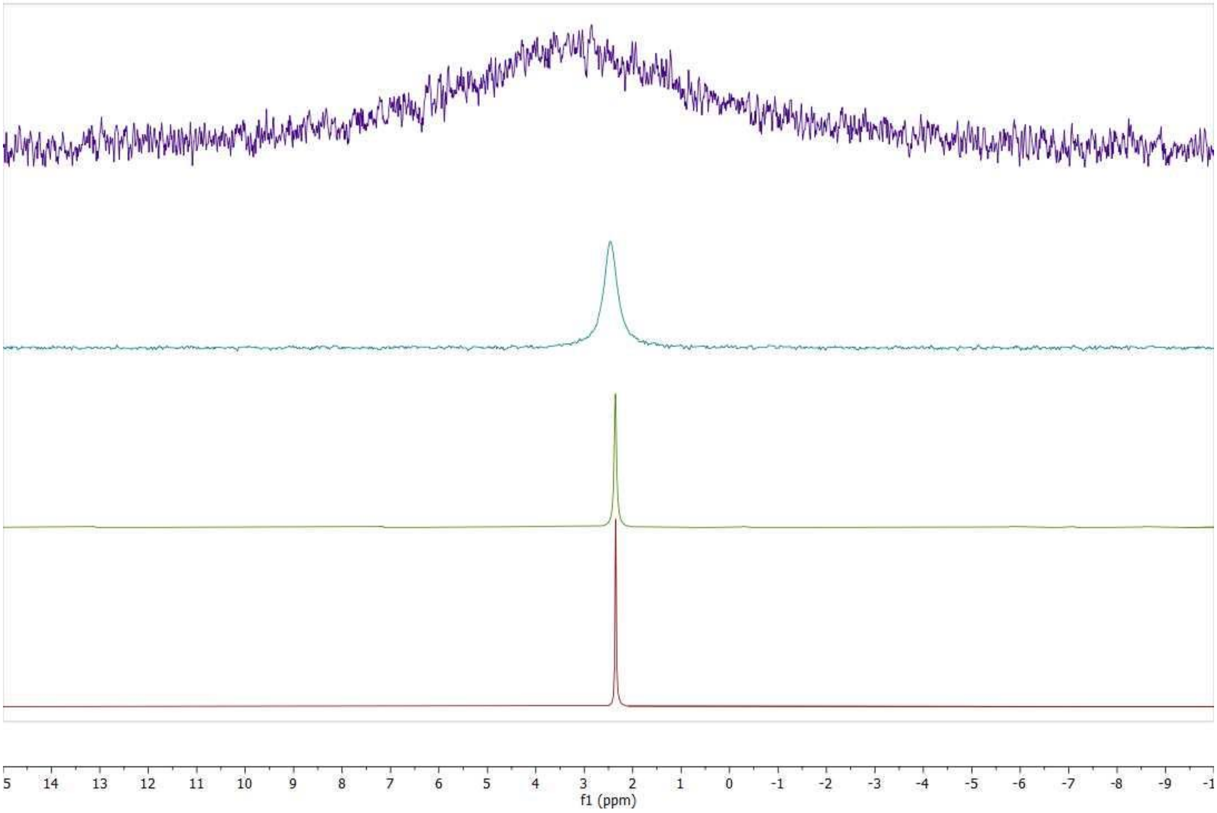

Figure S33. ^{31}P NMR of varying concentrations of $Co(NO_3)_2$ in 100 mM phosphate buffer in D_2O. Top to bottom: 500, 50, 5 and 0 µM $Co(NO_3)_2$.

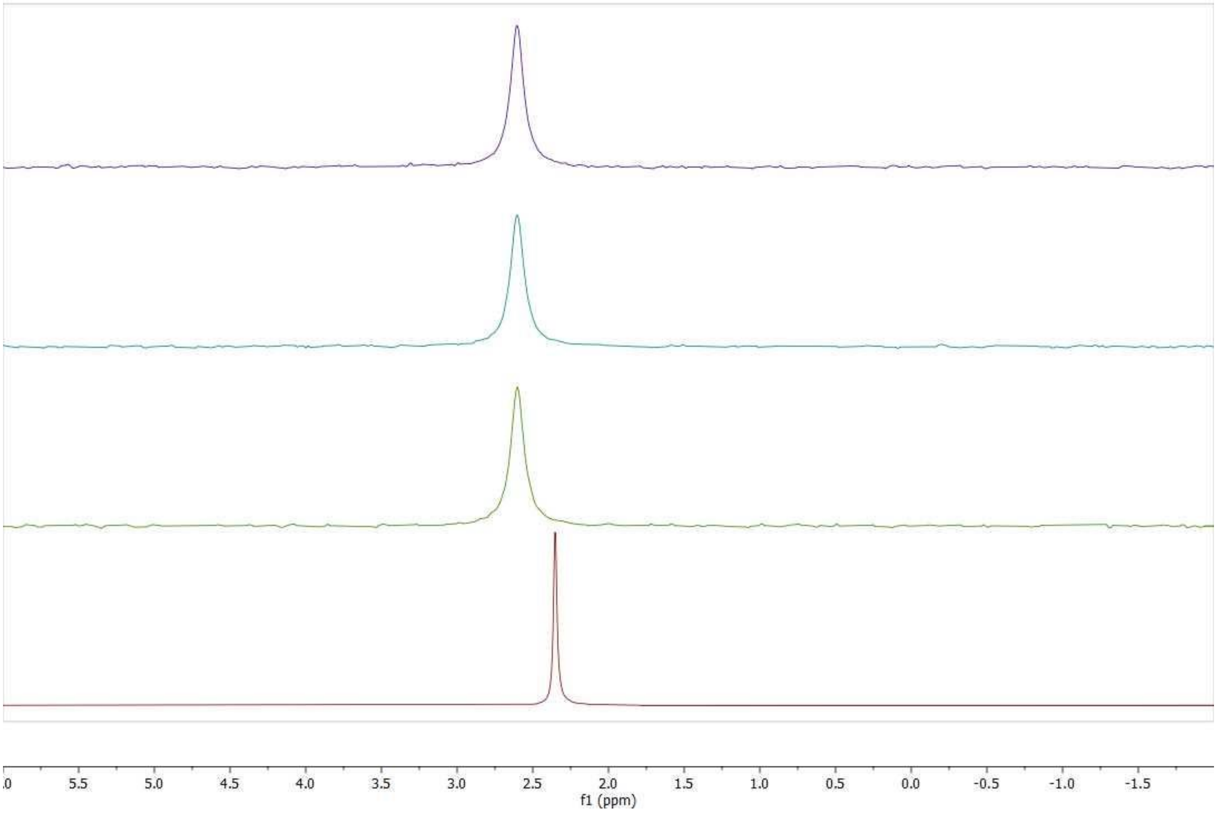

Figure S34. ^{31}P NMR of varying concentrations of Co(H**L**) and $Co(NO_3)_2$ in 100 mM phosphate buffer in D_2O. Top to bottom: 1) 500 µM Co(H**L**) 2) 500 µM Co(H**L**) and 5 µM $Co(NO_3)_2$ 3) 500 µM Co(H**L**) and 0.5 µM $Co(NO_3)_2$ 4) phosphate buffer.

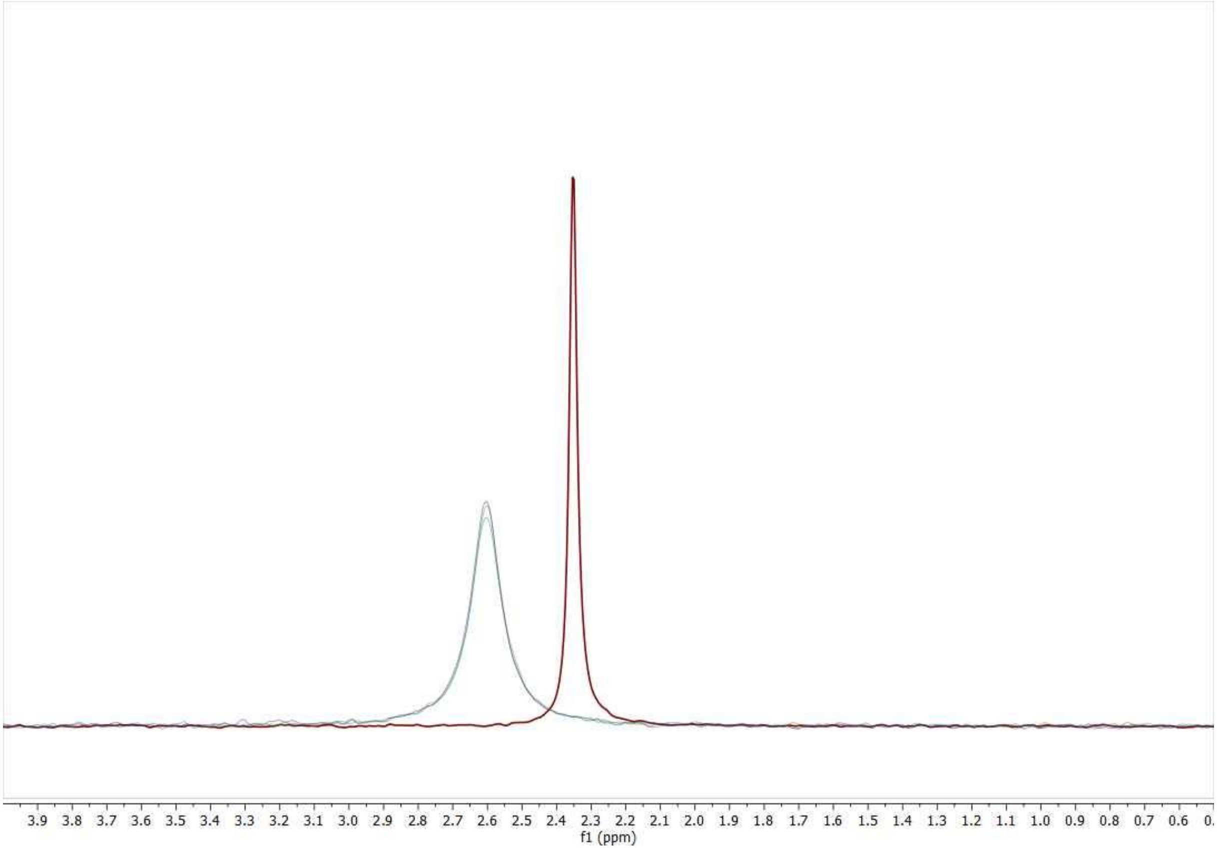

Figure S35. ^{31}P NMR of 500 µM Co(H**L**) with 0, 5 and 0.5 µM $Co(NO_3)_2$ in 100 mM phosphate buffer in D_2O. The red peak shows the ^{31}P NMR spectrum without Co present.

Co^{2+} trapping with EDTA

To a 0.5 mM Co(H**L**) solution in 100 mM pH 7 buffer amounts of Na_2H_2EDTA were added to potentially bind to free Co^{2+}. CVs were recorded without and with varying concentrations of Na_2H_2EDTA. First, a CV was recorded without addition of Na_2H_2EDTA. Subsequently, 5, 10 and 20% Na_2H_2EDTA vs. 0.5 mM Co(H**L**) were added, to the solution and a new CV was recorded (Figure 27). In between, the electrode was polished to start every measurement with a clean electrode surface. Over several cycles, no depletion of catalytic activity was observed. The current slightly increase with the Na_2H_2EDTA concentration, which is probably caused by background activity of Na_2H_2EDTA itself (Figure S28). The current in the blank increased linearly with the % EDTA, indicating Na_2H_2EDTA is not electrochemically inert under these conditions (Figure 29).

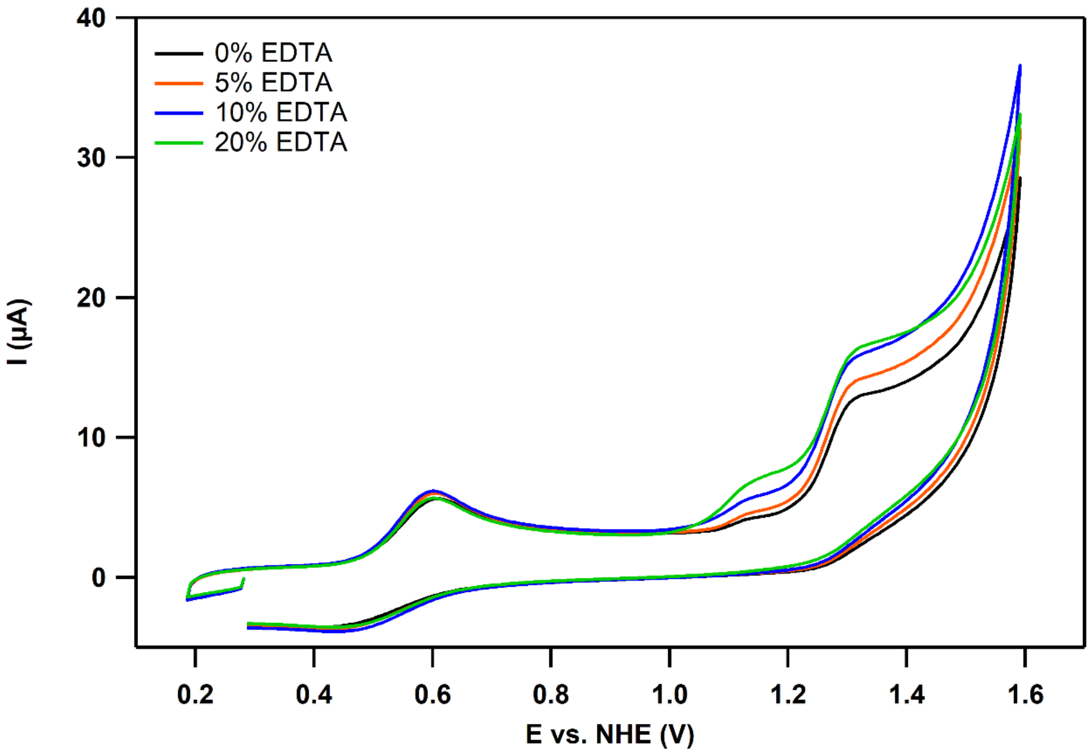

Figure S36. Cyclic voltammograms of 0.5 mM Co(H**L**) in 100 mM pH 7 phosphate buffer at a scan rate of 100 mV/s with varying % of Na_2H_2EDTA. GC, Au and RHE were used as WE, CE and RE, respectively. Potentials were converted to NHE.

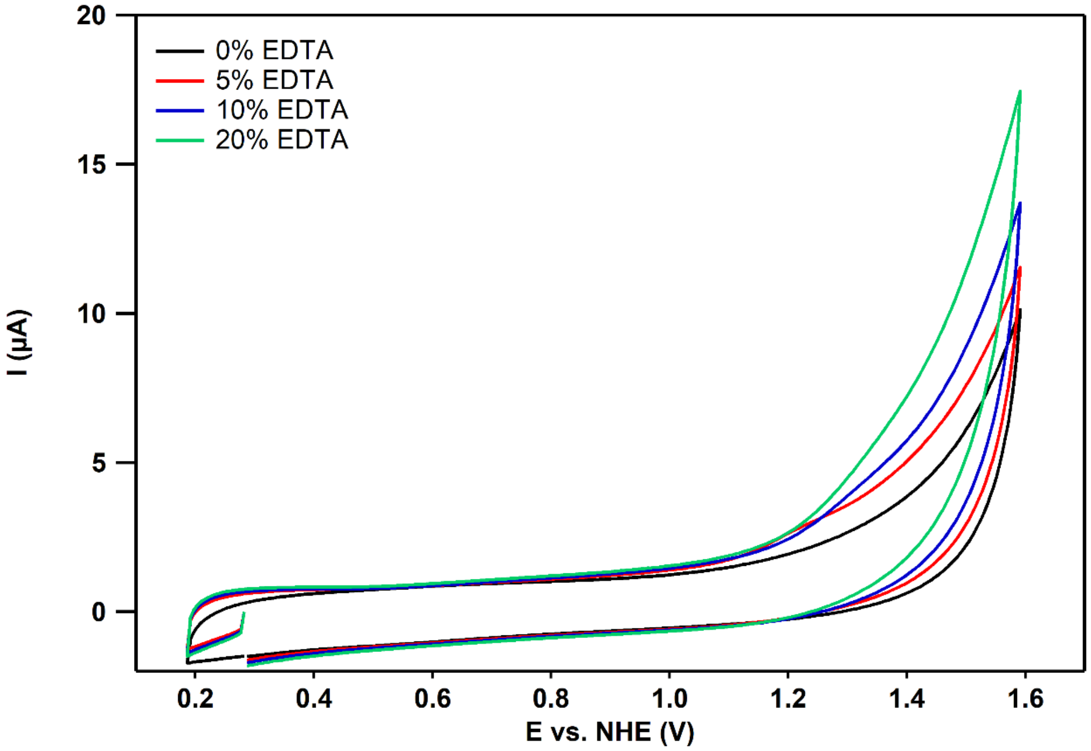

Figure S37. Cyclic voltammogram of varying % of Na_2H_2EDTA in 100 mM pH 7 phosphate buffer at a scan rate of 100 mV/s. GC, Au and RHE were used as WE, CE and RE, respectively. Potentials were converted to NHE.

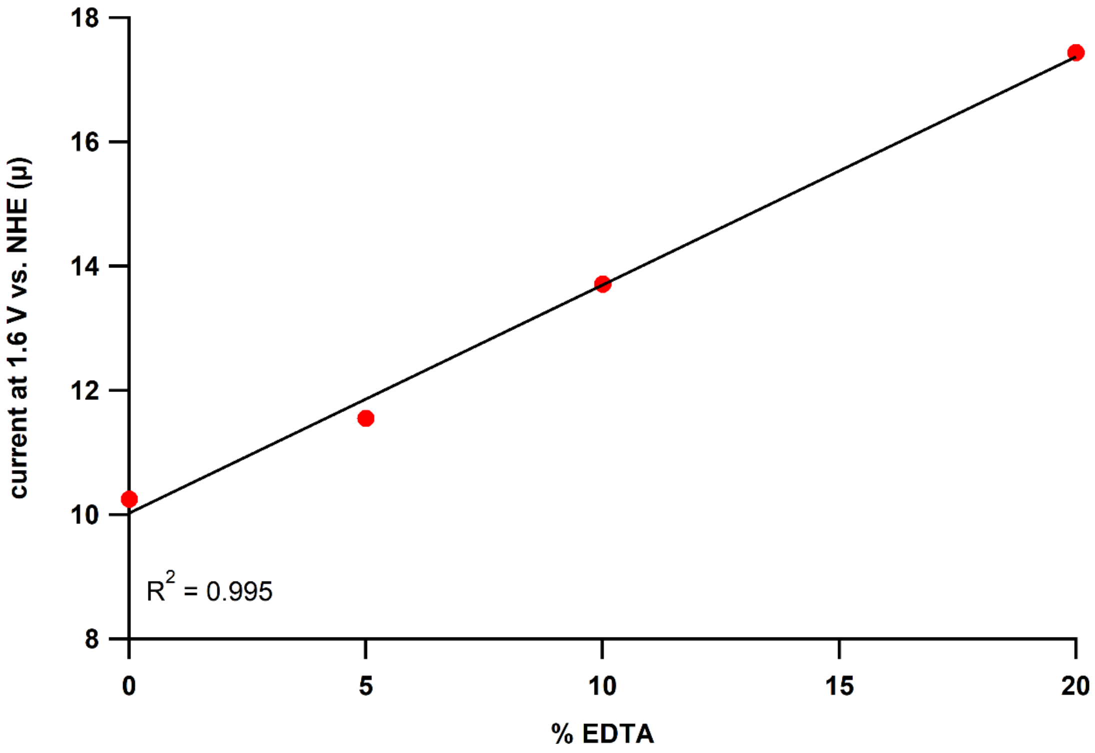

Figure S38. Linear correlation between the % Na_2H_2EDTA and current obtained at 1.6 V vs. NHE in the blank cyclic voltammogram.

Cyclic voltammograms after chronoamperometry

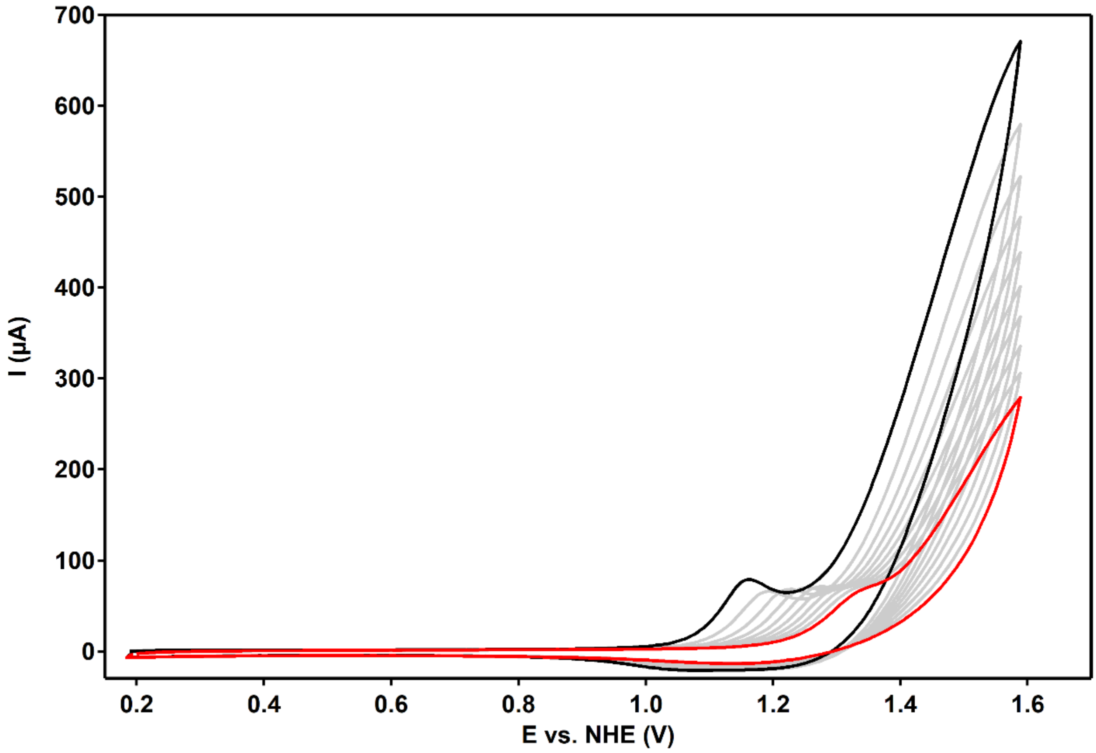

Figure S39. Cyclic voltammograms of 10 cycles in a 100 mM pH 7 phosphate buffer at a scan rate of 100 mV/s recorded after chronoamperometry in 0.5 mM $Co(NO_3)_2$ in a 100 mM pH 7 phosphate at 1.25 V vs. NHE until a charge of 60 mC/cm^2 has passed (scan 1: black, scan 10: red). GC, Au and RHE were used as WE, CE and RE, respectively. Potentials were converted to NHE.

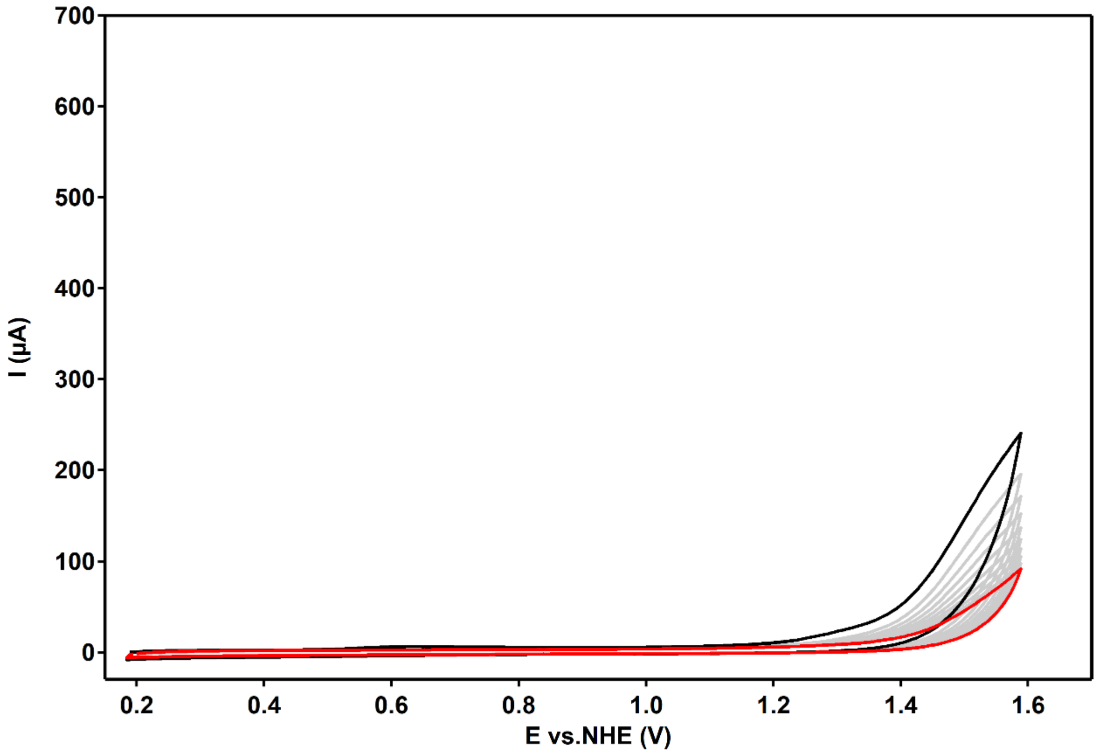

Figure S40. Cyclic voltammograms of 10 cycles in a 100 mM pH 7 phosphate buffer at a scan rate of 100 mV/s recorded after chronoamperometry in 0.5 mM Co(H**L**) in a 100 mM pH 7 phosphate at 1.25 V vs. NHE until a charge of 60 mC/cm^2 has passed (scan 1: black, scan 10: red). GC, Au and RHE were used as WE, CE and RE, respectively. Potentials were converted to NHE.

Oxygen detection and Faradaic efficiency

The Neofox oxygen probe was calibrated by a 2-point calibration consisting of 0% and 100% oxygen in solution by placing the probe in de argon deaerated solution and an O_2-saturated solution. Here the 100% calibration corresponds with 1.1 mM of O_2 in solution. [9] The amount of oxygen formed can then be determined relative to the value belonging to a 1.1 mM concentration of O_2 in solution, denoted as $\%O_2$. The electrolyte was deaerated with argon prior to the experiment and argon was flowed continuously over the cell during the experiment to prevent dissolving O_2 from the air. An experiment was performed as follow, the $\%O_2$ was measured for a few minutes to obtain a stable baseline. Subsequently, 1.29 V vs. Ag/AgCl (= 1.49 V vs. NHE) was applied for 10 minutes and afterwards the $\%O_2$ was measured for another few minutes to obtain the maximum amount of O_2. The oxygen signal was baseline corrected and normalized. The total charge was corrected for the background activity of the GC electrode, at which no (detectable) oxygen is formed. In the case of Co(H**L**), the experiment was performed four times to determine an average faraday efficiency and error (standard deviation over four measurements). The Faraday efficiency was calculated as follow;

The theoretical amount of moles of O_2 formed:

$$\text{moles } O_{2\,\text{theoretical}} = \frac{Q_{\text{total}} - Q_{\text{blank}}}{n * F}$$

In which Q_{total} is the total charge in C, Q_{blank} is the total charge in the blank solution in C, n is the number of electrons required to form one molecule of O_2, which is 4 in this case and F is the Faraday constant of 96485 C/mol.

The formed amount of moles of O_2 formed was determined:

$$\text{moles } O_{2\,\text{detected}} = \%O_2 * [O_2]_{\text{max}} * V$$

In which $\%O_2$ is the amount of O_2 formed in solution relative to the 1.1 mM calibration value, $[O_2]_{max}$ is the maximum solubility of O_2 in the electrolyte in M, which is $1.1 \cdot 10^{-3}$ M in this case and V is the volume of the electrochemical cell in L, which is 0.0080 L in this case.

The Faradaic efficiency was calculated as followed:

$$\text{Faradaic Effiency} = \frac{\text{moles } O_{2_{\text{detected}}}}{\text{moles } O_{2_{\text{theoretical}}}} * 100\%$$

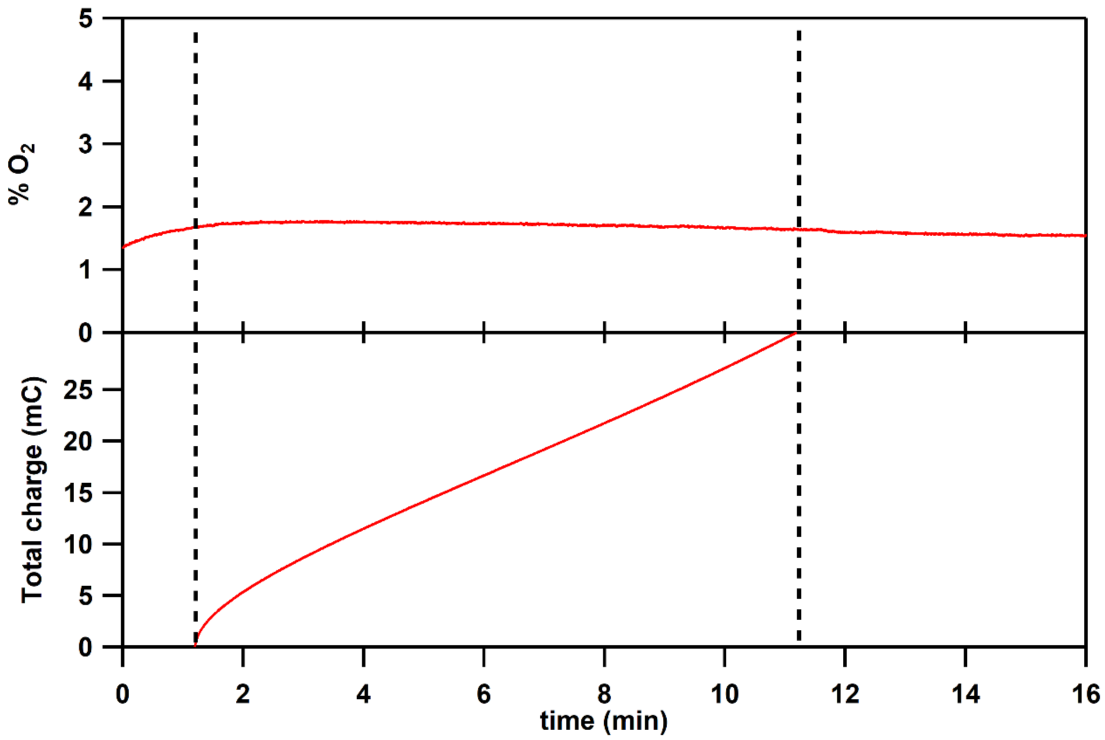

Figure S41. Oxygen signal during 10 minutes of chronoamperometry at 1.49 V vs. NHE of a 100 mM pH 7 phosphate blank solution. Top: % of O_2 in solution. Bottom: total charge during chronoamperometry at 1.49 V vs. NHE. The dotted lines indicate the time period in which potential was applied. Data is not baseline corrected or normalized. GC, Au and Ag/AgCl were used as WE, CE and RE, respectively.

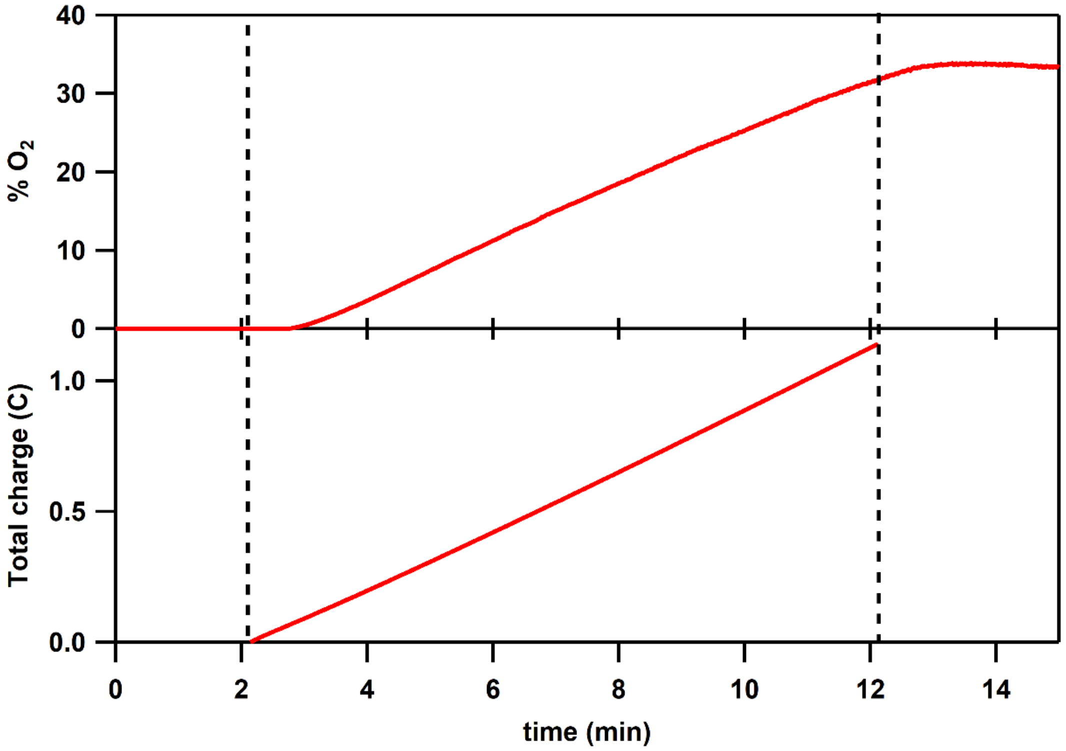

Figure S42. Oxygen signal during 10 minutes of chronoamperometry at 1.49 V vs. NHE of a 0.5 mM $Co(NO_3)_2$ in 100 mM pH 7 phosphate solution (= Co-Pi). Top: % of O_2 in solution relative to 1.1 mM O_2. Bottom: total charge during chronoamperometry at 1.49 V vs. NHE. The dotted lines indicate the time period in which potential was applied. Data is baseline corrected and normalized. GC, Au and Ag/AgCl were used as WE, CE and RE, respectively.

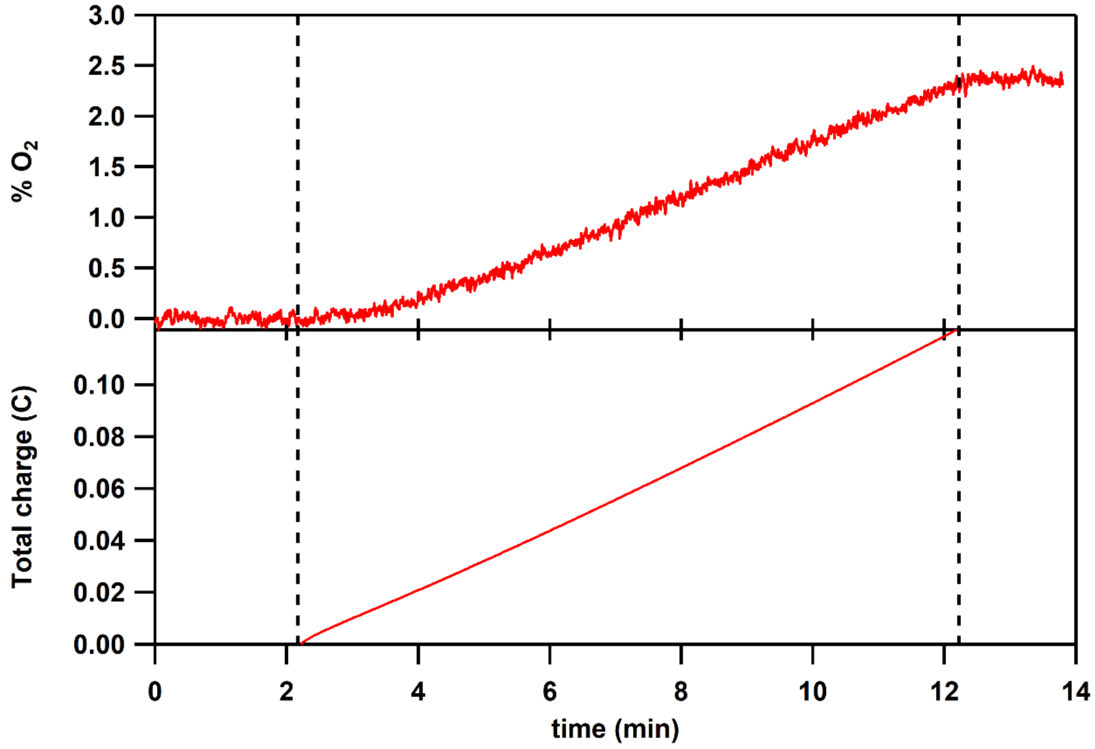

Figure S43. Oxygen signal during 10 minutes of chronoamperometry at 1.49 V vs. NHE of a 0.5 mM Co(H**L**) in 100 mM pH 7 phosphate solution. Top: % of O_2 in solution relative to a 1.1 mM O_2 solution. Bottom: total charge during chronoamperometry at 1.49 V vs. NHE. The dotted lines indicate the time period in which potential was applied. Data is baseline corrected and normalized. GC, Au and Ag/AgCl were used as WE, CE and RE, respectively.

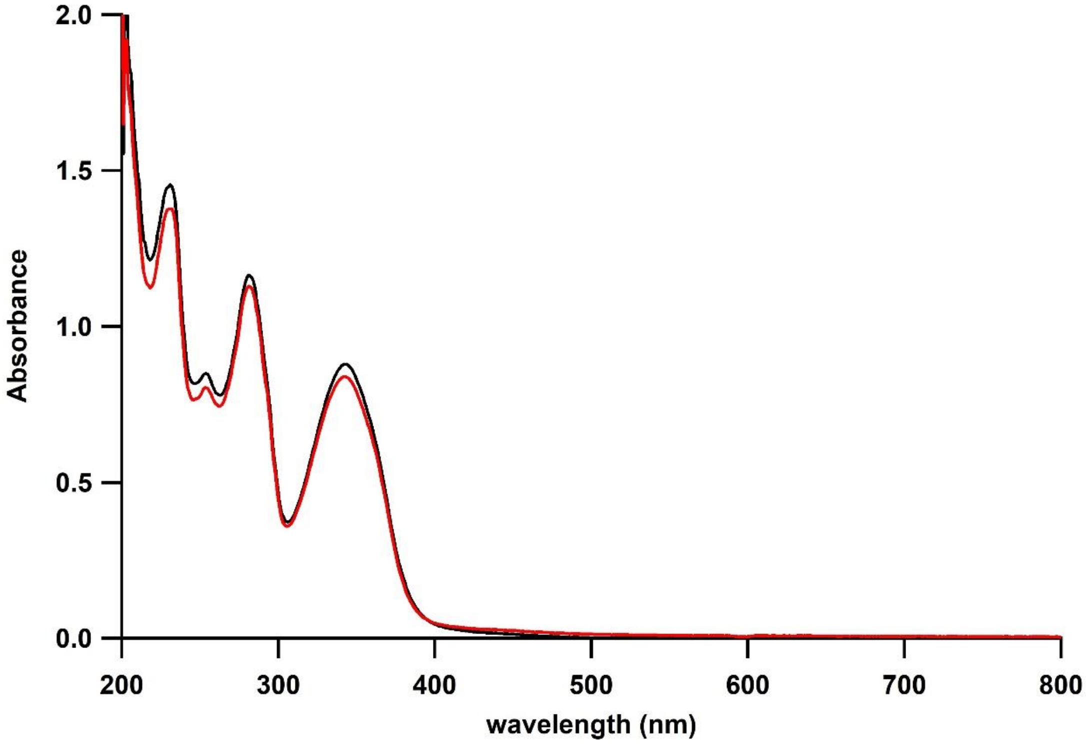

Figure S44. UV-vis spectra of Co(H**L**) in pH 7 phosphate buffer recorded before (black) and after (red) 5 hours of chronoamperometry at 1.49 V vs. NHE in a 0.5 mM Co(H**L**) in 100 mM pH 7 phosphate solution. UV-vis samples were diluted 10 times to obtain a suitable absorbance.

VII. XPS analysis of the electrode surface

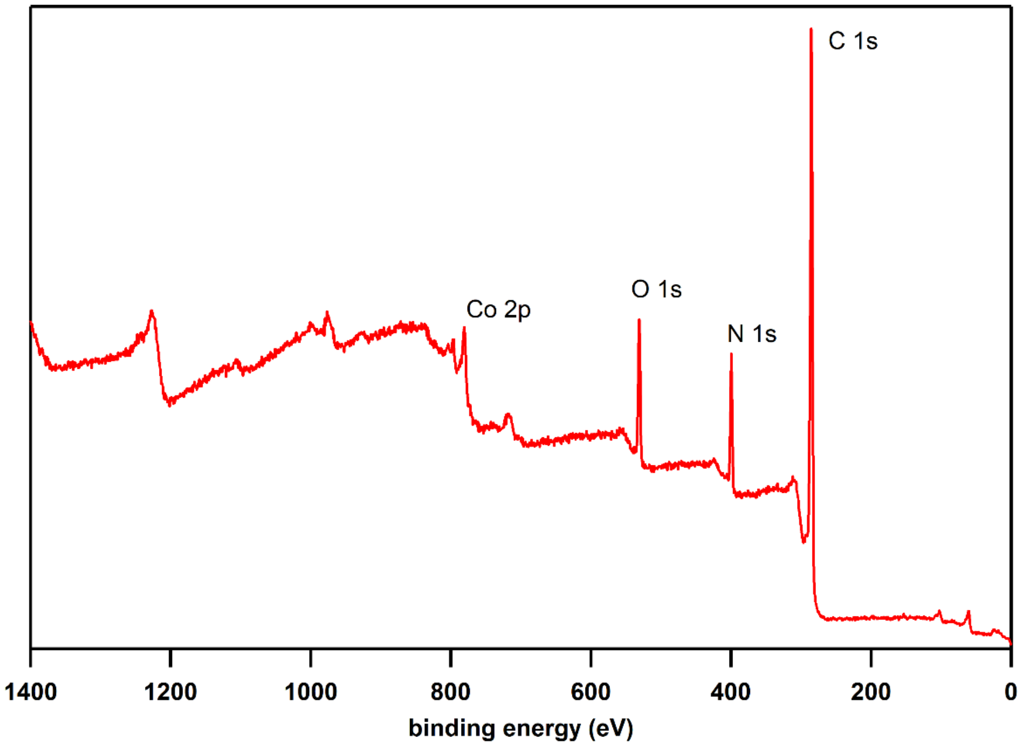

Figure S45. XP survey spectrum of powder reference [Co(H**L**)(OAc)$_2$]

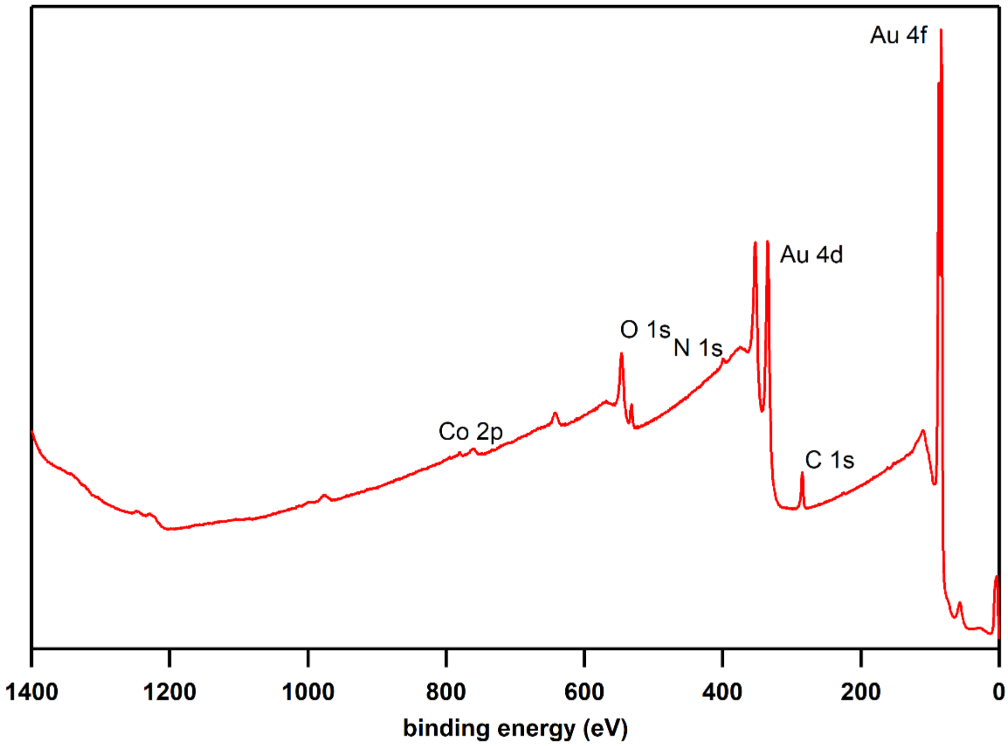

Figure S46. XP survey spectrum of Co(H**L**) on an Au electrode surface after 50 cycles between 1.1 and 1.6 V vs. NHE in a 100 mM pH 7 phosphate buffer at a scan rate of 100 mV/s.

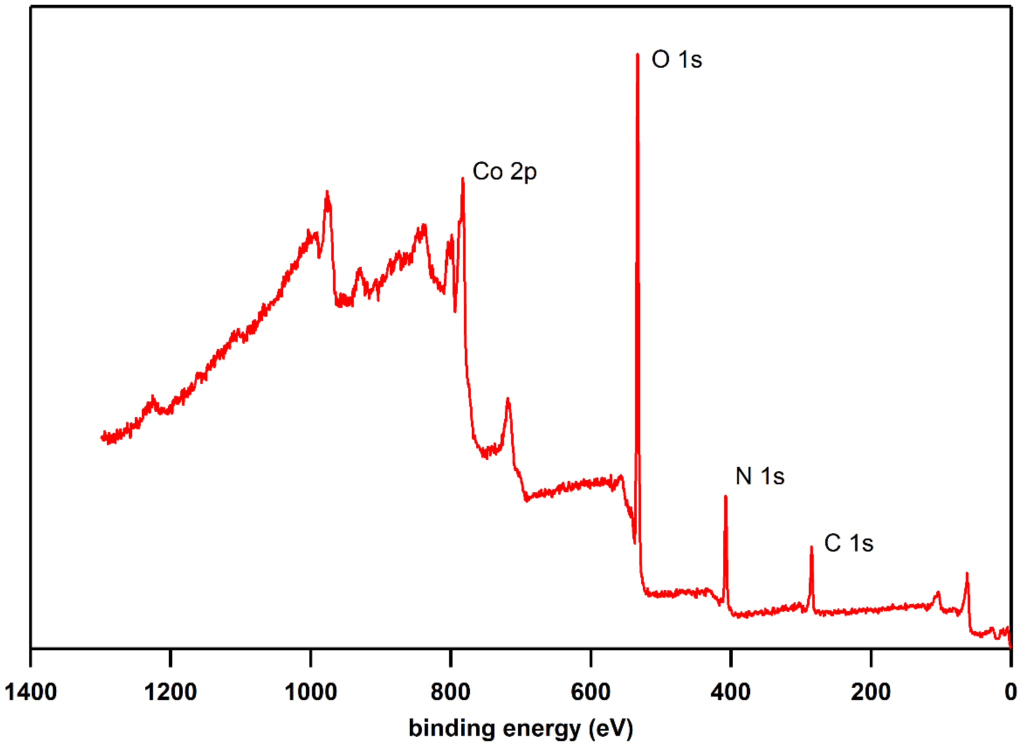

Figure S47. XP survey spectrum of powder reference $Co(NO_3)_2 \cdot 6\ H_2O$.

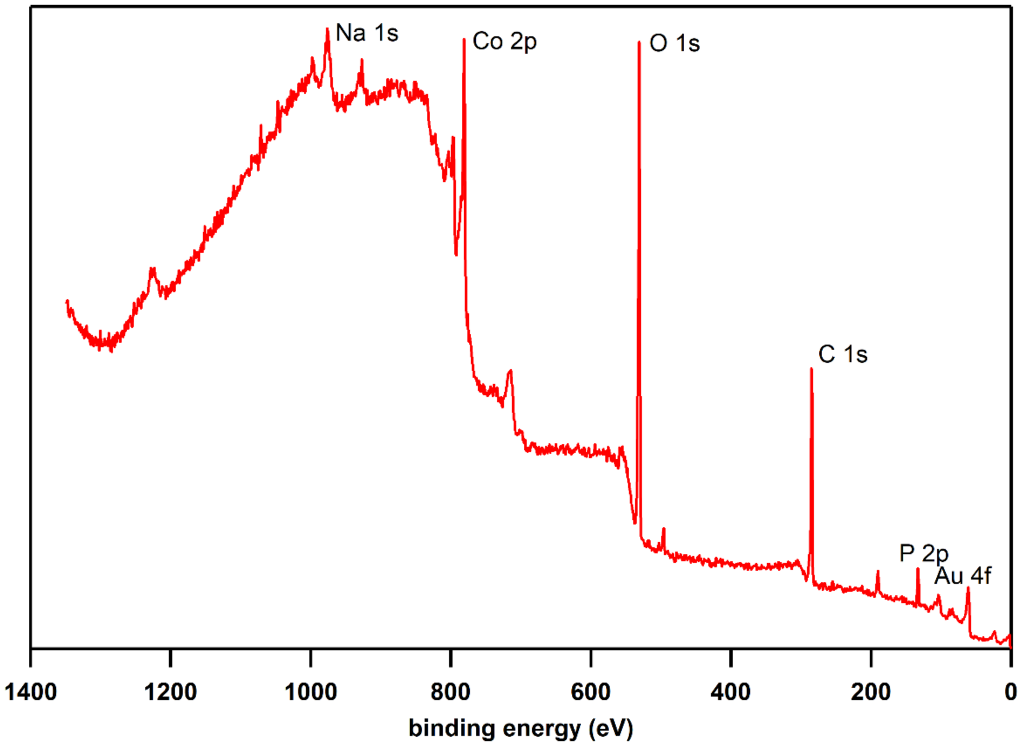

Figure S48. XP survey spectrum of Co-Pi on an Au electrode surface after 50 cycles between 1.1 and 1.6 V vs. NHE in a 100 mM pH 7 phosphate buffer at a scan rate of 100 mV/s.

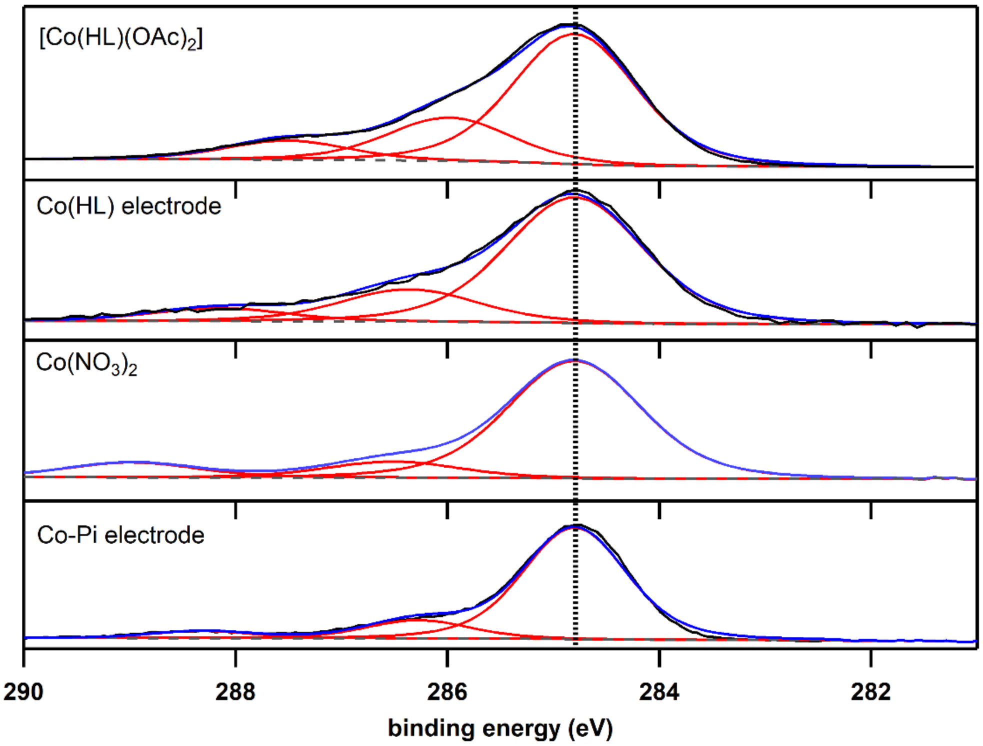

Figure S49. XP spectra of the C 1s region utilized for binding energy calibration setting the C1s component of sp^3 carbon to 284.8 eV. The XP spectra (black), components (red), sum of components (blue) and background (dashed grey) are shown for each sample. From top to bottom: 1) powder reference [Co(H**L**)(OAc)$_2$], 2) Co(H**L**) on an Au electrode surface after 50 cycles between 1.1 and 1.6 V vs. NHE in a 100 mM pH 7 phosphate buffer at a scan rate of 100 mV/s, 3) powder reference $Co(NO_3)_2 \cdot 6\ H_2O$, 4) Co-Pi on an Au electrode surface after 50 cycles between 1.1 and 1.6 V vs. NHE in a 100 mM pH 7 phosphate buffer at a scan rate of 100 mV/s.

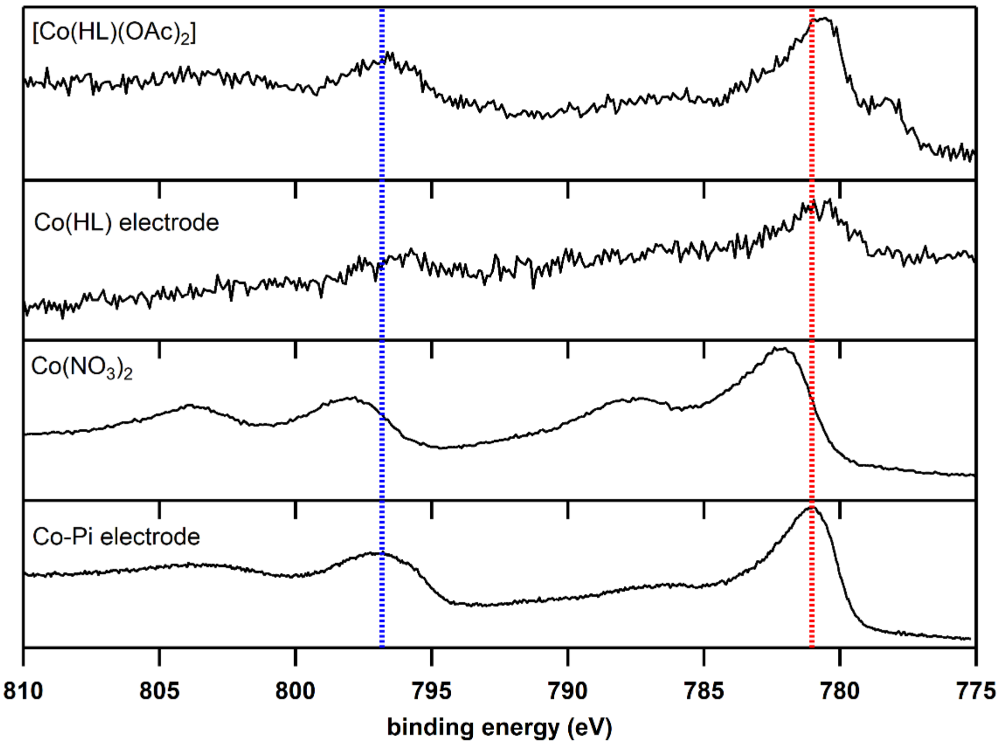

Figure S50. XP spectra of the Co 2p region including the Co $2p_{3/2}$ satellite peak. From top to bottom: 1) powder reference [Co(H**L**)(OAc)$_2$], 2) Co(H**L**) on an Au electrode surface after 50 cycles between 1.1 and 1.6 V vs. NHE in a 100 mM pH 7 phosphate buffer at a scan rate of 100 mV/s, 3) powder reference $Co(NO_3)_2 \cdot 6\ H_2O$, 4) Co-Pi on an Au electrode surface after 50 cycles between 1.1 and 1.6 V vs. NHE in a 100 mM pH 7 phosphate buffer at a scan rate of 100 mV/s. The red and blue line corresponds to the binding energy of the Co $2p_{3/2}$ and satellite peak in the Co-Pi sample.

Table S2. Quantification of data for XPS samples [Co(H**L**)(OAc)$_2$] and Co(H**L**) on the electrode.

Sample	Core level	Position (eV)	FWHM (eV)	Library RSF[10]	raw area	% at conc
[Co(H**L**)(OAc)2]	N 1s	399.5	1.82	1.8	703	86.9
	Co $2p_{3/2}$	781.0	2.61	12.6	802	13.1
Co(H**L**) electrode	N 1s	399.5	1.37	1.8	1020	86.2
	Co $2p_{3/2}$	780.5	3.34	12.6	1238	13.8

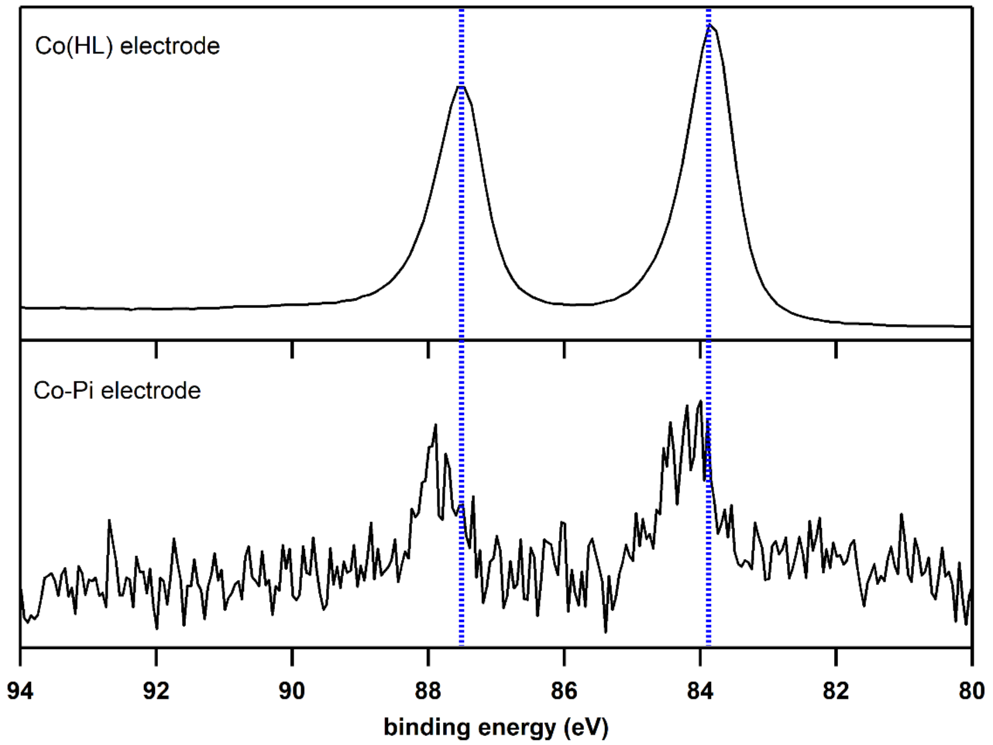

Figure S51. XP spectra of the Au 4f region. From top to bottom: 1) Co(H**L**) on an Au electrode surface after 50 cycles 1.1 and 1.6 V vs. NHE in a 100 mM pH 7 phosphate buffer at a scan rate of 100 mV/s, 2) Co-Pi on an Au electrode surface after 50 cycles between 1.1 and 1.6 V vs. NHE in a 100 mM pH 7 phosphate buffer at a scan rate of 100 mV/s.

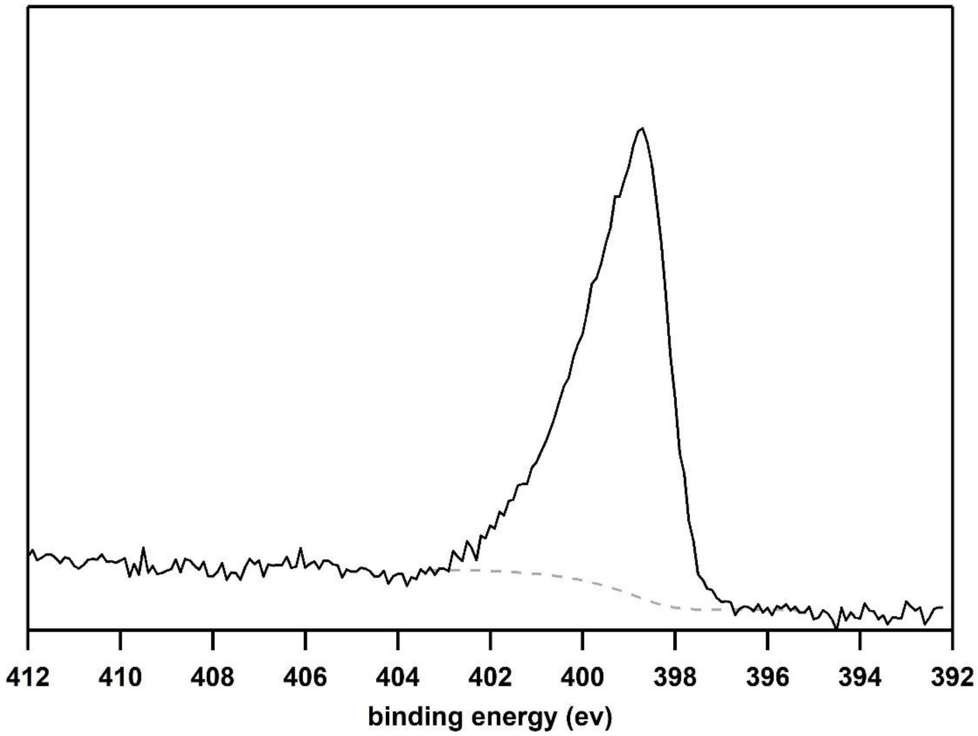

Figure S52. XP spectra of the N 1s region of the powder reference H**L** ligand (black) and background (grey dashed). The signal peak is asymmetric and suffers from tailing to higher BE, which is due to charging of the non-conductive H**L** ligand.

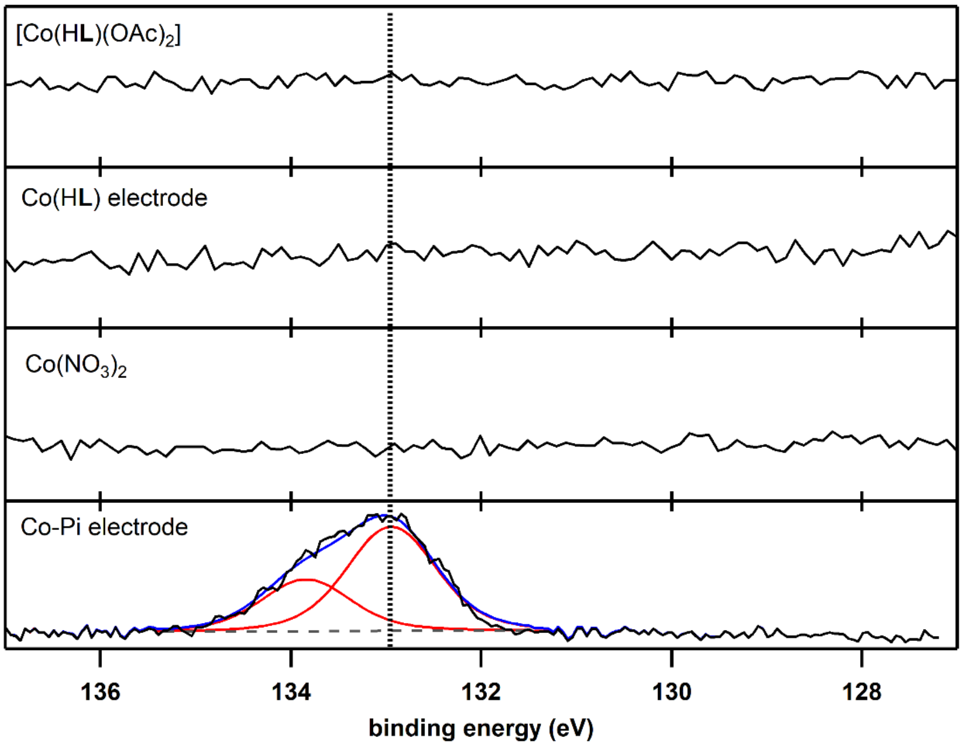

Figure S53. XP spectra P 2p signal. The XP spectra (black), components (red), sum of components (blue) and background (dashed grey) are shown for each sample. From top to bottom: 1) powder reference [Co(H**L**)(OAc)$_2$], 2) Co(H**L**) on an Au electrode surface after 50 cycles between 1.1 and 1.6 V vs. NHE in a 100 mM pH 7 phosphate buffer at a scan rate of 100 mV/s, 3) powder reference Co(NO$_3$)$_2$ · 6 H$_2$O, 4) Co-Pi on an Au electrode surface after 50 cycles between 1.1 and 1.6 V vs. NHE in a 100 mM pH 7 phosphate buffer at a scan rate of 100 mV/s.

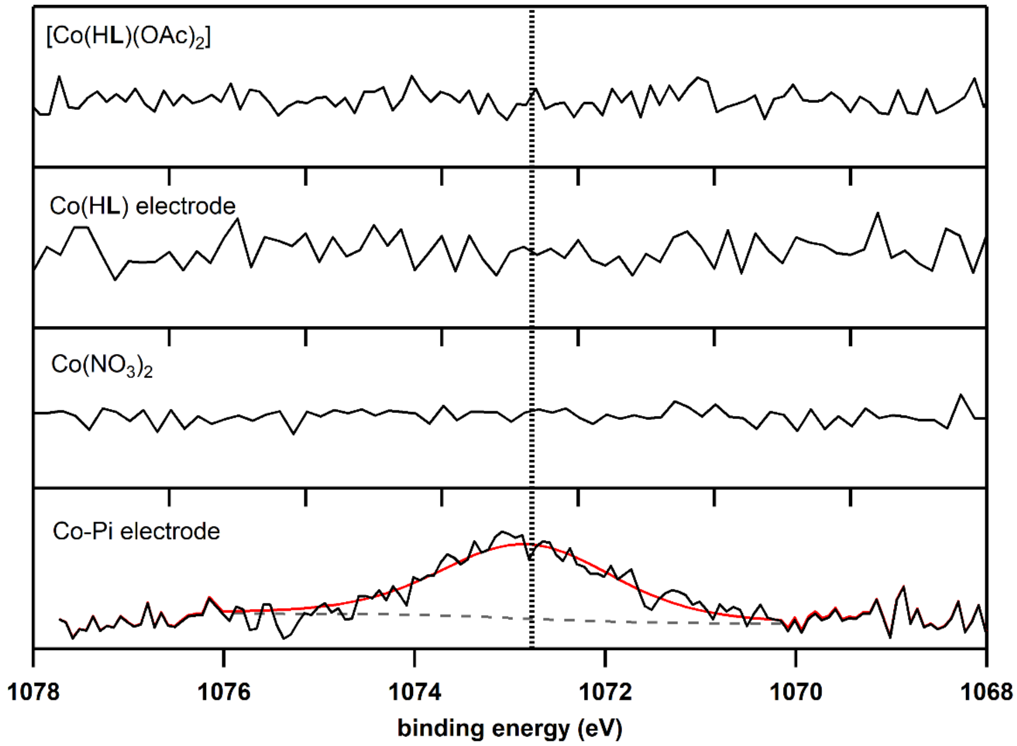

Figure S54. XP spectra Na 1s signal. The XP spectra (black), components (red) and background (dashed grey) are shown for each sample. From top to bottom: 1) powder reference [Co(H**L**)(OAc)$_2$], 2) Co(H**L**) on an Au electrode surface after 50 cycles between 1.1 and 1.6 V vs. NHE in a 100 mM pH 7 phosphate buffer at a scan rate of 100 mV/s, 3) powder reference Co(NO$_3$)$_2$ · 6 H$_2$O, 4) Co-Pi on an Au electrode surface after 50 cycles between 1.1 and 1.6 V vs. NHE in a 100 mM pH 7 phosphate buffer at a scan rate of 100 mV/s.

VIII. NMR spectra

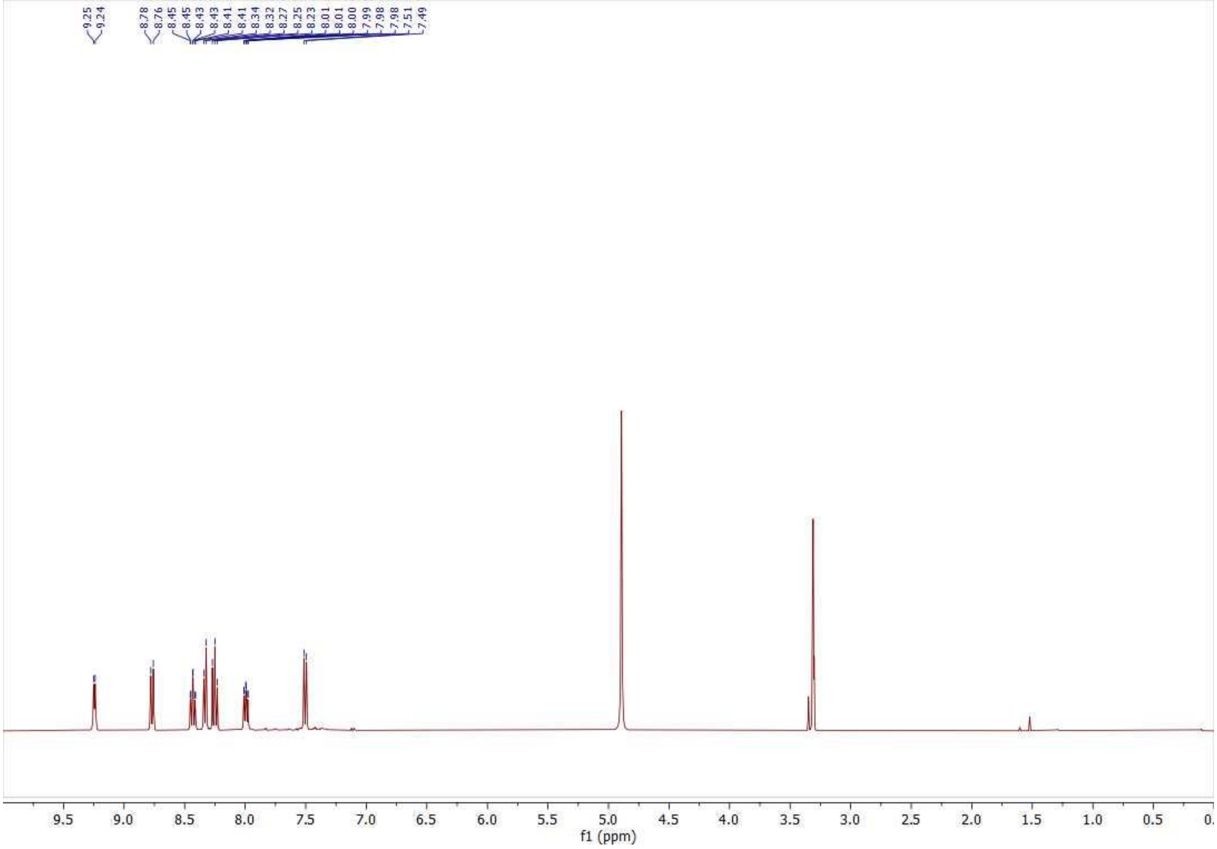

Figure S55. ^1H NMR of [Zn(H**L**)]$(OTf)_2$ in methanol-d_4 (400 MHz).

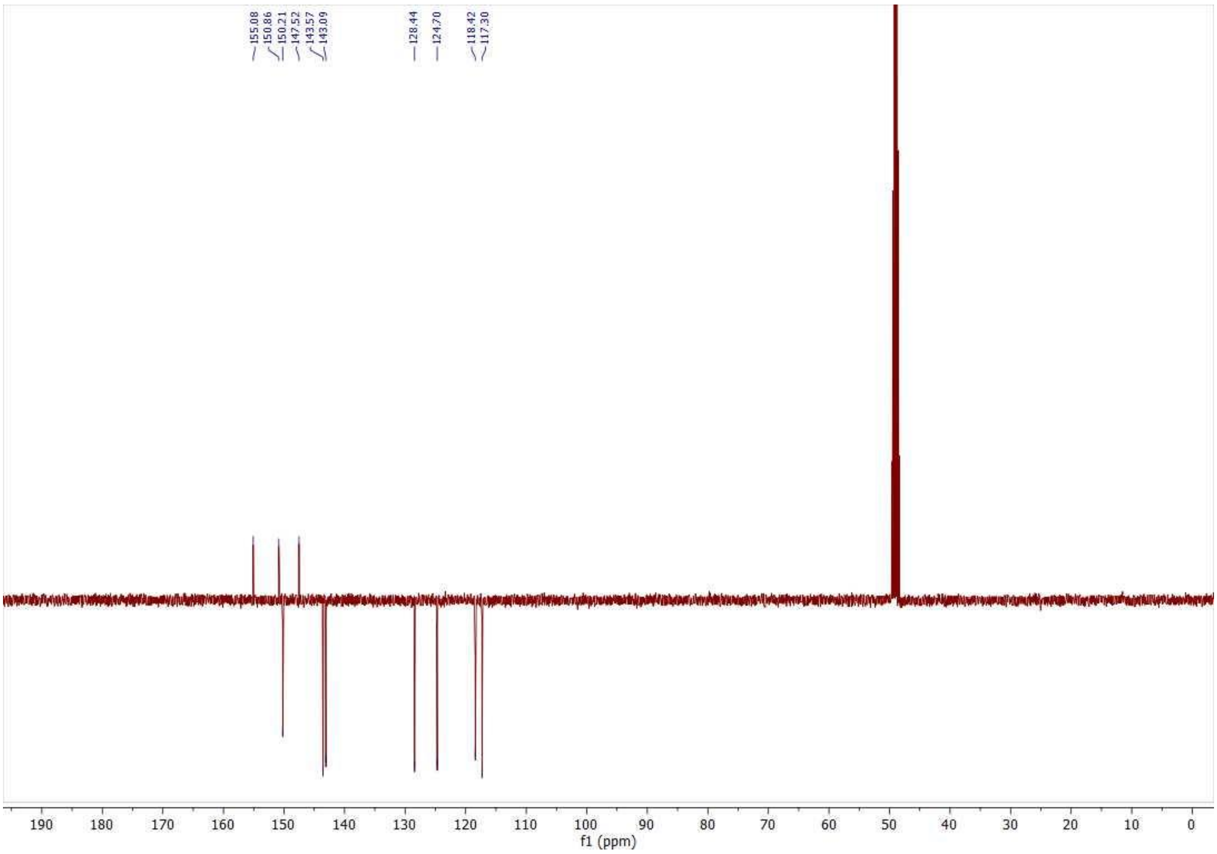

Figure S56. ^{13}C APT NMR of [Zn(H**L**)](OTf)$_2$ in methanol-d$_4$ (101 MHz).

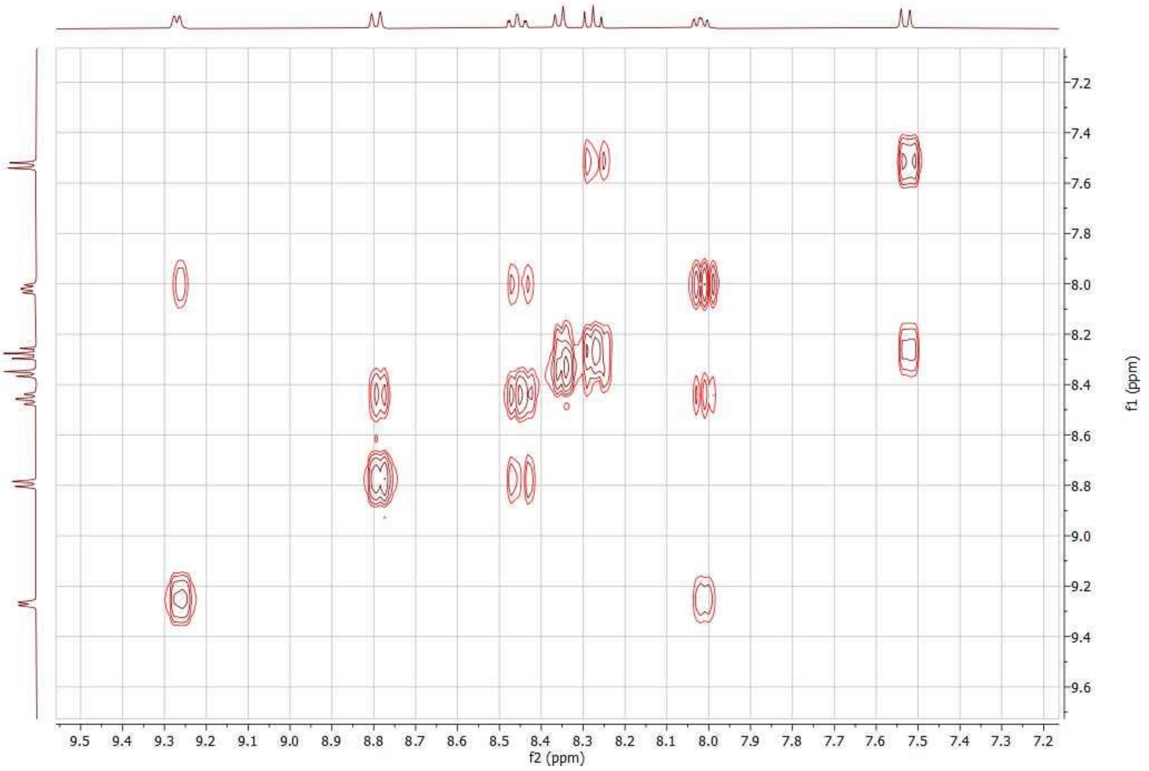

Figure S57. ^{1}H COSY NMR of [Zn(H**L**)](OTf)$_2$ in methanol-d$_4$ (400 MHz).

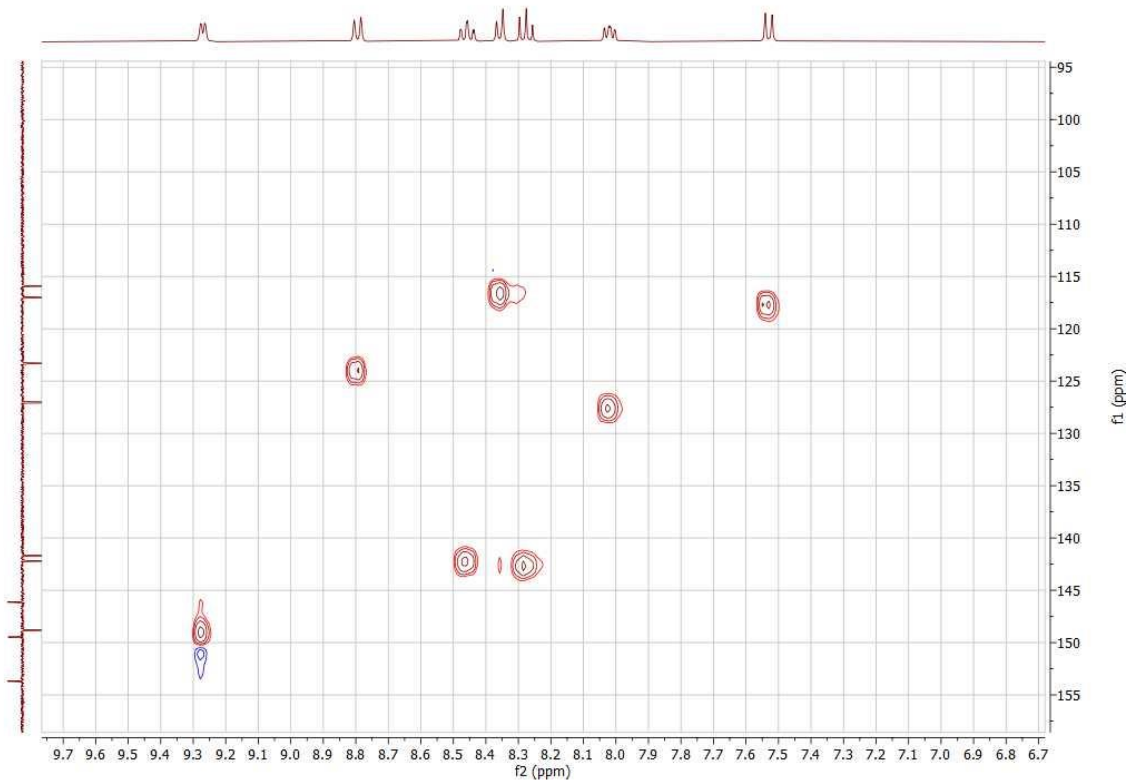

Figure S58. $^{1}H^{13}C$ HSQC NMR of [Zn(H**L**)]$(OTf)_2$ in methanol-d_4 (400 MHz).

IX. Mass spectrometry

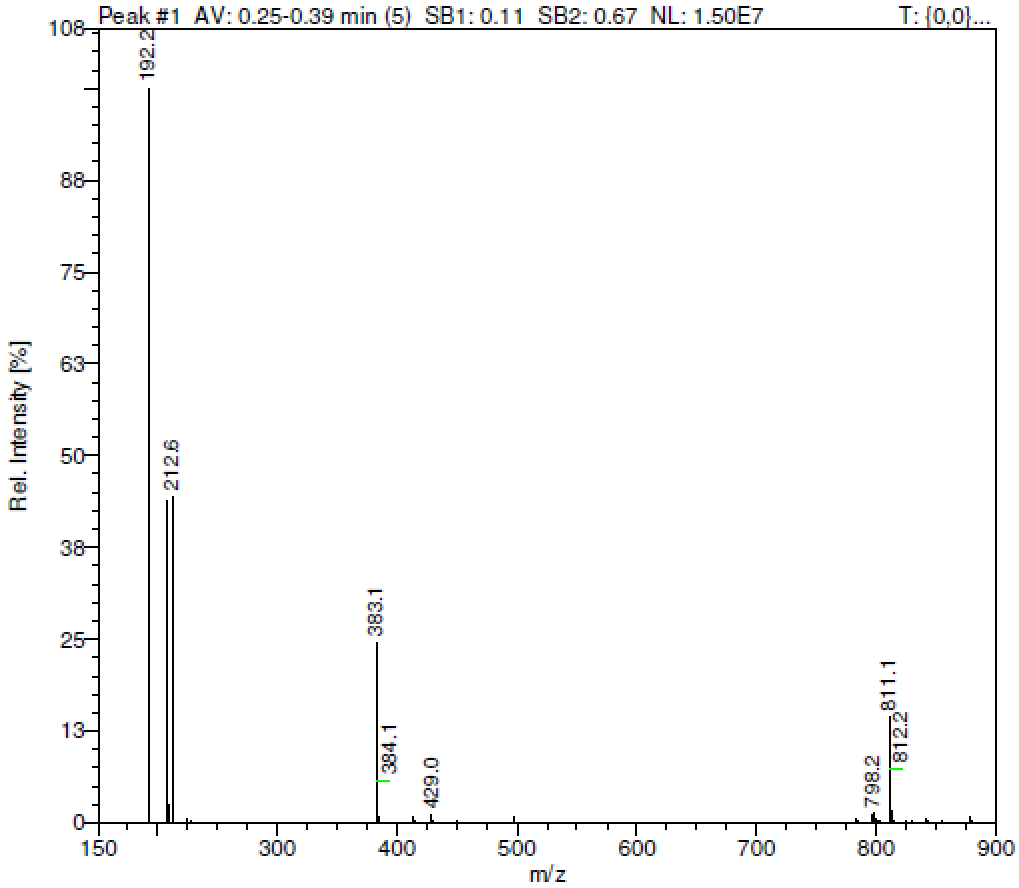

Figure S59. MS spectrum of a solution of [Co(H**L**)(OAc)$_2$] in MeOH

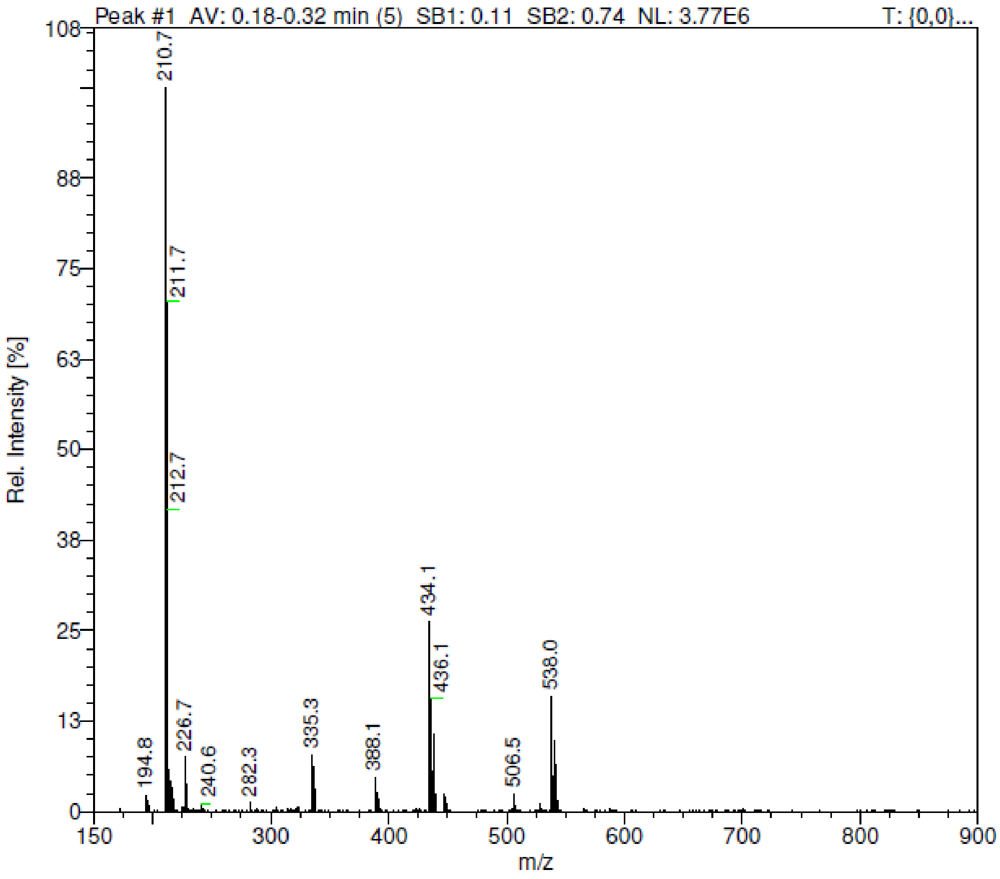

Figure S60. MS spectrum of a solution of [Zn(H**L**)(OTf)$_2$] in MeOH

X. References

1. Joachim Demnitz, F. W.; D'Heni, M. B., A high yielding preparation of 2,2'-bipyridine-1-oxide. *Org. Prep. Proced. Int.* **1998,** *30*, 467-469.
2. Yin, J.; Xiang, B.; Huffman, M. A.; Raab, C. E.; Davies, I. W., A General and Efficient 2-Amination of Pyridines and Quinolines. *J. Org. Chem.* **2007,** *72*, 4554-4557.
3. Smith, A. J.; Kalkman, E. D.; Gilbert, Z. W.; Tonks, I. A., ZnCl2 Capture Promotes Ethylene Polymerization by a Salicylaldiminato Ni Complex Bearing a Pendent 2,2'-Bipyridine Group. *Organometallics* **2016,** *35*, 2429-2432.
4. Zheng, S.; Reintjens, N. R. M.; Siegler, M. A.; Roubeau, O.; Bouwman, E.; Rudavskyi, A.; Havenith, R. W. A.; Bonnet, S., Stabilization of the Low-Spin State in a Mononuclear Iron(II) Complex and High-Temperature Cooperative Spin Crossover Mediated by Hydrogen Bonding. *Chem. -Eur. J.* **2016,** *22*, 331-339.
5. Sheldrick, G. M., SHELXT–Integrated space-group and crystal-structure determination. *Acta Crystallogr. A* **2015,** *71*, 3-8.
6. Fulmer, G. R.; Miller, A. J. M.; Sherden, N. H.; Gottlieb, H. E.; Nudelman, A.; Stoltz, B. M.; Bercaw, J. E.; Goldberg, K. I., NMR Chemical Shifts of Trace Impurities: Common Laboratory Solvents, Organics, and Gases in Deuterated Solvents Relevant to the Organometallic Chemist. *Organometallics* **2010,** *29*, 2176-2179.
7. Sauerbrey, G., Verwendung von Schwingquarzen zur Wägung dünner Schichten und zur Mikrowägung. *Z. Phys* **1959,** *155*, 206-222.
8. Metrohm Autolab B.V., Autolab application note EC05, EQCM Study of Underpotentially Deposited (UPD) Lead Adlayer on Gold; 2011.
9. Langerman, M.; Hetterscheid, D. G. H., Fast Oxygen Reduction Catalyzed by a Copper(II) Tris(2-pyridylmethyl)amine Complex through a Stepwise Mechanism. *Angew. Chem. Int. Ed.* **2019,** *58*, 12974-12978.
10. Wagner, C. D.; Taylor, J. A., Reference levels in electron-excited and X-ray-excited Auger spectra. *Surf. Interface Anal.* **1979,** *1*, 73-73.
